# The dynamic linkage between covid-19 and nutrition: a review from a probiotics perspective using machine learning and bibliometric analysis

**DOI:** 10.3389/fnut.2025.1575130

**Published:** 2025-05-09

**Authors:** Christos Stefanis, Christina Tsigalou, Ioanna Bezirtzoglou, Gregoria Mitropoulou, Chrysoula Voidarou, Elisavet Stavropoulou

**Affiliations:** ^1^Laboratory of Hygiene and Environmental Protection, Department of Medicine, Democritus University of Thrace, Alexandroupolis, Greece; ^2^School of Chemistry, University of Edinburgh, Edinburgh, United Kingdom; ^3^Laboratory of Applied Microbiology and Biotechnology, Department of Molecular Biology and Genetics, Democritus University of Thrace, Alexandroupolis, Greece; ^4^School of Agriculture, University of Ioannina, Arta, Greece

**Keywords:** COVID-19, SARS-CoV-2, bibliometrics, diet, probiotics, nutrition, ASReview

## Abstract

**Introduction:**

The pandemic crisis is now a memorable milestone in the history of science, not only for the impacts on the population’s health but also for the effort of the medical community to find immediate solutions amid the pandemic so that appropriate therapeutic means can be provided. Diet and nutrition could not fail to be studied in the context of combating the side effects of COVID-19. This study attempts to detect the relationship between dietary patterns and the disease of COVID-19 and emphasizes research on probiotics by mapping the knowledge produced during the pandemic until 2024.

**Methods:**

In addition to bibliometrics, a machine-learning framework, ASReview, was used to structure the literature search. With this method, 2,309 articles were collected from the PubMed database, with 599 constituting inputs into bibliometric software and further analysis.

**Results:**

Food choices, dietary patterns, vitamins and their role (vitamin D), obesity, and probiotics were keywords that attracted global research attention. Dietary supplements also constituted a field of study regarding the evolution of the disease and the impact they could have after the first pandemic wave.

**Discussion:**

Probiotics were considered an adjunct therapeutic intervention not only during the period before the development of vaccines but also alongside other therapeutic solutions. Whether used preventively or during the treatment phase, probiotics were studied to combat COVID-19 due to their potential role in immunomodulation and ability to regulate gut microbiota during respiratory infections.

## 1 Introduction

From the onset of the pandemic until November 10, 2024, more than 776.8 million confirmed cases of SARS-CoV-2, the virus responsible for COVID-19, and over 7 million confirmed deaths were reported to the WHO across 234 countries. Most COVID-19-related deaths occurred in 2020, 2021, and 2022. However, with increasing immunity, deaths have significantly decreased. According to the WHO, between October 14 and November 10, 2024, 77 countries reported new COVID-19 cases, resulting in 27 deaths worldwide during this period (1).

Compared with the first years of the pandemic (2020–2022), there are now more dispassionate conditions regarding the measures to deal with COVID-19, with several more defence mechanisms such as vaccines to deal with this disease that humanity has. In just a few years, COVID-19 has evolved into a pre-eminent pandemic crisis at a global level that has affected almost all political and economic aspects of human life (2).

Recent studies have linked the pandemic to climate change. Viral stability is affected by modifications that may alter the virus’s transmissibility, e.g., at high humidity levels or, conversely, at high temperatures. The frequency of people sneezing may change. Thus, there will be a difference in the viral load carried by air. Climate change also has an indirect impact on the transmission level through hosts, since bats and other mammals are known to be hosts to alpha-and beta-coronaviruses; gamma coronaviruses mainly infect birds (3). Moreover, delta coronaviruses infect birds and mammals. Furthermore, research showed the shifts that the global crisis brought to agriculture, livestock farming, and fishing at the level of distribution and transportation, quantities, sustainability and quality, and the safety of these foods (4, 5).

Government measures to limit coronavirus transmission, such as travel and trade restrictions and quarantines, brought about significant disruptions in the economic flaw, food production, demand and distribution, and altered dietary patterns (6–8). In addition to the apparent health security issues, the disruption caused by COVID-19 to food systems and supply chains has also brought to the fore the connection between nutrition and the pandemic, not only at the level of food security but also as a means of mitigating its effects, since it is known that nutrition, under certain conditions, can help during the progression and in mitigation of disease (9–11).

Additionally, a balanced diet, i.e., the presence of vitamins, minerals, antioxidants, and the absence of refined foods, among other things, can strengthen the human immune system and better respond to the symptoms caused by COVID-19 (12–16). Nutrients and micronutrients in diet patterns promote human health and well-being. By regulating the composition and function of the intestinal microflora, probiotics can also contribute to maintaining human health and adjunctively enhance other methods of treating the coronavirus (17, 18).

Although there is now a relatively large body of literature on COVID-19, from various sources and origins and research objectives, it is nevertheless essential to have studies that summarise specific fields of knowledge to capture the evolution of a cognitive field and its cognitive structure, as provided by bibliometric studies. Regarding the issue of nutrition and its impact on research on COVID-19, the PubMed database, which has predominantly medical content, does not have many studies, as far as we know. The first bibliometric study was published in 2021, considering a short research period of only 3 months, January–March 2020. It was not related to nutrition but only to the correlation of COVID cases with the population of countries and the production of research manuscripts (19, 20).

Three studies were published in the PubMed database during 2022. The first aimed, inter alia, to study probiotics as an adjunctive approach in patients with COVID-19 and wanted to reveal their role in regulating the intestinal flora of patients to reduce the intensity of symptoms and the duration of the disease. Furthermore, bibliometric techniques also showed the connection between diet and coronavirus. This particular study selected papers from January 2020 until December 2021 (17).

The following bibliometric analysis focused on the role of nutrition and COVID-19 as a reference period of January 1, 2020, and June 30, 2021. This research captured research related to food security, changes in dietary habits and patterns during the pandemic, and finally, the role of nutritional factors such as vitamins and minerals, as well as a balanced diet concerning the symptoms and dynamics of the disease (21).

The subsequent bibliometric analysis explored from January 2019 to September 2022 as a reference period and the absence of guidelines for nutritional supplements in preventing and treating COVID-19 as a starting point. The results showed that during that period, vitamins, mainly D and C, ferritin, oxidative stress and the uncertainty of the contribution of nutritional supplements to the pandemic crisis were research niches depicted in the literature (22).

In a 2023 publication, which includes the research production of articles on the coronavirus and nutrition from January 2020 to December 2022, it is observed that in the first months, the contribution of nutrition to the progression of the disease as a field of study was absent. Later, bibliometrics revealed the role of nutrition as a factor that may influence the progression of the disease or play a role in the predisposition of obese patients to the progression of the disease. Finally, the Mediterranean diet was a field of study regarding the duration and intensity of the symptoms of the disease (23).

Subsequently, this bibliometric study is unique in its scope and methodology compared to the existing studies in the PubMed database. It integrates machine learning-based article review (ASReview) and bibliometric tools to analyse the relationship between diet and COVID-19 comprehensively. In addition, an update of the existing knowledge and literature is necessary. Thus, a broader time frame and data are required; this study incorporates publications up to October 2024, capturing the most recent research trends and extending the analysis to a longer pandemic period. The current research will also highlight the role of probiotics in nutritional interventions and the management of COVID-19 disease. Furthermore, the broader period embodied by this initiative will provide a new, straightforward concept for future research directions and public health policies regarding possible upcoming pandemics. Overall, this bibliometric study will attempt to inform and broaden the understanding of the association between nutrition and COVID-19, setting a new benchmark for future bibliometric studies.

Unlike earlier bibliometric studies that covered limited timeframes or took a broader view of nutrition, this study offers a fresh and timely perspective by examining the entire course of the pandemic up to October 2024. Combining artificial intelligence and machine learning-assisted systematic review (ASReview) methodology with bibliometric analysis provides a more thorough and reliable picture of how research in this field has evolved. This dual approach helps capture emerging trends, particularly the growing interest in probiotics’ role in preventing and managing COVID-19. As we move through 2025, the need to revisit and update our understanding remains critical—especially with the ongoing challenges of long COVID and the focus on enhancing immune resilience. In this context, the present review not only brings together 5 years of research but also helps inform future nutrition and public health planning strategies for similar crises that may arise.

This bibliometric study will attempt to answer the following research questions: (1) How has global research activity on nutrition and COVID-19 been characterised over time regarding publication trends? (2) What are the dominant research themes and knowledge structures within the field of nutritional research related to COVID-19? (3) What highlights the role and relationship between probiotics and the management of COVID-19?

## 2 Materials and methods

### 2.1 Literature review process

A mixed method was followed regarding the stage of article screening, the selection, and the collection of bibliographic metadata for the bibliometric analysis. Initially and after collecting the articles from the PubMed database, an innovative article screening tool, ASReview, was selected, which promotes speed and transparency during the architecture and execution of a systematic review.^1^. This specific tool, Active Learning for Systematic Reviews, ASReview version 0.19.2, uses machine learning algorithms to help the researcher sort the most relevant articles during the review selection. As in machine learning, its structure is based on the choice of the appropriate classifier for the two categories, relevant and irrelevant articles, which the researcher is asked to name at the initial stage of the ASReview operation to begin distinguishing the articles into these two categories. Therefore, each time the researcher classifies a manuscript in the relevant category, ASReview reclassifies the entire collection of articles by promoting the most relevant ones at the beginning of the list, resulting in the immediate and straightforward researcher’s selection for those documents that are in line with their research topic and at the same time, the most irrelevant articles will appear at the end of the relevant list (24–27). Prioritising articles in this way while training the model based on the researcher’s annotations results in the model’s improved ability to predict the relevance of articles continuously. When enough annotations are completed or the user decides to stop, the process is completed, and the final set of relevant records is obtained. This specific tool has been proven to save up to 60% of the time required in the manuscript screening step (28–30).

Specifically, the tool uses an active machine learning algorithm to rank articles with a high to low probability of eligibility for final selection. Before the AI tool can select relevant articles, its algorithm must be trained with at least one relevant and one irrelevant article (i.e., prior knowledge). In our case, two relevant and one irrelevant articles were selected since the more prior knowledge, the better the algorithm is trained, and the faster it will find relevant articles. After training with prior knowledge, the AI tool ranked all untagged articles (i.e., articles whose eligibility has not yet been decided) from the highest to lowest probability of being relevant.

The default parameters of ASReview (TF-IDF, Naive Bayes, Maximum query strategy, and Dynamic Resampling) are optimised for speed and performance and applied in this study. The Feature Extraction Technique defines how features are extracted from the dataset. In this case, TF-IDF (Term Frequency-Inverse Document Frequency) is used, a common technique in text analysis that weighs the importance of a term in a document relative to a collection (corpus). The machine learning algorithm used to classify or predict based on the extracted features is set to Naive Bayes, which is a probabilistic model often used for text classification due to its simplicity and effectiveness. The Query Strategy: The method used to select the most informative samples to query (label) in an active learning process. The strategy here is Maximum, referring to selecting samples with the highest uncertainty or importance based on a specific criterion. The Balance Strategy considers how the data is balanced to avoid biases during training. The option is Dynamic Resampling (Double), a strategy that dynamically resamples the dataset to ensure class balance or diversity during model training.

The mixed method presented here, described for the first time, comprises three main steps. First, ASReview was employed to screen articles using an AI-based tool. Second, the “human factor” was introduced, characterised by the decision not to halt the selection process after encountering a predefined number of consecutive irrelevant documents. Third, the resulting body of literature was utilised for bibliometric analysis.

**Figure 1:**
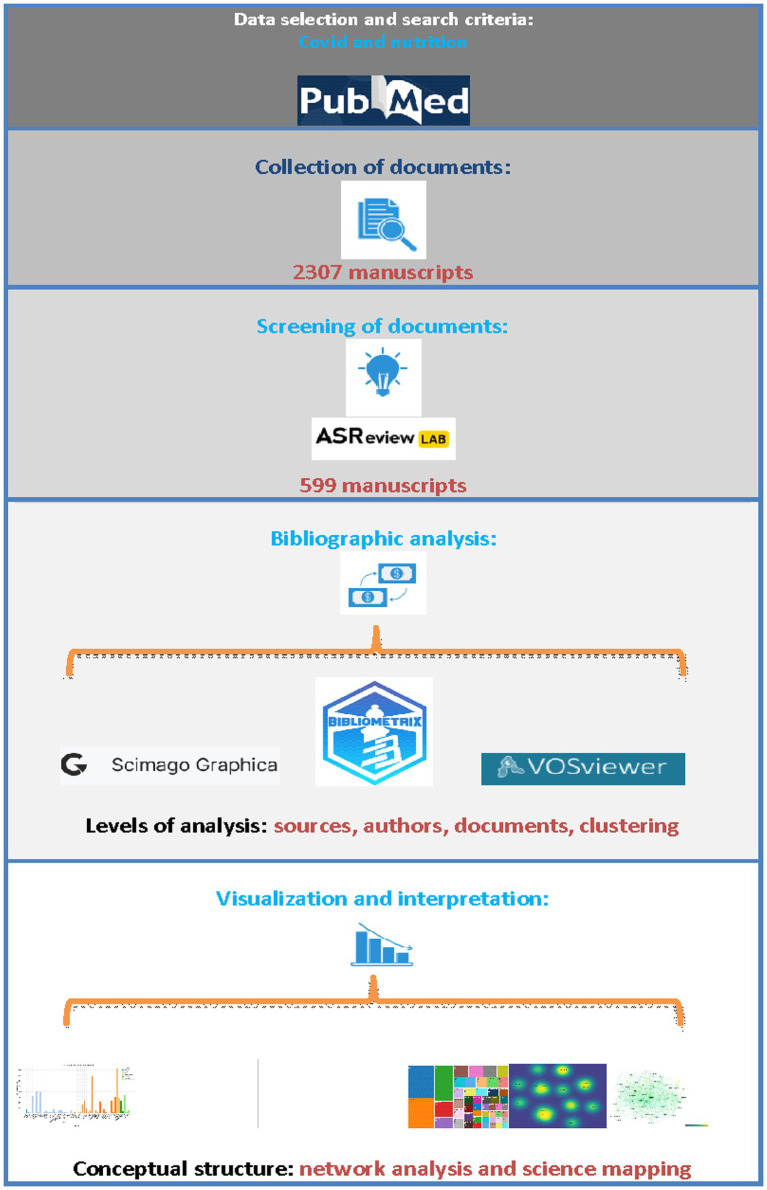
Workflow of the bibliometric review process.

In the sorting stage, articles were filtered based on their titles and abstracts without applying a mathematical stopping criterion for the machine learning phase. Specifically, the selection process was not terminated after observing, for instance, 20 consecutive irrelevant articles. Instead, all selected articles were screened, resulting in the termination of the process only after 76 successive irrelevant articles, as illustrated in Figure 1. The bibliographic metadata of the selected articles were subsequently used for bibliometric analysis.

### 2.2 Bibliometric analysis

For this study, a search was executed in the PubMed database. PubMed^®^ is a vast resource hosting over 37 million biomedical literature citations and abstracts, including MEDLINE content and life science journals. Introduced in 1996, the PubMed database is managed by the National Centre for Biotechnology Information (NCBI), a part of the U.S. National Library of Medicine (NLM) at the National Institutes of Health (NIH). Its primary goal is to aid in locating and accessing biomedical and life sciences literature to advance health globally. Moreover, PubMed focuses on citations in biomedicine and health sciences and related fields like life sciences, behavioural sciences, chemistry, and bioengineering.

After preliminary experimentation with keyword combinations, the keywords “COVID and nutrition” were initially used to collect as many articles as possible. The combination of these keywords also serves to combine the articles that will be included to approach the topic of nutrition about COVID in a multifaceted way, namely the role of food, diet, dietary choices, habits and choices, nutritional interventions, as well as individual foods, bioactive substances, supplementation and beneficial microorganisms. The search criteria were: (“sars cov 2”[MeSH Terms] OR “sars cov 2”[All Fields] OR “covid”[All Fields] OR “covid 19”[MeSH Terms] OR “covid 19”[All Fields]) AND (“nutrition s”[All Fields] OR “nutritional status”[MeSH Terms] OR (“nutritional”[All Fields] AND “status”[All Fields]) OR “nutritional status”[All Fields] OR “nutrition”[All Fields] OR “nutritional sciences”[MeSH Terms] OR (“nutritional”[All Fields] AND “sciences”[All Fields]) OR “nutritional sciences”[All Fields] OR “nutritional”[All Fields] OR “nutritionals”[All Fields] OR “nutritions”[All Fields] OR “nutritive”[All Fields]). In addition, after mapping the bibliographic networks and presenting the bibliometric indicators, the research will elucidate the role of probiotics from the articles used in the bibliometric analysis.

The selection, screening and final collection of articles consists of 4 stages. In the initial stage, all articles from the PubMed database^2^ were entered into the bibliographic citation software Mendeley^3^ to detect possible duplicates, further removal and formation of the final body of the bibliography. In the second stage, the open-source machine learning tool ASReview was used to screen the articles at the title and abstract level, as described above. In the third stage, the bibliographic metadata of the selected documents was used as input data for bibliographic analysis in two free software programs, VOSviewer 1.6.20^2^ and bibliometrix 4.1^3^, of the R programming language (31–33). In the final stage, SCImago Graphica and the software mentioned above were all applied to produce and extract figures, tables, and graphs and interpret the data. The figure below depicts the streamlined process of selecting the database, formulating search criteria, collecting the document, and applying bibliometric tools and data interpretation (Figure 1).

The analysis focused on specific document types, including Clinical Trials, Meta-Analyses, Randomised Controlled Trials, Reviews, and Systematic Reviews. No time frame was set since the literature was anchored to the pandemic’s emergence. The search was conducted on October 31st, 2024, and all the manuscripts were collected that day to avoid extra publication bias. The collection included only English-language manuscripts organised in an Excel spreadsheet.

We used VOS Viewer (v1.6.20), SCImago Graphica and R Bibliometrix (v4.4.0) to analyse and map the bibliographic data. Co-occurrence analysis was performed to identify recurring keywords in the manuscripts’ titles, abstracts, and texts. A co-authorship analysis was also carried out using the full counting method, which gives equal weight to each collaboration. The bibliometric analysis included a streamlined flow: defining research criteria and questions and selecting features (publication year, subject area, institutional affiliations, keyword analysis, and research trends). The data were processed using the above software, creating bibliographic maps, networks, and interpretations of the results (34, 35).

## 3 Results

**Figure 2:**
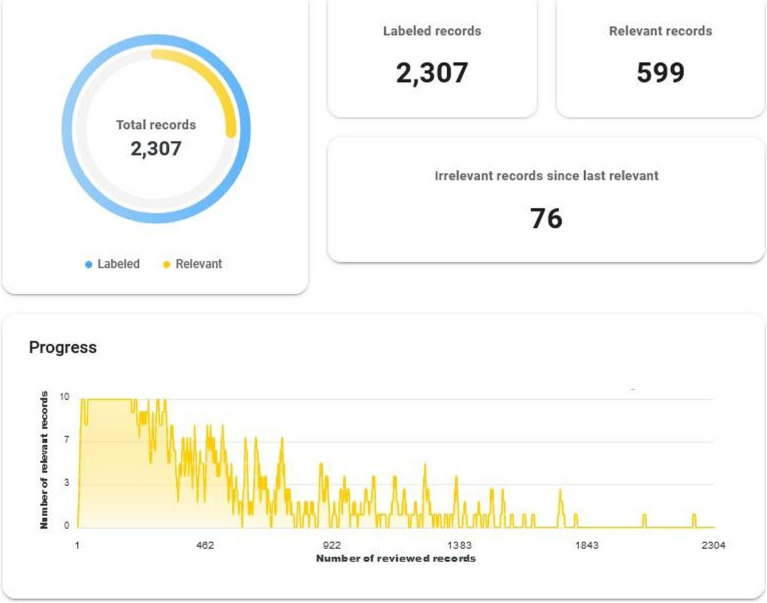
ASReview results.

The initial search in the PubMed database returned 2,307 manuscripts. Figure 2 illustrates the number of identified manuscripts labelled as relevant at any point during the AI-supported screening process with the ASReview (Supplementary Figure 1; Figure 2). After screening the documents and sorting them at the title and abstract level with ASReview, the final number exploited for the bibliometric analysis was 599 (Supplementary Table 1). The details of the papers at the text type level and the date of appearance in the collection are given below.

**Figure 3:**
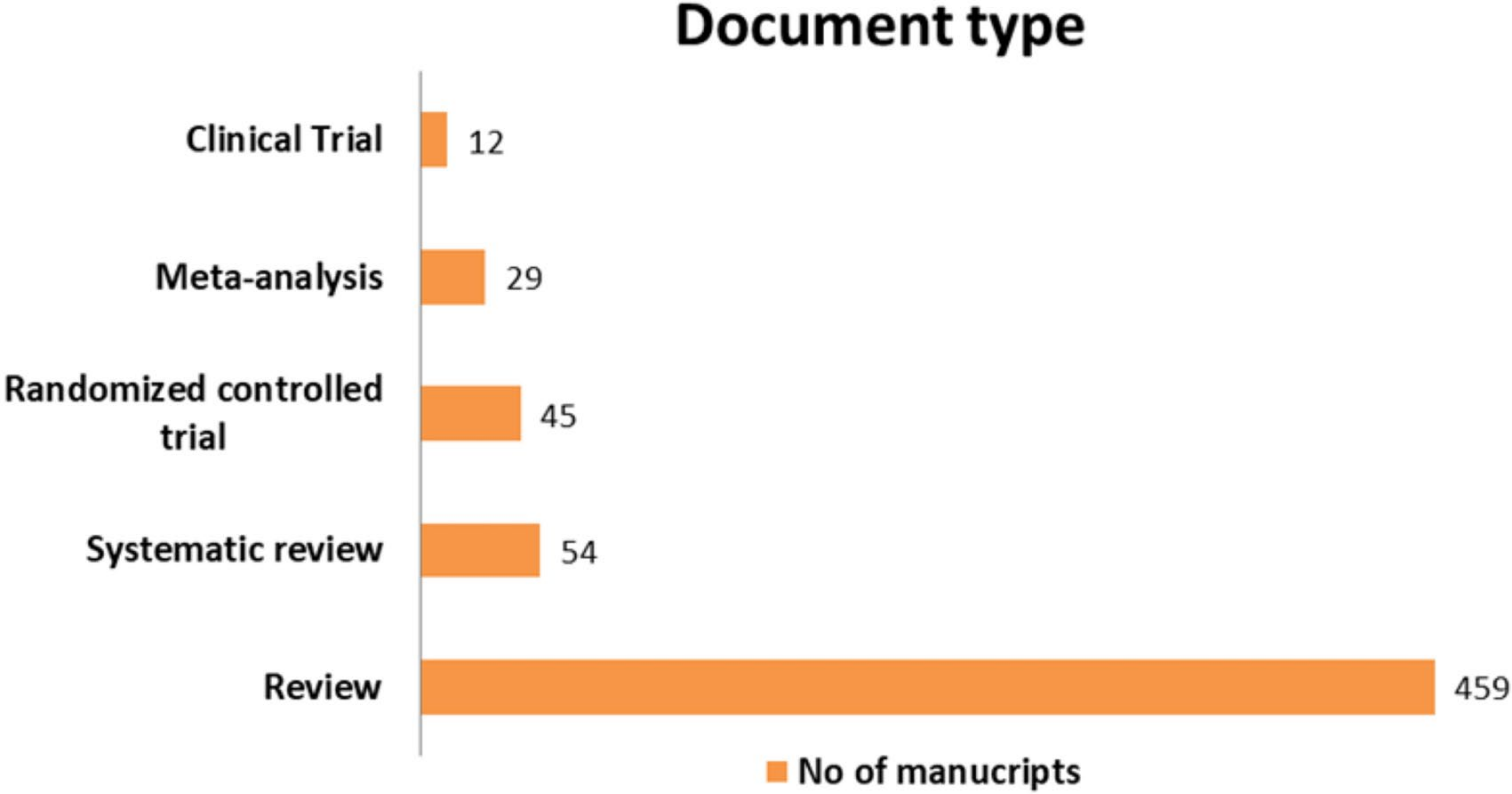
Number of manuscripts per document type.

Figure 3 provides insights regarding the number of documents per year and type within the document collection. Review and systematic review articles are the largest category, indicating the value of synthesising and presenting existing knowledge and addressing specific research questions. The number of interventions, meta-analyses and clinical trials fluctuates between 12 and 45 studies, suggesting the contribution of experimental and analytical research initiatives. Considering the annual distribution of the manuscripts, a pattern influenced by the global pandemic is observed. These patterns demonstrate the scientific community’s adaptability and resilience in responding to evolving global priorities.

**Figure 4:**
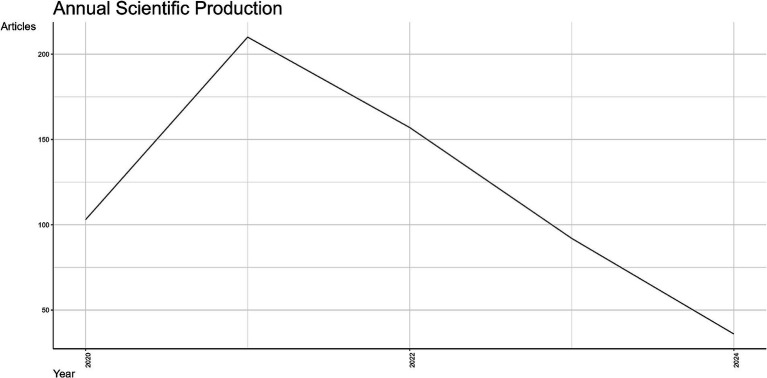
Annual scientific production related to nutrition and COVID-19 (2020–2024).

The annual scientific production is shown in Figure 4. The y-axis corresponds to the number of articles published, while the x-axis corresponds to years. The trends of scientific production from 2020 to 2024 reveal diverse patterns in publication output. In particular, in the initial year of 2020, the article production began with over 100 manuscripts, reflecting an initial response to emerging global health challenges. A significant surge occurred in 2021, peaking at approximately 200 articles, likely driven by intensified research efforts during the COVID-19 pandemic. This peak was followed by a steady decline starting in 2022, with article counts dropping below 150. The downward trend continued through 2023 and 2024, reaching the lowest publication count of fewer than 50 articles. It must be underlined that the reduction in 2024 is partially explained because this year is not fully covered in the document collection. However, the decline in research publications may be attributed to reduced urgency in pandemic-related research and shifting funding priorities. Additional analysis suggests that this publication trend was influenced by the global shift in research priorities, with early pandemic years seeing intense investigation into immediate health challenges, followed by diversification into broader health issues and sustainability topics.

Co-authorship analysis reveals that at least two researchers authored 558 documents, while 6,8% of the documents, namely 41, were published by only one researcher. In addition, the collaboration of authors, as indicated by the average number of authors per article, is 6, pinpointing a strong tendency toward collaborative research and multi-author contributions across the document collection.

**Figure 5:**
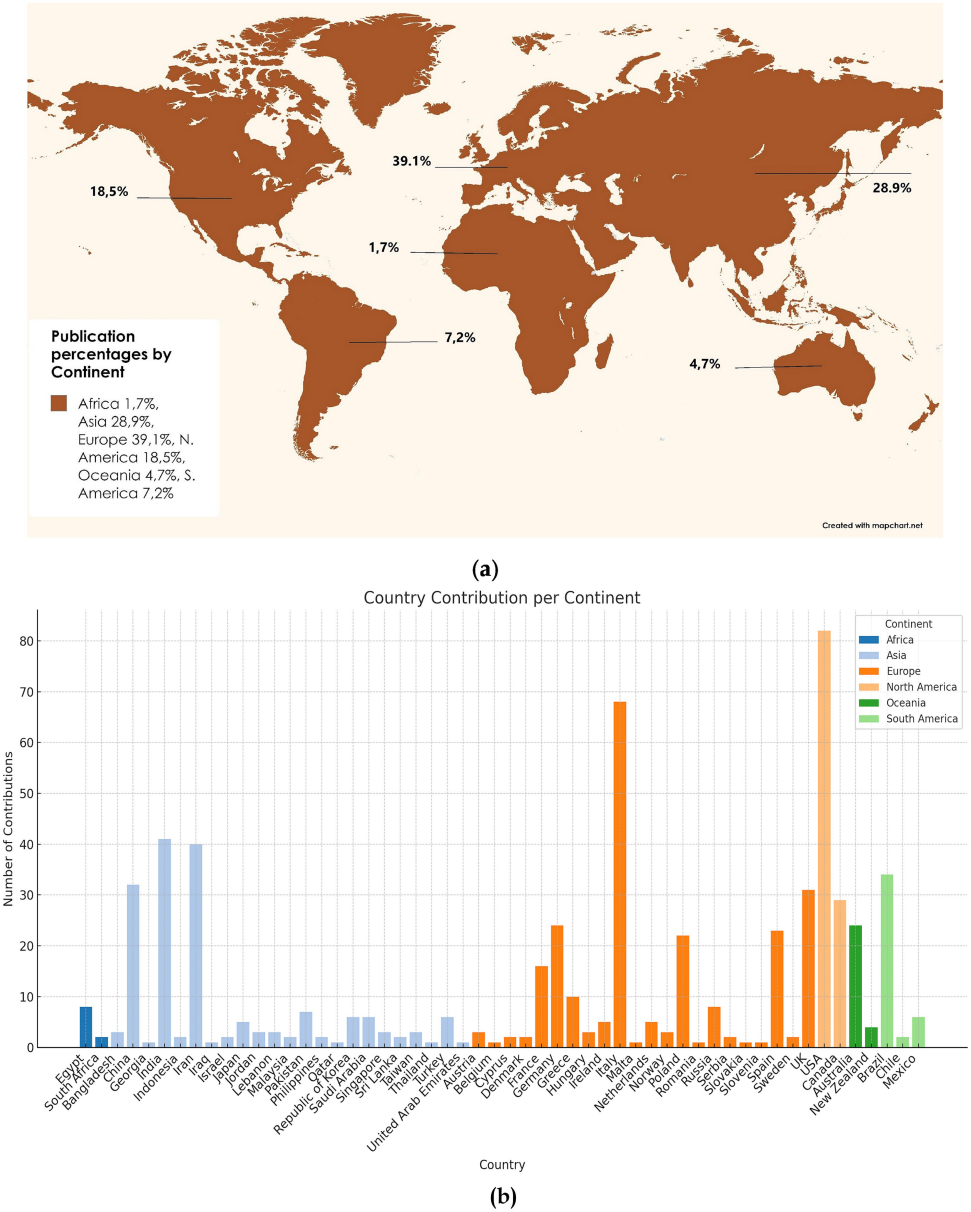
Frequency word clouds of top 50 words in **(a)** publication percentages by continent; **(b)** country contribution per continent.

Figure 5 presents a global overview of publication contributions, combining a world map in panel (a) with a detailed bar chart in panel (b). Panel (a) visualises the percentage distribution of publications across continents using a global projection. Europe is the leading contributor, accounting for 39.1% of the total output, followed by Asia at 28.9% and North America at 18.5%. South America contributes 7.2%, Oceania 4.7%, while Africa shows the lowest contribution at 1.7%. This panel highlights the dominance of Europe, Asia, and North America in global research output while underscoring the comparatively lower contributions from Africa and Oceania.

Panel (b) represents the number of contributions, namely documents per country for each continent. China and India have the highest publication outputs in Asia, while countries such as Japan, Saudi Arabia, and Iran contribute smaller shares. Europe shows a strong presence from the UK, Italy, and Germany, with notable contributions from France, Spain, and Netherlands. The USA, Canada, and Mexico dominate North America’s output. Brazil is the primary contributor in South America, with smaller shares from Chile and Argentina. Oceania’s contributions are led primarily by Australia, while New Zealand plays a minor role. Africa’s publication output is modest, with Egypt and South Africa as the main contributors.

As shown in Figure 5, specific regions such as Europe, Asia, and North America are at the forefront of the research landscape in the worldwide publication trends. The bar chart highlights disparities within continents, where a few countries dominate contributions. Subsequently, continents like Africa, Oceania, and South America, although with a less represented scientific fingerprint, still disclose significant contributions from leading countries such as Egypt, Australia, and Brazil. This particular output signifies the diversified scientific and geographical imbalance while illustrating the dominant influence of specific regions and countries in the global research landscape.

**Figure 6:**
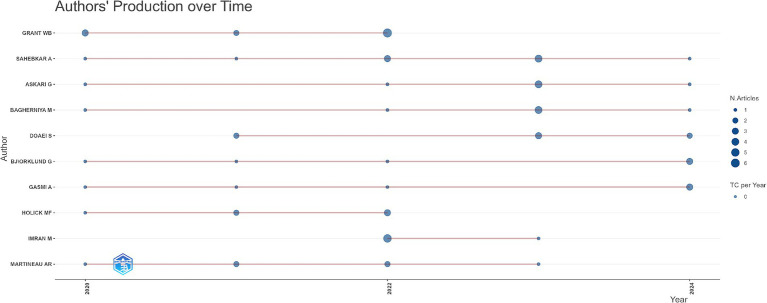
Authors’ production over time.

The productivity of authors from 2020 to 2024 is shown in Figure 6. The horizontal axis represents the years 2020 to 2024. The authors’ names are listed on the y-axis, while the horizontal axis highlights their respective publication activity over time. Each dot along the timeline represents an article published by the author in that specific year, with the dots varying to illustrate the number of manuscripts published; the bigger the dot, the more documents the researcher published that year. Thin horizontal lines linking dots correspond to periods of constant publication activity, revealing varying degrees of productivity among researchers. Authors such as Grant W.B. maintained consistent publication activity with notable peaks in specific years. On the contrary, Doaei S. and Holick M.F. exhibited concentrated bursts of productivity followed by limited output. Sahebkar A., Askari G., and Baghernya M. demonstrated sporadic publication patterns, while Bjorklund G. and Martineau A.R. maintained steady but lower contributions. This indicates diverse engagement levels and evolving research priorities among key contributors.

**Figure 7:**
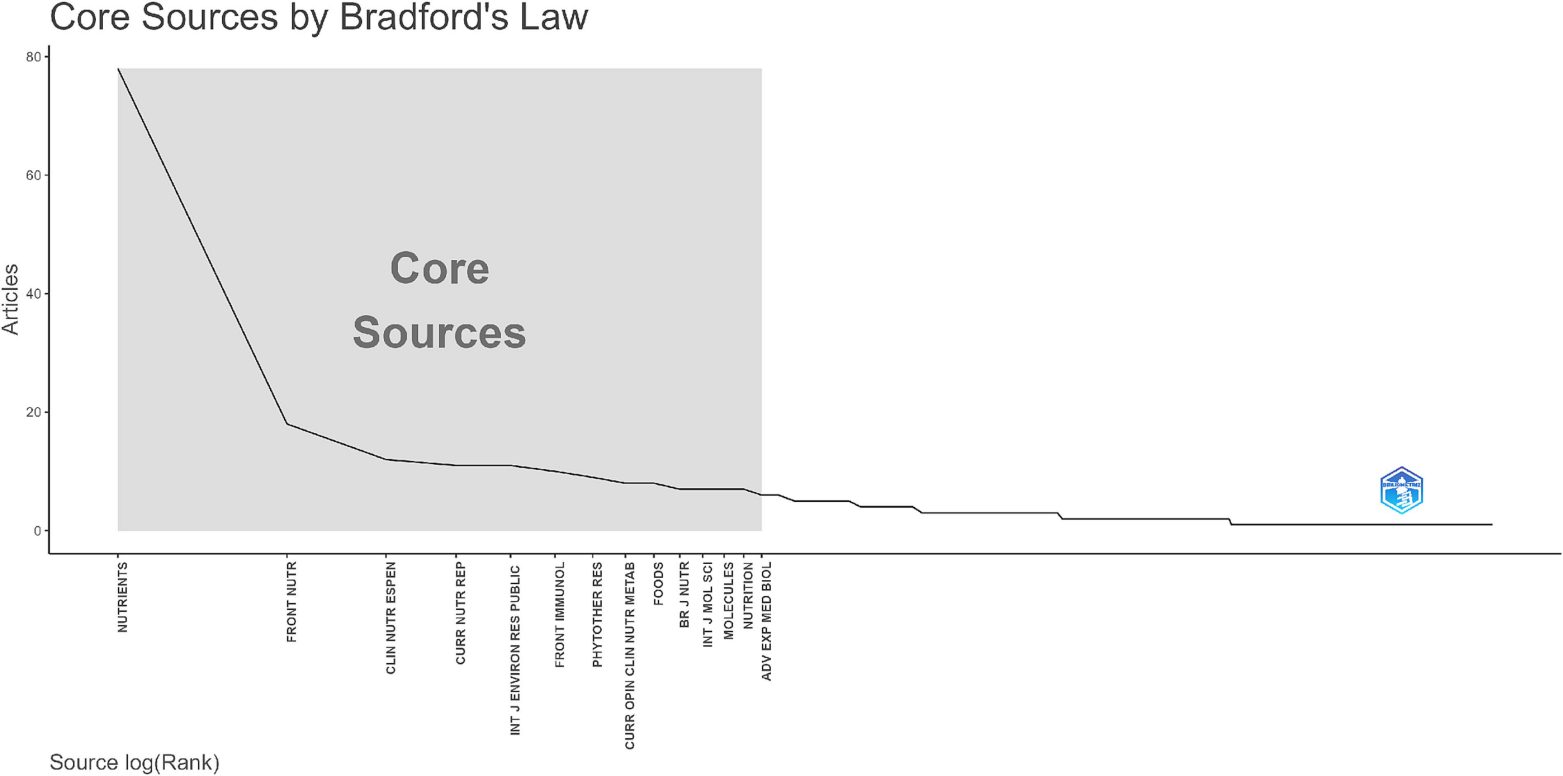
Bradford’s Law distribution of journal sources.

According to Bradford’s Law, illustrated in Figure 7, the distribution of journal articles underlines a concentration of publications within a few core sources, namely 14 scientific journals. “Nutrients” journal emerged as the leading journal with approximately 80 published documents, designating its vital role in publishing pandemic-related nutrition research. Other journals such as “Frontiers in Nutrition,” “Clinical Nutrition ESPEN,” and “Current Nutrition Reports” contributed relatively low. Five scientific journals had below ten publications, according to Figure 4. Overall, the sharp decline in article counts as journal rank increases demonstrates the expected Bradford’s Law distribution, emphasising the dominance of a few prolific journals and concurrently their specialised focus on nutrition and public health, positioning them as preferred journals for publishing COVID-19-related studies, among others.

**Figure 8:**
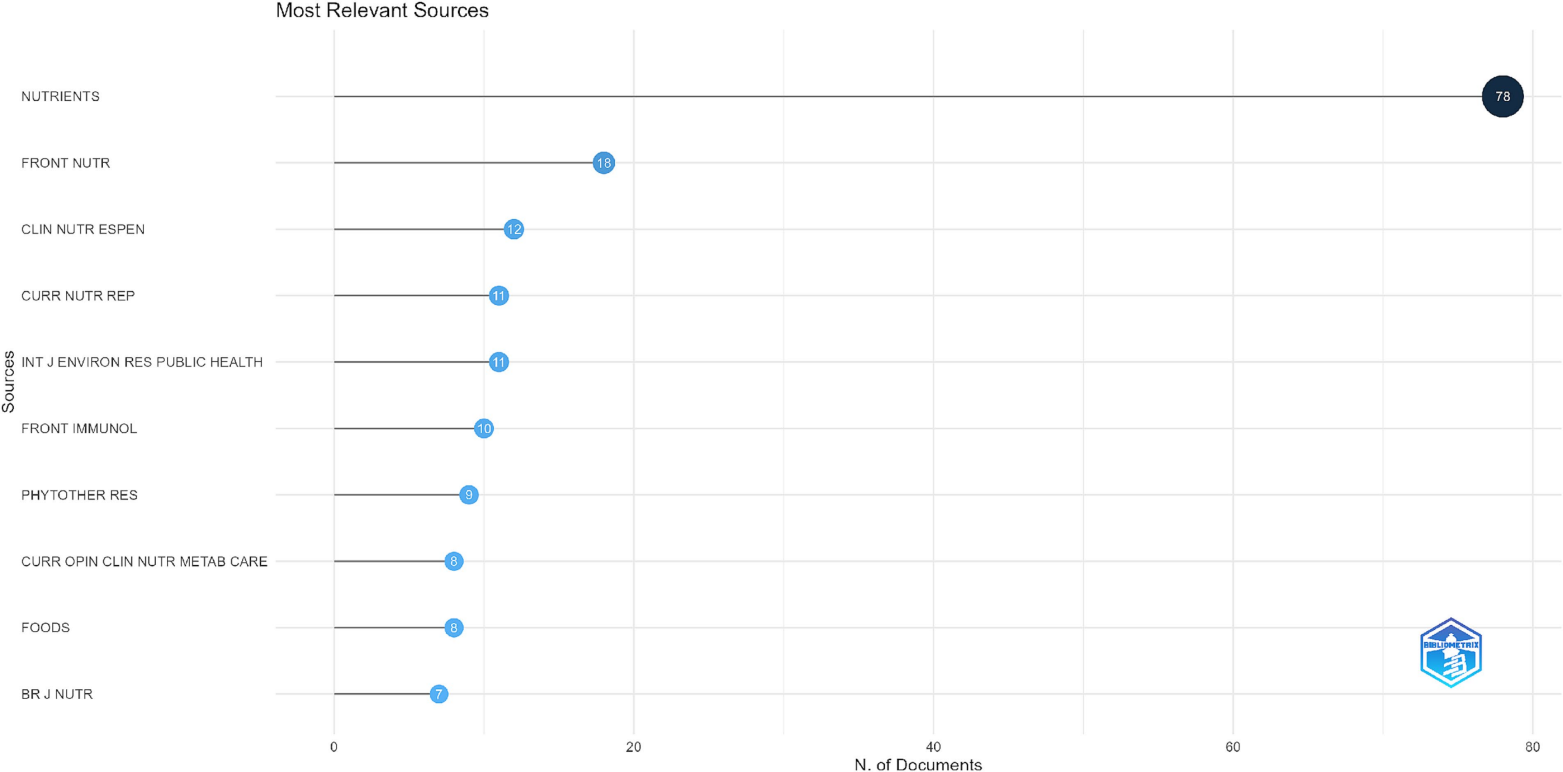
Top 10 most relevant journals by publication count.

In Figure 8, *“Most Relevant Sources,”* the top scientific journals per the number of published documents are shown. Moreover, the x-axis states the number of articles, while the y-axis ranks the journal. The journal *“Nutrients”* stands out as the most significant contributor, publishing 78 documents, far surpassing all other sources. *“Frontiers in Nutrition”* follows with 18 documents, while other notable contributors include *“Clinical Nutrition ESPEN”* (12 documents), *“Current Nutrition Reports”* (11 documents), and the *“International Journal of Environmental Research and Public Health”* (11 documents). Additional journals such as *“Frontiers in Immunology”* (10 documents), *“Phytotherapy Research”* (9 documents), and *“Current Opinion in Clinical Nutrition & Metabolic Care”* (8 documents) also make notable contributions. The list is rounded out by sources like *“Foods”* (8 documents) and the *“British Journal of Nutrition”* (7 documents), which, while smaller in count, still play a significant role in the field.

**Figure 9:**
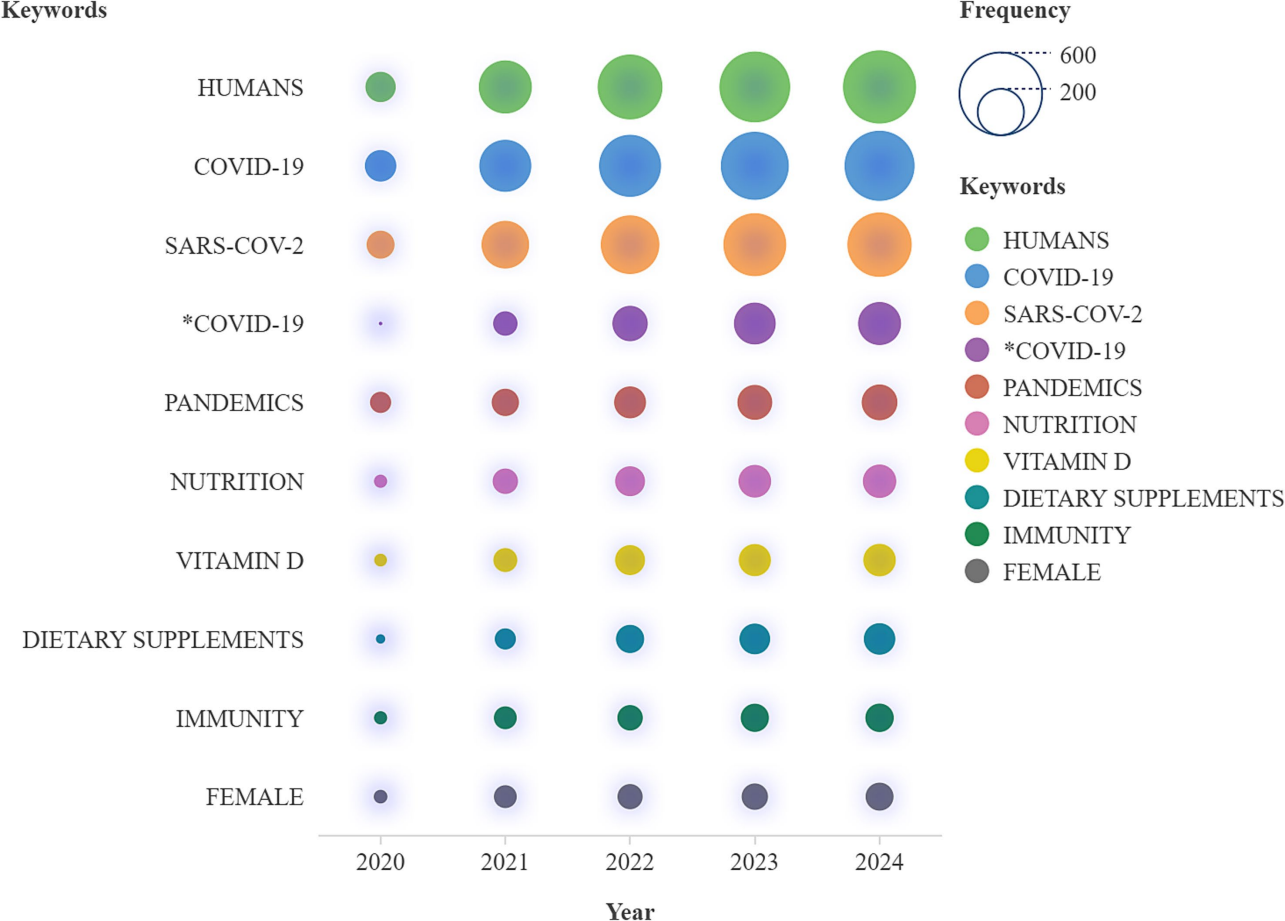
Words frequency over time.

Figure 9, “Words’ Frequency over Time,” displays the cumulative occurrences of key research terms between 2020 and 2024, offering a clear view of how research priorities have evolved. The x-axis represents the timeline, while the y-axis displays the total cumulative occurrences of the tracked terms. Each line corresponds to a specific keyword identified in the legend. The term “COVID-19″ emerges with the highest cumulative frequency by 2024, showing its essential role in research during these years. Related terms like “SARS-CoV-2,” “pandemics,” and variations of “COVID-19″ also show significant growth, further reflecting the pandemic’s widespread influence on scientific literature. Health-related keywords such as “nutrition,” “vitamin D,” and “dietary supplements” exhibit steady increases, highlighting the strong link between immune health and nutrition in the context of COVID-19. Other terms like “humans” and “immunity” consistently grow over time, reflecting their relevance across diverse studies. To conclude, nutrition and immunity terms are interlinked as a result of the research community to answer to the pandemic via the linkage of nutrition, immune support and public health.

**Figure 10:**
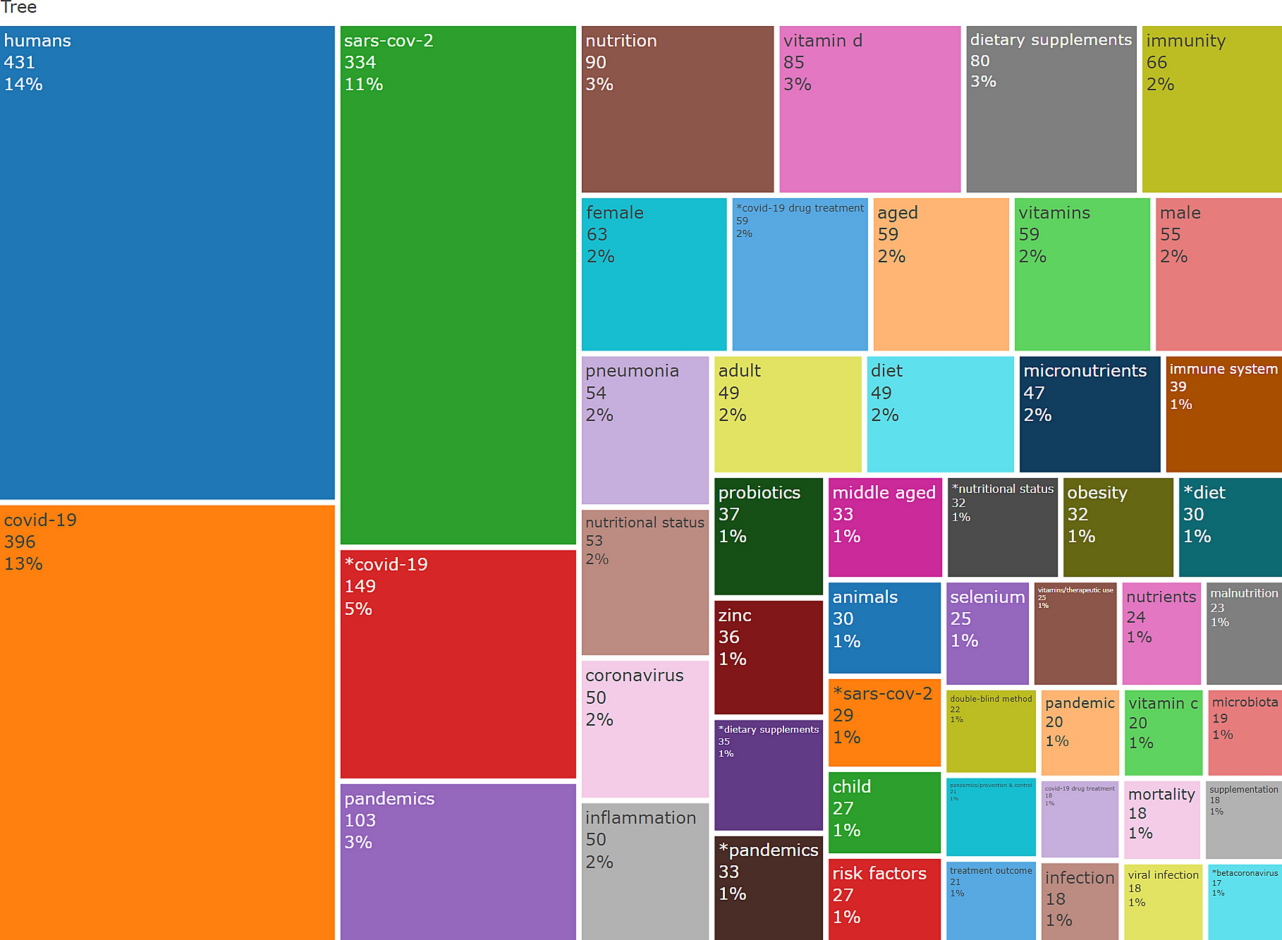
Treemap of most frequent keywords.

This tree-map presents a hierarchical representation of keyword frequency. This way, valuable insights can be used to understand keywords’ relative importance and frequency in a given dataset. Each rectangle represents a keyword, and the size reflects the term’s frequency—larger rectangles signify more frequent occurrences, while colours distinguish the terms visually. In Figure 10, each rectangle is annotated with the keyword, its number of occurrences, and the percentage concerning the total dataset (Figure 9).

Terms like “humans” (431 occurrences, 14%), “COVID-19” (396, 13%), and “SARS-CoV-2” (334, 11%) are nominated as core terms, emphasising the strong research focus on the pandemic and its impacts. Keywords related to nutrition and health, such as “nutrition” (90, 3%), “vitamin D” (85, 3%), and “dietary supplements” (80, 3%), highlight the connection between immune health and nutritional interventions between 2020 and 2024. In the same context, “immunity” (66,2%) and “immune system” (39, 1%) further pinpoint this relationship.

Specific demographic terms, such as “female” (63, 2%), “male” (55, 2%), and “aged” (59, 2%), reflect targeted research on population subgroups. Emerging topics like “probiotics” (33, 1%), “microbiota” (19, 1%), and “selenium” (25, 1%) suggest a growing interest in gut health, enteric microflora and micronutrient research. Furthermore, terms like “COVID-19 drug treatment” (35, 1%) and “pandemic prevention control” (33, 1%) highlight focused therapeutic and preventive research areas. Peripheral keywords, such as “malnutrition” (23, 1%) and “viral infection” (18, 1%), indicate less frequent but still relevant themes. The tree-map emphasises significant research topics, with “COVID-19,” “humans,” and “SARS-CoV-2” as foundational themes, as expected. Concurrently, it reveals the key role of nutrition, dietary supplements, and immune health in pandemic-related surveys and emerging research landscapes like probiotics and micronutrients.

**Figure 11:**
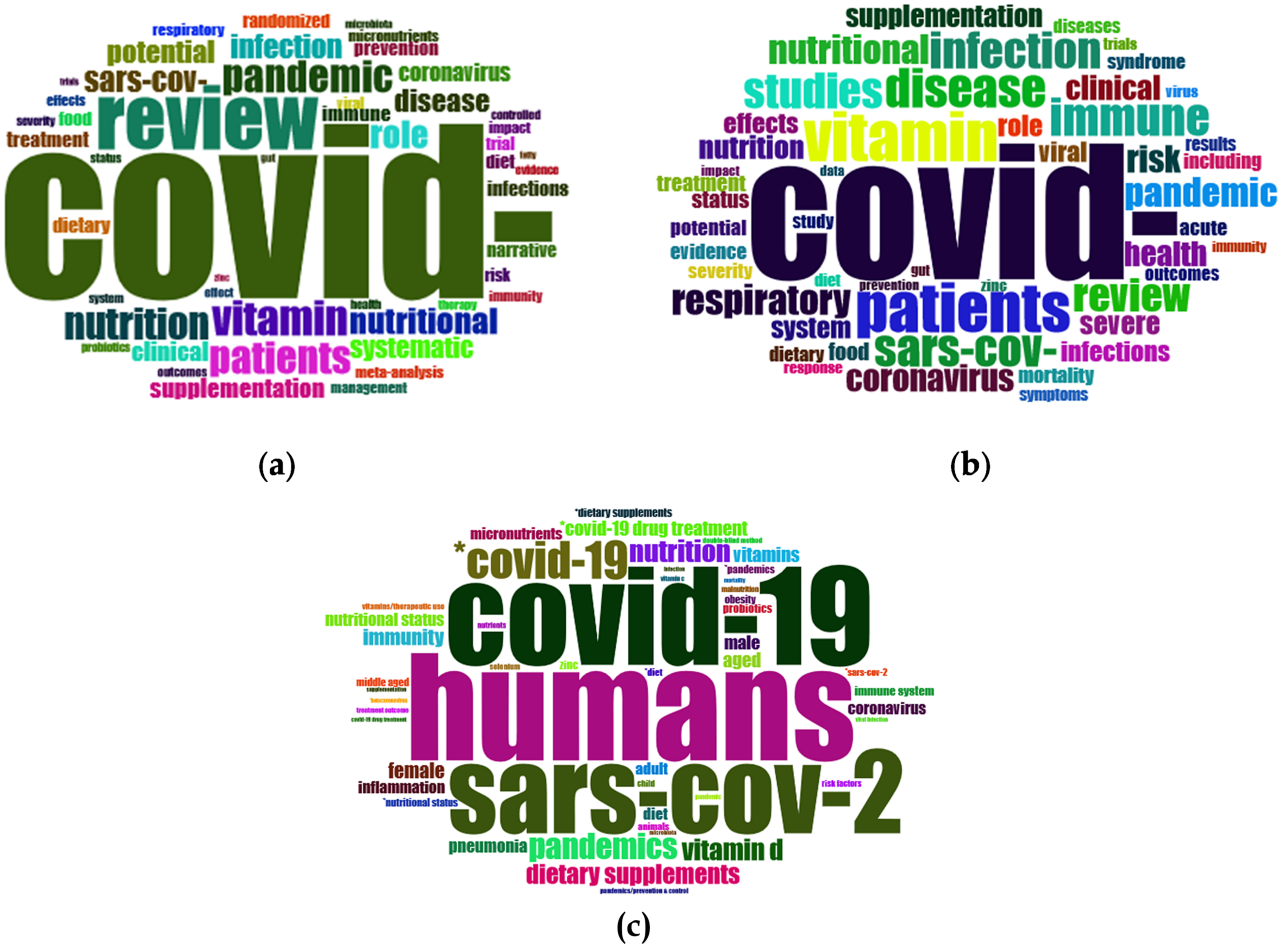
Frequency word clouds of top 50 words in **(a)** titles; **(b)** abstracts; **(c)** keywords plus.

The word cloud visualisations provide an overview of the central themes and research directions across the analysed dataset’s titles, abstracts, and Keywords Plus fields (Figure 11). In the titles, the dominant keyword is “COVID,” highlighting the pandemic as the primary research focus. Keywords like “review,” “pandemic,” and “patients” underline the significance of systematic reviews in the representation of research information to combat the impact of COVID-19. Moreover, keywords like “nutrition,” “vitamin,” and “nutritional” highlight the elevated research attempts to decipher if dietary factors play an essential role in the pandemic context. Clinical and methodological terms are also used by the researchers when referring to medical and epidemiological studies (“infection,” “SARS-CoV-2,” “prevention,” “systematic,” “meta-analysis,” and “randomised”). In the same line as the above, “COVID” remains the central term written in the abstract field. Frequently occurring terms like “patients,” “vitamin,” “disease,” and “pandemic” highlight themes related to COVID-19’s clinical impacts and nutritional considerations. Virological terms such as “SARS-CoV,” “coronavirus,” and “infection” reflect the focus on epidemiology and disease transmission.

In the Keywords Plus field, “COVID-19,” “humans,” and “SARS-CoV-2” are the most prominent terms, again emphasising the pandemic’s central role in the dataset. Related terms such as “pandemics,” “pneumonia,” and “coronavirus” highlight the virological and public health dimensions of the studies. Nutrition-related terms, including “nutrition,” “dietary supplements,” and “vitamin D,” reflect a strong focus on dietary interventions and their impact on health during the pandemic.

Immune response and inflammation, as well as demographic terms, are also utilised to emphasise medical and therapeutic protocols for specific target groups of the population, as shown in the above-mentioned word cloud (“immunity,” “inflammation,” “immune system,” “female,” “male,” “middle-aged,” “COVID-19 drug treatment,” and “therapeutic use”).

**Figure 12:**
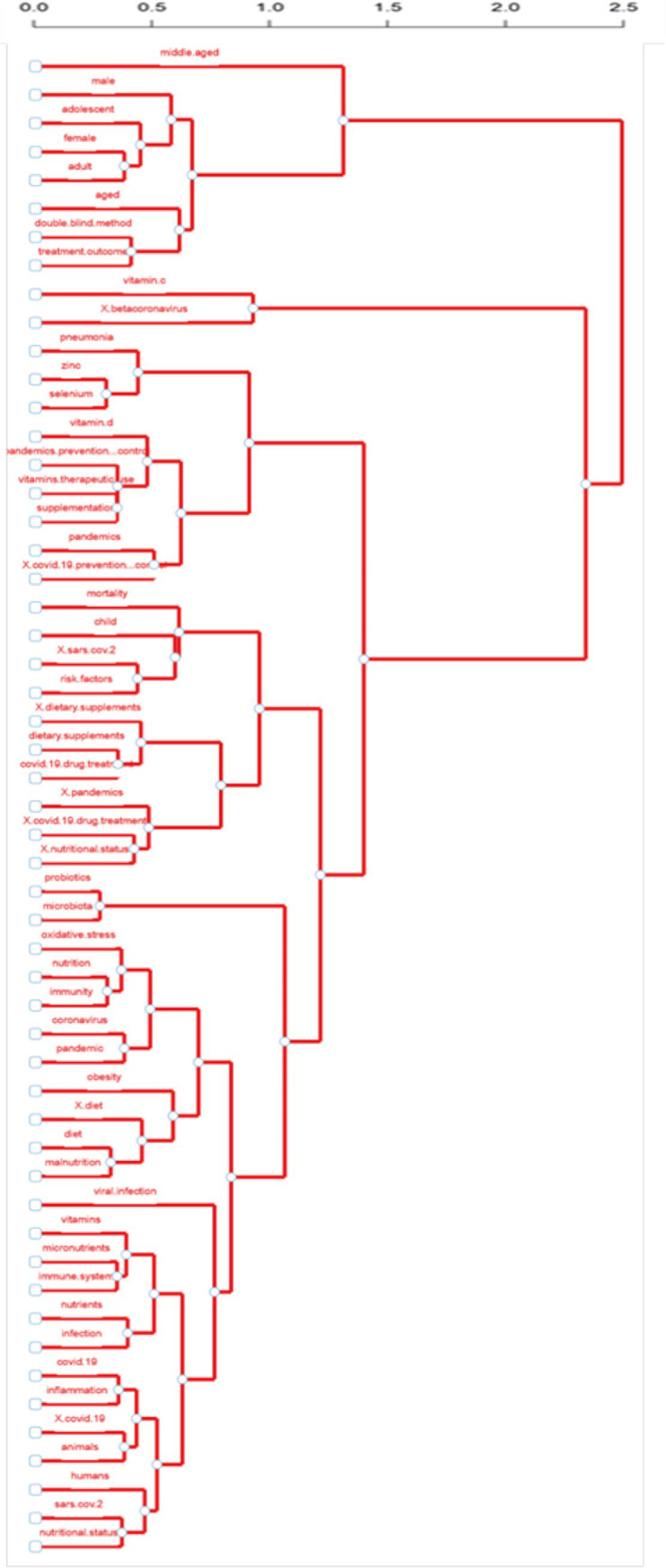
Factorial analysis of keywords.

Figure 12 shows the dendrogram of the frequency of occurrence of keywords based on factor analysis. In summary, the height of each branch represents the distances between keywords or groups of them. The distance of the keywords in the hierarchical structure also dictates their relevance. The closer the words are, the more semantically related they are and the more thematic context they are in, while the more significant the distance of their proximity, the smaller their thematic proximity, and the greater the height of the branch they belong to.

In this analysis, the dendrogram highlights clear keyword groupings that reveal significant concepts within the collection of keywords. Keywords related to population demographics, such as “male,” “female,” “adolescent,” “middle-aged,” and “aged,” form a distinct cluster, reflecting studies on specific population groups. Another cluster emphasises nutritional factors, grouping terms like “vitamin D,” “selenium,” “zinc,” and “nutritional status,” identifying a focus on nutrition and dietary supplementation. Strong associations are also acknowledged between disease-related keywords, including “COVID-19,” “SARS-CoV-2,” “pandemics,” and “pneumonia,” highlighting the emerging attention given to pandemic-related research. Subsequently, terms such as “immunity,” “inflammation,” “oxidative stress,” and “probiotics” are clustered together, focusing on the linkage of nutritional interventions and immune system operation and performance.

In conclusion, the research focuses on the pandemic is related to COVID-19, global health, and nutrition, and it stems from specific clusters of terms about nutritional supplements and the immune system during the pandemic crisis.

In the object density visualisation, each point, which in this image represents the authors, has a colour that indicates the density of objects at that point. The colours range from blue to green to yellow. The greater the number of objects in the area of a point and the greater the weight of the elements nearby, the closer the point’s colour is to yellow. Conversely, empty areas on the map or areas with a small number of objects in proximity and with a lower weight, the closer the colour of the point is to blue. Overall, the density visualisation highlights the critical role of key researchers while underscoring the need for a balanced and collaborative research ecosystem that promotes research, collaboration, and innovation.

**Figure 13:**
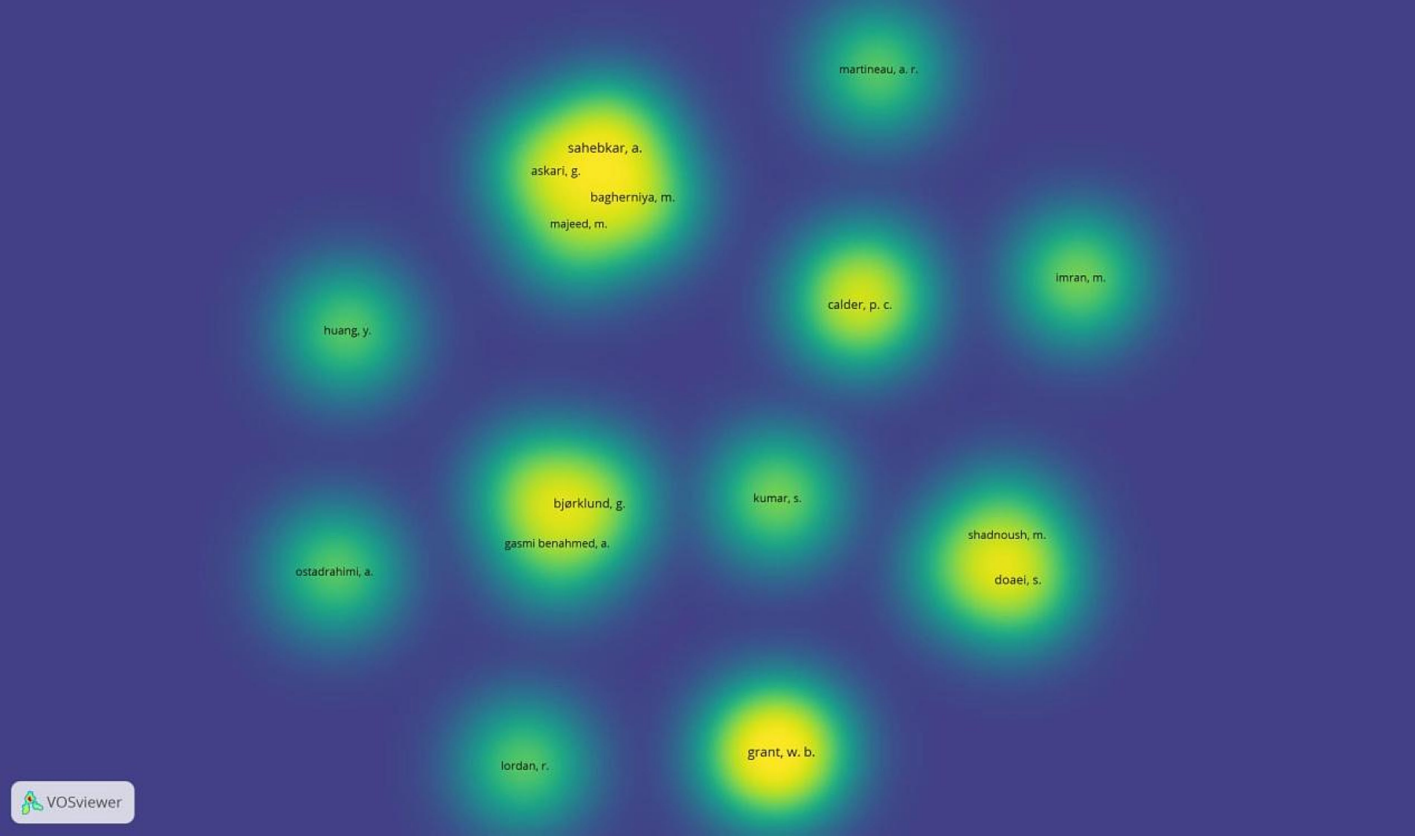
Author density visualisation.

Figure 13 presents the author density visualisation based on their research contribution and the dynamics of their collaboration. The research hotspots on the map depict areas where authorship and contributions are high, with prominent researchers with significant research output in the field, such as Sahebkar, A., Grant, W. B., Bagherniya, M., and Askari, G. (bright yellow). Other author groups with frequent research collaborations include Sahebkar, A., Askari, G., Bagherniya, M., Bjorklund, G., and Gasmi Benahmed, A., reflecting joint research output and collaboration. Author groups with Calder, P. C., Imran, M., and Martineau, A. R. appear as isolated clusters, focusing on specific research areas. Areas of moderate density include Doaei, S., Shadnoush, M., and Ostadrahimi, A., and indicate emerging researchers with increasing influence in specialised research areas.

**Figure 14:**
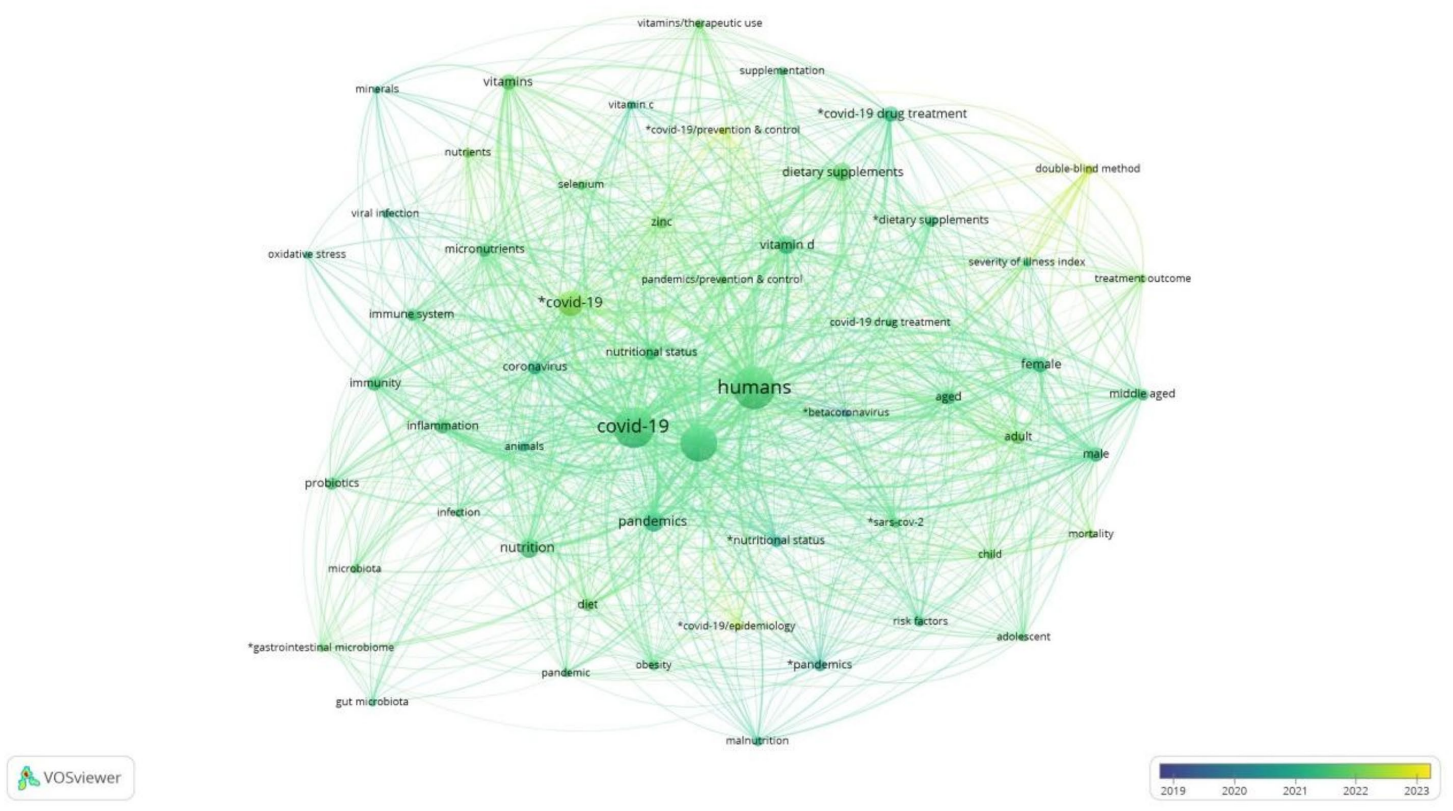
Chronological evolution of research themes on COVID-19 and nutrition, 2019–2023.

Figure 14 depicts the chronological evolution of research in the under-researched field of Covid and nutrition from 2019 to 2023. The size of each node is related to the frequency of occurrence of the terms, so larger nodes indicate a higher frequency of occurrence. In addition, the colour scale from dark to light blue represents the evolution of time, with darker shades indicating older years and lighter shades from green to yellow indicating more recent years and recent research pools.

Therefore, observing the picture, initially, the research focused on concepts and themes such as oxidative stress, inflammation, and the microbiome. At the same time, as the pandemic evolved, the research agenda was enriched, encountering terms such as nutritional supplements, immunity, and vitamin D. Also, concepts that summarise fundamental research issues, such as nutrition, humans, and COVID and their synonyms, are consistently detected throughout this period. In addition, vitamins such as B and D, micronutrients, probiotics, zinc, minerals, and selenium have been in research efforts for their therapeutic use since the middle of the pandemic. Finally, the visualisation of the chronological evolution of research shows the research priorities at the beginning and end of the pandemic, moving from basic research on health and the immune system response to more specialised topics targeting answers from nutritional and clinical research.

**Figure 15:**
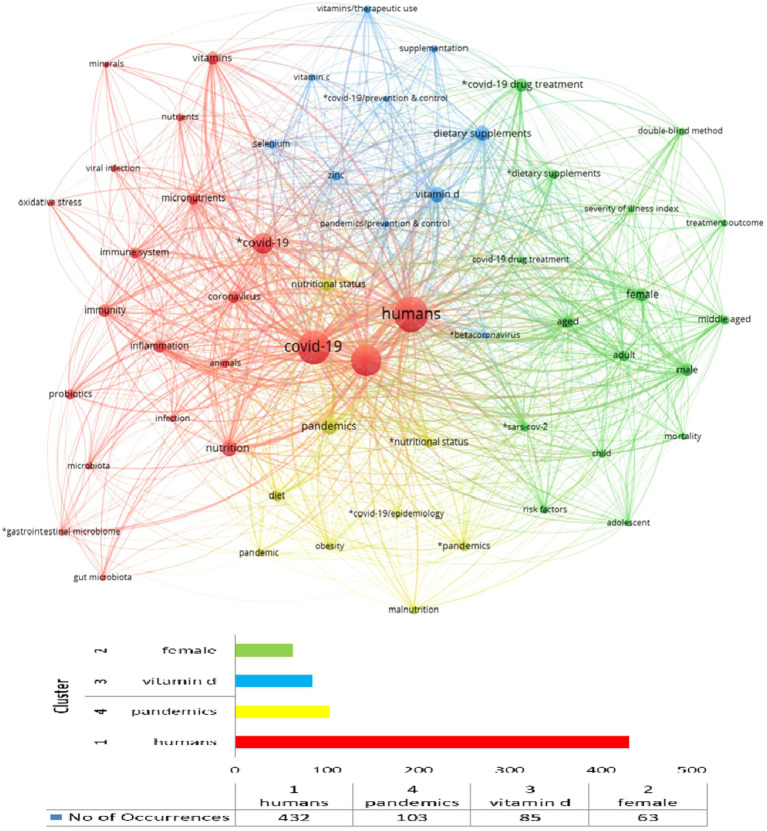
Keyword analysis and the number of occurrences of the keywords in each cluster.

Figure 15 depicts the visualisation co-occurrence network of the keywords collected, mapping their linkages and relevance within the dataset. The following network visualisation was derived from 2,372 keywords, setting a minimum appearance limit of 15, resulting in a bibliometric map consisting of 56 keywords. In the outline, the network is divided into four clusters, shown in Figure 14, based on their colour. Each element in the four colour groups is unique, namely each keyword, and cannot be included in another group. The lines connecting nodes represent co-occurrences of keywords. The thicker the line, the more frequently the terms appear together in the dataset. Finally, the closer the two elements are together, the greater their relatedness. Figure 14 also shows the keywords with the highest frequency of appearance per cluster.

Based on the network visualisation, the keywords with the highest frequency of occurrence per cluster are “humans” in the red group, “pandemics” in the yellow group, “vitamin D” in the blue group, and “female” in the green group. The number of occurrences for each keyword is 432, 103, 85, and 63, respectively. The analytical distribution of all keywords in each cluster is presented in Table 1.

**Table 1:** Thematic keyword clusters in COVID-19 and nutrition research (2020–2024), based on co-occurrence analysis (*n* = 56 keywords, threshold ≥15).

Cluster identification	Keywords
Red	*COVID-19, *gastrointestinal microbiome, animals, coronavirus, COVID-19, gut microbiota, humans, immune system, immunity, infection, inflammation, microbiota, micronutrients, minerals, nutrients, nutrition, oxidative stress, probiotics, SARS-CoV-2, viral infection, vitamins
Green	*COVID-19 drug treatment, *dietary supplements, *SARS-CoV-2, adolescent, adult, aged, child, COVID-19 drug treatment, double-blind method, female, male, middle-aged, mortality, risk factors, severity of illness index, treatment outcome
Blue	*beta-coronavirus, *COVID-19/prevention & control, dietary supplements, pandemics/prevention & control, selenium, supplementation, vitamin c, vitamin d, vitamins/therapeutic use, zinc
Yellow	*COVID-19/epidemiology, *nutritional status, *pandemics, diet, malnutrition, nutritional status, obesity, pandemic, pandemics

The red group, which is the most numerous, consists of 21 terms, the yellow group of 16, the blue group contains 10 terms, while the group that gathers the fewest terms is the yellow one with a total of 9 keywords.

The red cluster centred on the role of nutrition, nutrients and immunity. Keywords such as immune system, immunity, infection, and inflammation, which were collected in this cluster, indicate the high contribution of nutrition in supporting the immune system, managing oxidative stress and mitigating the effects of inflammation. In addition, the same cluster also emphasises the auxiliary role of vitamins and minerals as well as the impact of probiotics on gut health and the management of viral infections and diseases, as can be deduced from the other keywords oriented from the red cluster. Moreover, the anti-inflammatory effects of nutritional interventions in viral infections, e.g., COVID-19, are being investigated within the general framework defined by the red cluster regarding the importance of nutrition as a preventive and adjuvant strategy for managing infections, in combination with the role of probiotics in enhancing the resilience of the immune system.

The role of public health and epidemiology is highlighted in the green cluster, particularly emphasising the interaction and interplay of demographic factors, risk assessment and nutritional supplements. Research value is also given to specific characteristics of the population, such as age group, gender, and mortality, to decipher their role in understanding the evolution of the disease and the pandemic (“adolescent, adult, aged, child, female, male, middle-aged, mortality”). In addition, research initiatives on the effects of the COVID-19 disease through the contribution of nutritional supplements in several demographic groups and the results of research on the mitigation of the risks of the disease and their impact on different population groups aim to develop health policies to address pandemic crises.

The blue cluster reflects more targeted approaches to clinical research on the role of nutrition, control and prevention of the disease COVID-19. Keywords that emphasise this direction (“*covid-19/prevention & control, pandemics/prevention & control”) are combined with the therapeutic role of micronutrients and dietary supplements to improve health and reduce the severity of the disease (“dietary supplements, supplementation, vitamins/therapeutic use”). In particular, in this group, the critical role of vitamins, such as vitamins C and D, selenium and zinc, is emphasised in the strategic research on the nutritional value in preventing and strengthening the immune system during a disease such as COVID-19. Finally, the blue cluster highlighted the vital role of various micronutrients in maintaining nutritional health and the research that should be included in controlling this and a future pandemic.

The yellow cluster includes keywords such as COVID-19/epidemiology, nutritional status, pandemics, diet, malnutrition, and obesity that illustrate the research interest in nutrition and epidemiology, targeting specific population characteristics. A central theme of this cluster refers to the nutritional status of a portion of the population, namely malnutrition on the one hand and obesity on the other, as characteristics that may increase the vulnerability of these individuals to serious infections such as COVID-19. Thus, the nutritional behaviour and status of individuals seriously impact public health in the context of a pandemic crisis since these extreme conditions, malnutrition and obesity, act as co-morbidities burdening the overall health of individuals. Subsequently, identifying these terms in the bibliographic network in a single cluster further underlines the need to integrate nutritional intervention plans for a balanced diet into future health policies. Finally, malnutrition and obesity also conceal significant inequalities in populations, highlighting the need to strengthen food security and the regulation of food supply chains and their declaration in a pandemic situation on the one hand and the proper diet of the population and specific subgroups on the other.

## 4 Discussion

### 4.1 COVID-19 and nutrition

**Figure 16:**
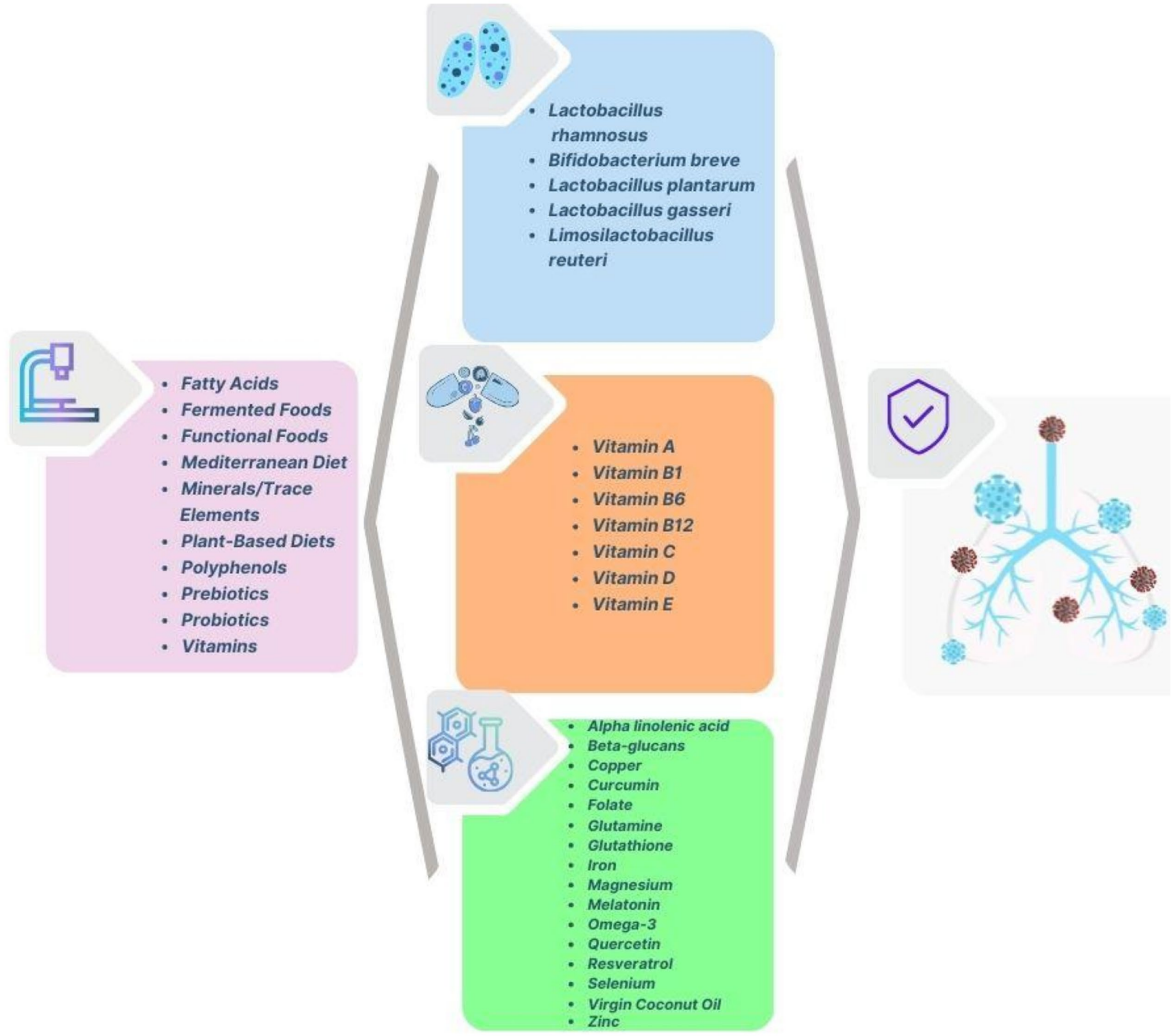
Nutritional interventions relevant to COVID-19 prevention and management.

The analysis of the clusters shows a comprehensive research focus that brings together public health, nutrition, epidemiology, and clinical interventions in response to COVID-19. The red cluster emphasises the importance of nutrition and immunity, particularly highlighting micronutrients, dietary supplements, and inflammation management. The green cluster centres on public health and epidemiological studies, examining risk factors, demographics, and disease outcomes. The blue cluster points out the therapeutic benefits of micronutrients such as vitamin C, D, selenium and zinc in enhancing immunity and aiding clinical recovery. The yellow cluster connects nutrition and public health, stressing the impact of malnutrition, obesity, and dietary strategies in managing pandemics. Overall, the bibliographic network and the respective terms and clusters correspond to an interdisciplinary research approach that highlights research studies and practical interventions to strengthen public health resilience and preparedness for future pandemics. Figure 16 illustrates a comprehensive overview of the nutritional interventions that enhance immune function and respiratory health, targeting prevention, symptoms and treatment of COVID-19.

In addition to the current research activity captured by the PubMed database, several studies are ongoing on the dynamic relationship between diet, probiotics and the symptomatology and progression of COVID-19, as shown by the studies available in the PROSPERO database up to the 24th of December 2024. Overall, systematic reviews focus on the role of probiotics and dietary supplements in managing disease symptoms, enhancing immunity and reducing the severity of respiratory infections, among others. Nutritional strategies and lifestyle policies are also mentioned, as well as specific dietary interventions, such as vitamin D and C supplements, the effect of probiotics on the gut microbiota and the relationship between diet and long-term outcomes of COVID-19.

Finally, and in combination with the results of this study, as shown in the keywords in the bibliographic networks, specific demographics, including older adult populations, children and patients with comorbidities, are a target for investigation by evaluating their results. Literature reviews have also pinpointed the role of preventive strategies for the disease evolution and symptoms through nutritional supplements and probiotics in enhancing immunity and managing health risks associated with COVID-19, emphasising the interdisciplinary approach to the pandemic crisis. The list of all nutrition, probiotics, and COVID-19 studies with Review Status: “Ongoing” retrieved from the PROSPERO database on 24/12/2024 is in the Supplementary Table 2.

The green cluster bridges clinical research for treating COVID-19 and nutritional science, depicting a holistic approach to mitigating the pandemic. In addition, the green cluster emphasises the variability of research in different population subgroups, as reflected by the keywords included, highlighting the role of age, gender and other risk factors in the effectiveness of treatment and disease progression. This also substantiates the presentation of the keywords, double-blind method, and treatment outcome, which identify the research application of rigorously designed research protocols. Lastly, studies that evaluate mortality, disease severity and treatment outcomes provide reliable information for clinical decision-making and policy formulation across the spectrum of citizens’ social and economic lives.

Since the beginning of the pandemic, various pharmacological and therapeutic methods have been proposed to combat it, mainly antiviral substances used in previous respiratory infections, such as SARS and MERS. Furthermore, since the initial phase of the COVID-19 response, many countries have proposed corresponding plans to produce vaccines (36–38). In addition, nutrition through the intake of micronutrient components of bioactive substances from natural products has been proposed as a primary adjuvant for the duration of symptoms, the reduction of mortality and morbidity, and nutritional supplements for treating COVID-19 (39, 40).

Several studies have also focused on various demographic factors. During COVID-19, research on immunological and metabolic changes in the human body has focused on the entire age range, from children, adolescents, and young people to older people of both sexes.

Research questions have been raised related to dietary supplements and, more generally, to dietary patterns and habits and their effects during the pandemic. In addition, lifestyle factors, such as diet and physical activity, are compromised in old age and are essential factors in COVID-19 infection (38, 41). Age over 50 years was the most relevant risk factor for developing a post-acute COVID-19 condition (PCC), followed by female gender. The most important protective factor against PCC was vaccination against SARS-CoV-2 (42).

As shown by the results of the bibliometric analysis, terms related to COVID-19 therapy and nutritional supplements appear in the latter stages of the timeline (Figure 14), reflecting their emergence as research priorities as the pandemic matured. The shift from public health measures (distancing, masks, travel restrictions, etc.) to targeted therapies is also visible. Combining the terms identified in the green group, such as “treatment outcome,” “risk factors,” and “nutritional supplements” with the word clouds confirms their thematic importance. Furthermore, the ongoing emergence of “disease severity” and “drug therapy for COVID-19” keywords underlines their central role in clinical research, as evident from the number of documents retrieved in the PubMed database, 57 in clinical and randomised controlled trials. The current bibliometric study shows an increasing focus on pharmacological interventions and integrated therapies. The emphasis on pharmaceutical therapies with nutritional supplements, including demographic terms, suggests a growing interest in the stratified analysis aligned with the green cluster keywords.

Plant-based dietary patterns, in addition to antioxidants and vitamin supplements, have been examined by the research community amid the pandemic crises (43, 44). Promising results were identified, particularly in reducing the severity and mortality. Moreover, physical activity has been linked to reduced hospitalisation and death risk, which underlines the protective role of a healthy lifestyle (45). In this vein, it is notable that sarcopenia has also been recognised as an essential risk factor for mortality in critically ill patients, highlighting the importance of maintaining muscle health (46).

Combining these keywords in the cluster, it is noteworthy that the main risk factors for mortality include advanced age, male gender, and pre-existing conditions such as cardiovascular disease, diabetes, cancer, and severe kidney disease, which are often exacerbated by elevated pro-inflammatory cytokines (47). Dietary supplements and immune-boosting strategies can mitigate hyperinflammation and support recovery in high-risk individuals (48).

Overall, findings from the green cluster indicate that developing personalised treatment protocols that consider patient demographics and risk profiles may be a valuable direction. In addition, nutritional supplements as adjunctive therapies may offer cost-effective options for managing disease severity. In parallel, studies with multiple demographic characteristics can guide vaccination strategies, resource allocation, and public health campaigns targeting high-risk groups.

It is important to note that the effects of probiotics on COVID-19 severity and progression cannot be interpreted in isolation. Host-specific factors likely influence their efficacy, including age, sex, comorbidities (such as diabetes, obesity, or cardiovascular disease), microbiome composition, and vaccination status. In addition, nutritional status and the usage of dietary supplements—particularly vitamin D—are essential confounders that may modulate immune responses and impact outcomes. While several of the identified studies in this review included such variables in their designs or subgroup analyses, the degree to which these factors were systematically accounted for varies. As such, any findings regarding probiotic benefits should be viewed within the view of these interacting components, highlighting the need for more personalised research in future efforts and protocols.

The blue group emphasises the role of micronutrients in preventing and treating COVID-19. The role of vitamins, led by vitamin D, and trace elements such as selenium and zinc have been the focus of the literature during the pandemic. The inclusion of selenium and zinc in this group is linked to selenium’s role in regulating oxidative stress, which was also identified in the red group, and zinc’s role in supporting the immune system. These findings are consistent with specific pandemic keywords identified in the timeline visualisation, where terms such as “COVID-19 prevention” and “nutritional status” are included. Additionally, keywords that emerged from this cluster (zinc, selenium, and vitamin D) gained prominence in the later stages of the timeline, reflecting the increasing focus on these specific micronutrients as research matured beyond the early pandemic response (Figure 14).

Bibliographic indicators such as manuscript type demonstrated that systematic reviews and meta-analyses focusing on vitamin D, zinc, and other supplements were particularly prominent, reflecting the firm reliance on high-quality evidence. This also supports the observed dominance of review articles in the dataset, where supplementation emerged as an important preventive and therapeutic topic across the literature collected from the PubMed database.

One of the elements that has emerged for its potential as a nutritional and therapeutic agent in the COVID-19 pandemic is selenium. Selenium is characterised by its antioxidant and anti-inflammatory properties, which are associated with improved outcomes in viral diseases, including COVID-19, against oxidative stress and hyperinflammation, which are the main characteristics of severe COVID-19 cases (49–51). Studies have consistently reported lower serum selenium levels in patients with COVID-19 compared to healthy individuals. Patients with reduced selenium levels have been linked with high rates of morbidity and mortality, which underlined the role of selenium as a protective avenue in parallel with other therapeutic measures against COVID-19 (52–54).

Additionally, selenium’s ability to reduce inflammatory cytokines, such as IL-6, positions selenium as a key regulator in preventing cytokine storms—a significant cause of severe complications in COVID-19. Reviews have highlighted selenium’s ability to inhibit SARS-CoV-2 proteases and reduce ACE2 receptor expression, key events during viral replication and entry (49, 55). Additional research has highlighted selenium’s synergistic action with other micronutrients, such as zinc and vitamin D, to enhance the antiviral shield (56, 57). Dietary interventions with nutritional supplements applying selenium with these micronutrients have been proposed to support innate and adaptive immunity and mitigate inflammation and symptoms (58). Selenium supplementation at early stages of infection in high-risk populations with specific demographic characteristics, such as the older adult or those with comorbidities, has been suggested as an adjunct to standard therapeutic care for patients (59).

Likewise, selenium and zinc also play a supportive role in alleviating the symptoms of COVID-19. Zinc is involved in several cellular events, including activating immune cells, regulating cytokines, and maintaining barrier function by epithelial cells (60, 61). Other than antiviral activity, downregulation in the expression of ACE2 receptors has also been mentioned, further restricting the virus’s entry into host cells. Thus, partial deficiency of this element can enhance oxidative stress and the risk of hyperinflammation (62, 63).

Low serum zinc levels and supplementation have reduced patient mortality and survival (64). Zinc’s mode of action and immunotropic profile focus, among other things, on dampening cytokine storms, inhibiting viral replication and enhancing host immune responses (65). For example, study data show that mortality was reduced by 37% for patients who received zinc compared to those who did not. Although zinc supplementation thus improved survival rates, it did not affect the severity of symptoms, and therefore, the main benefits of zinc supplementation in COVID-19 infection lie in mitigating disease progression (66, 67). This micronutrient also regulates oxidative stress and inflammatory pathways, essential in severe COVID-19. Some studies have identified its deficiency as a risk factor, mainly in older adult patients and patients suffering from chronic diseases (66, 68). Zinc modulates gut barrier integrity, thus limiting systemic inflammation and improving mucosal immunity. The bidirectional relationship between zinc and gut health is an added avenue to exploit its benefits in COVID-19 management (69).

Thus, zinc is clinically valuable in nutritional intervention against COVID-19. Overall, further research and clinical trials to document its use in standard medical protocols for COVID-19 treatment will be necessary for effectiveness in the presence of other vitamins and minerals.

Before the era of vaccines against COVID-19, the global research community sought therapeutic solutions through nutritional supplements, mainly substances such as vitamins that had a safe profile for their administration. Vitamin C was also investigated in this direction because it was also studied for other respiratory diseases (70).

Vitamin C is known for its therapeutic effects, although for the general population, a high dose of vitamin C does not affect the course of the common cold. The data on pneumonia are also unclear, and the research community had not expressed certainty about the treatment of COVID-19 in 2020 (71, 72). In another study of the same year, the intravenous administration of vitamin C is proposed based on the fact that the symptoms of the common cold are milder after a dose-dependent administration of vitamin C. Specifically, intravenous infusion of vitamin C in the range of 6–24 g/day, to enhance interferon production and support the anti-inflammatory effect of glucocorticosteroids, especially for patients with severe COVID-19 has been proposed. Two studies that focused on Vitamin C due to its anti-inflammatory, immunomodulatory, antioxidant, and antiviral properties, also characterised its possible use without documenting clear recommendations against COVID-19 were published in 2022 (73, 74).

In a 2021 study that reviewed all clinical studies on the application of vitamin C in patients with COVID-19, 12 studies in total, it concluded that the role of vitamin C with intravenous administration could improve oxygenation parameters, reduce inflammation markers, days of hospitalisation and mortality, especially in severe and complicated cases (75, 76). The same study also confirmed that oral administration can accelerate the rate of recovery in mild cases. The combination of vitamin C and zinc was ineffective in studies that studied their combined effect at a preliminary level; the researchers concluded that more studies would shed light on this prospect (77).

Another vitamin, vitamin D, has been at the forefront of studies of nutritional interventions against COVID-19. Specifically, of the 599 articles collected in this study, just over 60 manuscripts devoted their content to its mode of action, clinical studies, role and therapeutic potential.

The mechanism of action of vitamin D lies in the reduction of pro-inflammatory levels such as interleukin-6 (IL-6) and the increase of anti-inflammatory cytokines to stop the cytokine storm (78). In addition, this vitamin is also involved in the renin-angiotensin-aldosterone system (RAAS) through its interaction with angiotensin-converting enzyme 2 (ACE2), which aims to prevent the entry of the virus into the host cells. Thus, it contributes to treating respiratory infections, including those caused by COVID-19. However, the complete deciphering of the mechanisms against COVID-19 has not been completed, so it is emphasised that any benefit of vitamin D in the treatment of COVID-19 should be documented with rigorous clinical trials. These trials should examine dosage, stage of the disease, its intensity, population groups, such as the older adult, and comorbidities (79–83).

For example, a daily intake of 400–1,000 IU is considered a safe dosage effective for maintaining vitamin D levels in the general population. It is also important to emphasise that the beneficial effects of vitamin D administration on days of hospitalisation, for example, and the reduced mortality were not confirmed and statistically significant, thus remaining the results at the level of trends and noteworthy research hypotheses that need further investigation (81, 82, 84).

Studies have also been identified that highlight the role of vitamin D at a preventive level since its adequacy strengthens the body’s defences, reducing the viral load, in the older adult and patients with diabetes and obesity, indicating the aggravating role of the presence of comorbidity in patients with Covid-19 (85). It is no coincidence that countries associated with high mortality and severity of COVID-19 patients also have high rates of vitamin D deficiency. Vitamin D has also been studied in the context of general health programs and prevention strategies in the general population, requiring more specialised research and nutritional and therapeutic intervention protocols (85, 86).

Regarding morbidities and risk factors, vitamin D deficiency is characteristic, as it also contributes to the vulnerability of older adult individuals, individuals with chronic diseases, and overweight individuals (87, 88). Regarding the epidemiology of COVID-19, Vitamin D deficiency is also considered in individuals with diabetes and hypertension, with a risk of severe infection and increased mortality rates. Therefore, the prevalence of vitamin deficiency should also be further investigated clinical findings regarding demographic characteristics and COVID-19 disease (88, 89).

The European Society of Clinical Nutrition and Metabolism (ESPEN) has published practical recommendations and concise guidelines for managing COVID-19 patients regarding nutritional and dietary regimens since 2020 (90). These guidelines emphasise, inter alia, the need for individualised support, especially for people with malnutrition, and adequate prevention of trace elements and vitamins (vitamins A, D, B6, B12, zinc, and selenium) to strengthen the patient’s immune system.

In the yellow group, in addition to terms related to our search, we find two important risk factors: obesity and malnutrition. These are two multifaceted issues related to public health and the coronavirus. The coexistence of obesity and malnutrition in a single cluster, namely the yellow one, spotlights a paradox due to social, economic, and nutritional disparities. The above dietary challenges, malnutrition and obesity, are often highlighted as risk factors and gained additional attention during the pandemic period.

Obesity as a risk factor contributes to increased levels of disease severity, complications in the body and mortality during the pandemic crisis. The clinical picture of obesity appears as low-level and chronic inflammation with metabolic dysfunction. This burdens the body’s immune function, increasing the likelihood of the onset of the cytokine storm observed in patients with COVID-19 (91, 92). The review of 44 studies from 18 countries around the world revealed that obesity was associated with a higher risk of complications, increased risk of ICU admission, and adverse effects on intubation and mortality. It also found that patients’ vulnerability was associated with higher body mass index (42, 93).

Comorbidities also play an essential role in the development of the disease, with obesity being one of those that have been investigated for their role in the disease from COVID-19, along with diabetes and cardiovascular diseases (91, 94). Another factor that changed dietary patterns was reduced physical activity, which directly affected body weight during the pandemic and the consumption of unhealthy foods (95). The above components adversely affected the obesity rate, emphasising the critical role of metabolic health during the COVID-19 crisis (96). Nutritional interventions to reduce fatty acids and promote a healthy diet to combat obesity emerged during the coronavirus pandemic as an independent public health problem for the population and a critical risk factor that burdens patients with COVID-19 (97, 98). The pandemic crisis also served as a global alarm on obesity, highlighting it as a problem with profound implications for the entire public health system (94, 99).

Malnutrition is another parameter that adversely affects especially vulnerable groups with COVID-19. The clinical picture of malnutrition is described as a condition with partial deficiencies in micro-and macronutrients, resulting in immune system dysfunction, impairing the valid prognosis of the disease, and increasing susceptibility to infections (100). As mentioned in other keywords from the previous clusters of the bibliographic network, a sufficient and abundant supply of vitamins and minerals helps regulate the overall immune response (101).

Malnutrition, in combination with muscle loss and obesity, has been associated with an increased risk of morbidity and mortality, expressed by prominent systemic inflammation and longer disease duration in critically ill patients (102). Continuous nutritional monitoring should be performed systematically at admission, during hospitalisation, and after the recovery of patients (103).

Malnutrition worsened during the pandemic due to disrupted food supply chains, trade and distribution, and limited access to healthcare facilities (104). Micronutrient supplements for patients with COVID-19 and personalised nutritional treatment plans were proposed to improve the clinical picture of patients and address malnutrition. However, with obesity and malnutrition, a multifactorial approach is required, including educational and dietary public health policies and nutritional patterns with sufficient nutrients (104, 105).

### 4.2 Probiotics

Probiotics have been defined as “live microorganisms that, when administered in adequate amounts, confer a health benefit on the host.” Many commercial probiotics are specific strains of Lactobacillus sp. and Bifidobacterium sp. (106). The role of probiotics, sometimes more intensely and sometimes less emphatically throughout the pandemic, was explored based on the scientific constant that microbial flora plays a vital role in the patient’s immune competence. Consequently, the scientific community initially suspected the auxiliary role of probiotics and tested it mainly in the initial phase of the pandemic (Table 2).

**Table 2:** Summary of selected studies on probiotics in the management of COVID-19 and other respiratory infections.

Supplement	Main findings/Outcome	Reference
Combination of *Lactobacillus plantarum CECT7481, L. plantarum CECT 7484, L. plantarum CECT 7485, and Pediococcus acidilactici CECT 7483 vs. Placebo*	Severity progression of COVID-19, stay at ICU, mortality ratio	(162)
Probiotics	Several RCTs reports fewer new episodes, decreased duration of episodes and less severe symptoms	(163)
*Bifidobacterium longum BB536*	Decreased the duration of cough, sore throat, fever and runny Nose	(164)
Dietary Supplement: Probiotic vs. No Intervention	Cases with discharge to ICU.	(165)
Probiotic (Lactobacillus) vs. Control (Maltodextrin)	Occurrence of SARS-CoV-2 infection in healthcare workers.	(166)
*Lactobacillus rhamnosus, Lactobacillus plantarum, and Bifidobacterium longum*	A Cochrane meta-analysis review of 8 different trials reported the beneficial role of probiotic strains in reducing the risk of ventilator-associated pneumonia	(167)
*Bifidobacterium* spp.*, Lactobacillus* spp. *and/or Streptococcus spp*	Decreased the risk of developing a respiratory tract infection, mainly in adults	(168)
*S. thermophilus DSM 32345 L. acidophilus DSM 32241 L. helveticus DSM 32242 L. paracasei DSM 32243 L. plantarum DSM 32244 L. brevis DSM 27961 B. lactis DSM 32246 B. lactis DSM 32247*	SG: remission of diarrhoea and other symptoms 72 h after oral bacteriotherapy. Estimated risk of developing respiratory failure: Eight-fold lower in SG. CG: Higher ICU admission and mortality rates.	(169)
*Probiotic bacteria Lactobacillus gasseri PA 16/8, Bifidobacterium longum SP 07/3, Bifidobacterium bifidum MF 20/5*	Intake of probiotics had no effect on the incidence of common cold infections, but significantly shortened the duration of episodes by almost 2 days and reduced the severity of symptoms. Immunological: IG had a larger increase in cytotoxic T plus T suppressor cell counts and in T helper cell counts.	(170)
*Lactobacillus gasseri SBT2055*	Reduced weight loss, lower viral load in the lungs of infected mice, along with the reduced expression of proinflammatory cytokines	(171)
*Probiotic*	The computational representation depiction and molecular dynamics study demonstrated that postbiotics, plantaricin compounds, resulting from the metabolism of *Lacticaseibacillus plantarum,* showed antiviral activity • They block the viral entry by binding with RNA-dependent-RNA polymerase (RdRp) enzymes, SARS-CoV-2 receptor-binding domain (RBD) and angiotensin-converting enzyme 2 (ACE2)	(172)
Older adult people vaccinated with mRNA-based vaccine against SARS-CoV-2 (n = 98)	*L. coryniformis K8*: enhances vaccine-specific immune responses against SARS-CoV-2 in older adult populations.	(173)
*Lactobacillus casei Shirota*	Decreased pneumonia and increased recovery of pulmonary Function	(174)
Strains *Lactiplantibacillus plantarum* KABP022, KABP023, KAPB033, *Pediococcus acidilactici* KABP021 (totaling 2 × 109 CFU)	Complete symptomatic remission and viral clearance at day 30 higher in IG [RR: 1.89 (95% CI 1.40–2.55); *p* < 0.001]	(175)
*L. plantarum KABP022 L. plantarum KABP023 L. plantarum KABP033 P. acidilactici KABP021*	Complete remission in 53.1% of patients in SG and 28.1% in CG. Reduced viral load, lung infiltrates, and symptoms duration in SG. Significant increase in IgM and IgG against SARS-CoV-2 and faster reduction of D-Dimers in SG.	(175)
Probiotics	OR of one URTI with probiotics 0.53 (95% CI 0.37 to 0.76) OR of at least three URTIs with probiotics 0.53 (95% CI 0.36 to 0.80) The mean duration of episodes of URTI with probiotics −1.89 days (95% CI − 2.03 to −1.75) OR for antibiotic prescription for URTI with probiotics 0.65 (95% CI 0.45 to 0.94)	(176)
Probiotics	SMD days of illness per person with probiotics −0.31 (95% CI − 0.41 to −0.11); WMD days of illness with probiotics −0.77 (95% CI − 1.50 to −0.04); SMD days of absence with probiotics −0.17 (95% CI − 0.31 to −0.03)	(177)
*Lactobacillus rhamnosus GG, Lactobacillus casei, Bifidobacterium lactis Bb-12*	Probiotic strains significantly reduced the prevalence of common acute infections and antibiotics utilisation	(178)
*Lactococcus lactis W136*	Probiotic intranasal intervention was correlated with a reduced number of patients showing moderate/severe symptoms of fatigue, loss of perception of smell, and sensation of breathlessness, and by an improved proportion of individuals with moderate/severe facial pain or sore throat	(179)
Probiotics	*L. rhamnosus GG* reduced the duration of RTI − 0.78 days (95% CI − 1.46 to −0.090)	(180)
Probiotics	No effect of probiotics on in-hospital mortality, intensive care unit mortality, duration of hospital stay, duration of intensive care unit stay	(181)
*Lactobacillus rhamnosus* GG	No effect of probiotics on LRTI or overall respiratory infections	(182)
*Probiotic*	Modulation of the intestinal microbiome may have contributed to reduce the severity of COVID-19	(183)
*Ligilactobacillus salivarius*	The study proposed that certain immunological variables might be used as nasal or faecal biomarkers to assess the advantages of a probiotic strain supplementation in the diet of older adult patients infected with SARS-CoV-2	(184)
*Postbiotic*	Products resulting from the metabolic activity of *Lactococcus lactis* and *Lactiplantibacillus plantarum,* such as glucocin F and lactococcine G, respectively, may be administrated as a therapy for inhibiting SARS-CoV-2 infection	(185)
lactobacilli and bifidobacteria	At least one beneficial effect of probiotics were observed in most of the RCTs	(186)
*Lactobacillus paracasei N1115*	Reduced provenance of upper respiratory tract infections, along with a higher percentage of CD3 + cells	(187)
*Postbiotic*	Subtilisin, curvacin A, sakacin P and lactococcin Gb, which are lipopeptides resultant from distinct probiotic strains demonstrated a higher affinity to bind S-protein of SARS-CoV-2 and human ACE2. The amphiphilic nature of lipopeptides might act to restrain the interaction of SARS-CoV-2 with the host ACE2 competitively inhibiting its cell infection	(188)
*Lactobacillus casei strain Shirota*	Healthy subjects reported a significantly lower (22.4%) incidence of respiratory infections than 53.2% in the control group.	(189)
*Lactobacillus plantarum* DR7 suppress proinflammatory cytokines TNF-*α*, IFN-*γ*, enhancing anti-inflammatory cytokines IL-10, IL-4.	Τwo capsules a day (one closed capsule to swallow and one open capsule mixed with maple syrup) from day 1 to 10 and one closed capsule to swallow from day 11 to 25, for maximum 25 days.	(190)
*Lactobacillus rhamnosus GG and Bifidobacterium lactis Bb-12*	The severity of upper respiratory infections was lower in the probiotic group, along with the improved quality of life	(191)
Bifidobacterium strains (25 billion CFUs per capsule), galactooligosaccharides, xylooligosaccharide, and resistant dextrin Treatment for 4 weeks Follow-up for 9 months	Probiotics dramatically lowered the ARGs reservoir in the intestinal microbiome of individuals with COVID-19 infection.	(192)
*Bifidobacterium lactis Bb-12*	Newborn infants receiving probiotics had a lower (65%) incidence of respiratory infections as compared to 94% of infants in the control group	(193)
Probiotic (*Lactobacillus rhamnosus* GG) vs. Placebo	Changes in Shannon bacteria diversity.	(194)
lactobacilli and bifidobacteria	4/10 RCTs reported probiotics reduced incidence of RTI 5/6 RCTs reported probiotics reduced severity of symptoms of RTI 3/9 RCTS reported probiotics shortened duration of RTI	(195)
*Bifidobacterium* spp.*, Lactobacillus* spp.*, Propionibacterium* spp.*, Streptococcus* spp. *and/or α-Hemolytic streptococci*	Decreased the number of days of respiratory tract infection per person	(196)
Probiotics	RR of one RTI with probiotics 0.80 (95% CI 0.82 to 0.96); Days of RTI per child with probiotics −0.16 (95% CI − 0.29 to 0.02) Days absent with probiotics −0.94 (95% CI − 1.72 to −0.15)	(196)
*Lactobacillus rhamnosus HN001, Lactobacillus acidophilus DDS-1, Bifidobacterium lactis Bb-12, and Streptococcus Thermophiles*	Meta-analysis of 23 trials reported that consumption of probiotics reduced the prevalence of respiratory tract infections along with the improved quality of life	(196)
*B. lactis HNO19 L. casei Lc-11 L. plantarum Lp-15 B. lactis B420 B. longum BL05 L. format Lg-36 L. rhamnosus Lr-32 L. paracasei Lpc-37 L. salivarius*	Restoration of intestinal dysbiosis and pulmonary dysfunction. Reduced inflammatory biomarkers: TNF-α, IL-1β, IL-4, and IL-12P70.	(197)
*Lactobacillus rhamnosus CRL1505*	Reduced risk of lung injury and lower virus titer along with modulation of tissue factor and thrombomodulin expression in the lungs of infected mice	(198)
*Bifidobacterium and Lactobacillus.* *Lactobacillus plantarum.* *Lactobacillus reuteri.* *Lactobacillus rhamnosus.* *Bifidobacterium infantis inhibits IL-17.* *Bifidobacterium animali.* *Bifidobacterium lactis.* *Lactococcus lactis.* *Lactobacillus plantarum.*	Beneficial effects against infections (e.g., the influenza virus), including increased helper T cells in the lung parenchyma. Reduce the recruitment of granulocytes and the expression of the proinflammatory cytokines that inhibit the development of pneumonia virus. Increase interferon-γ and interleukin-2. Prevents virus replication. Increase the proportions of total, helper (CD4+), and activated (CD25+) T cells and NK cells in the blood. Activate plasmacytoid dendritic cells. Reduce the tissue damage caused by inflammation in TGEV (gastroenteritis coronavirus).	(199–202)
*Lactobacillus paracasei, Lactobacillus casei 431, and Lactobacillus fermentum PCC*	50 to 60% reduced prevalence of common cold and flu-like symptoms and increased levels of IFN-γ and IgA	(203)

Our research findings, even in studies from 2024, confirm the auxiliary role of probiotics concerning the COVID-19 disease at three levels: in the prevention of the disease, in its duration and the intensity or moderation of its symptoms. The relationship of probiotics is still indirect, and its role is auxiliary; therefore, they were studied and are being studied as an auxiliary method within the framework of a therapeutic strategy.

The central hypothesis guiding this research posits that regulating intestinal microbiota preserves gut health, indirectly enhances systemic immune responses, reduces oxidative stress and inflammation, and supports the absorption of essential micronutrients. These effects collectively benefit the host before and during the disease, reinforcing the potential of probiotics as an adjunctive measure during the COVID-19 pandemic. That fact confirms the co-occurrence and detection of the other keywords included in the red group of the bibliographic map and Table 1. At the same time, it justifies the researcher’s approach to studying the probiotics in that framework.

Two additional studies published in 2024 examined the role of probiotics as an adjunctive approach to treating COVID-19. The first emphasises the use of dietary supplements and the potential benefits against COVID-19 and its symptoms, and the second, a systematic review of the literature, concludes that probiotics should be based on more explicit evidence. However, preliminary studies show beneficial properties for managing the disease.

The first study involved a synthesis of the literature through a PubMed database search to investigate whether dietary and nutritional interventions influence disease progression, recovery time, and symptom severity in COVID-19. Among the findings, probiotics emerged as a notable intervention. Patients receiving the probiotic formula demonstrated complete symptom remission and viral clearance by day 30, experienced no hospitalisations, and exhibited a significant reduction in the duration of symptoms (fever, cough, headache, diarrhoea, and abdominal pain) compared to the control group. The authors reported that combining probiotic strains and omega-3 fatty acids might improve the patient’s overall immune response and respiratory and renal function, particularly in severe disease cases (107).

The second study, employing a comprehensive literature search with criteria including “COVID-19 pandemic” and “probiotics,” identified research trials suggesting that probiotic supplementation reduced antibiotic resistance genes in patients infected with COVID-19. Additionally, patients receiving probiotic strains reported decreased physical and mental fatigue. The study observed that while antibiotic use in COVID-19 patients increased the abundance of antibiotic-resistance genes in the intestinal microbiota, the administration of oral probiotics produced the opposite effect. Available evidence on the impact of probiotic supplementation on preventing COVID-19 infection, altering the patient’s resistome, and reducing the severity of disease symptoms appears promising but was insufficient (108).

Finally, a study from 2024 highlights the actions for the day after the pandemic and the preparedness plans for the next pandemic crisis, which should incorporate innovations and designs in nutrients and probiotics encapsulation for interventions in targeted and vulnerable population groups such as newborns or children (109).

The general function of probiotics in the gastrointestinal tract in humans supports their protective role and mechanism of action against COVID-19. Several systematic reviews published in 2023 highlighted the primary mechanisms of action of probiotics in combating COVID-19. These include inhibiting viral entry through the modulation of enzymes such as angiotensin-converting enzyme 2 and transmembrane serine protease 2, critical for the virus’s binding and entry into human cells. Additionally, probiotics may deter viral invasion by competing for binding sites on epithelial surfaces with pathogenic cells. In addition, probiotics generally contribute to a better response and regulation of the human body’s immunotropic environment, favouring a better treatment of the disease through the possible activation of anti-inflammatory cytokines. In the field of immune response and the general reduction of inflammatory markers, probiotics can diminish the levels of pro-inflammatory cytokines and concurrently improve the levels of anti-inflammatory cytokines, preventing the phenomenon known to occur in commonly observed COVID-19 severe cases, cytokine cascade, i.e., an excessive inflammatory response of the body. Another mechanism of action of probiotics that may indirectly improve the body’s response to the disease is their ability to regulate the proper functioning of the immune system in the mucous membranes of the respiratory tract, thus connecting the gut-lung axis and the general remodelling of the respiratory microflora. Notably, the probiotic strain *Lactobacillus plantarum* has been shown to increase the resistance of respiratory epithelial cells to COVID-19 by stimulating the production of type I and type III interferons (110–113).

These mechanisms of action, which inhibit the full development of the virus, also influence the onset and alleviation of associated symptoms, including diarrhoea, coughing, nausea, and anorexia. Probiotics have also been studied for their potential to reduce vaccine side effects by influencing the gut microbiome (114–116).

Systematic studies confirm probiotics’ effect in mitigating the disease’s symptoms and reducing their severity and intensity in COVID patients. However, researchers are questioning the complete confirmation of this effect through additional clinical trials.

For instance, one study observed that patients who received probiotics during their treatment for COVID-19 experienced shorter hospital stays, improved symptom profiles, and reduced severity of specific symptoms, including diarrhoea, cough, dyspnoea, and shortness of breath (117). On the contrary, for weakness, headache and fever, there was no statistical difference between the group of patients who were administered probiotics and the group who did not take probiotics. As previously mentioned, this improvement was also attributed to the interconnection of the gut-lung axis, with the sample of patients reaching almost 1,200 (118, 119). A smaller-scale study reported improved stress management, mood, and sleep quality. At the same time, another meta-analysis in 2023 confirmed the effect of probiotics on the clinical picture of symptoms among patients taking probiotics. Moreover, the latter study showed statistically significant effects on fever and headache, in contrast to the outcome of the above study (120, 121).

Another group of articles from 2023 focuses on the importance of nutraceuticals and other bioactive ingredients for mitigating complications of COVID-19 (122). Vitamins, minerals, flavonoids, curcumin and probiotics are examined in this context. Probiotics as regulators of the diversity of the intestinal flora can influence the dysbiosis observed in patients treated for up to 6 months after COVID-19. Probiotics functions exert two different immunomodulatory pathways: first, their immunostimulatory function, which involves interleukin (IL)- 12 production, a cytokine that regulates the T-cell triggers interferon-*γ* (IFN-γ) production, and enhances T helper 1 (Th1) differentiation, and second, their immunoregulatory pathway that encompasses IL-10 stimulation and cell activation byTh2, dendritic and B cells, and monocytes for adaptive host’s immunity. Probiotics can also interplay with TLRs on epithelial cells, uprising the generation of cytokines essential for stimulating epithelial cell productivity and preventing apoptosis.

Furthermore, probiotics interact with receptors (TLRs) on epithelial cells, promoting the production of cytokines essential for epithelial cell function and survival while preventing apoptosis. Probiotics may improve disease management when combined with other bioactive components, such as vitamins, minerals, polyphenols, prebiotics, polyunsaturated fatty acids, and compounds derived from medicinal plants. These combinations can aid recovery during and after COVID-19 treatment and indirectly enhance vaccine efficacy through microbiome regulation (123, 124).

Additional systematic reviews investigated the potential of various food groups containing bioactive compounds in mitigating the risk of COVID-19 (125). The primary objective focused on probiotics and other bioactive elements, including essential vitamins (A, C, D, and E), minerals (such as selenium, zinc, and copper), and herbal components (e.g., curcumin, thyme, and oregano). These substances can strengthen the body’s immune system when used in conjunction with a balanced diet, achieving a healthy body weight and following healthy dietary patterns (126, 127).

Recent studies with different approaches to the provision of probiotics therapeutic roles in the treatment of COVID-19 include the hypothesis that the administration of probiotics can help with iron deficiency phenomena, approaching anaemia observed in patients with COVID-19 (128). Its role lies in increasing iron bioavailability and maintaining the integrity of the intestinal mucosa, which helps in iron absorption by producing mucin. In the context of the gut-lung axis, probiotics are hypothesised to exert a protective effect against acute respiratory distress syndrome (ARDS) associated with COVID-19. They may achieve this by modulating the inflammatory response, mitigating the “cytokine storm,” and reducing hyaluronic acid synthesis in the lungs (129). Furthermore, research on the interplay between immune system alterations and long-term COVID-19 complications has emphasised the importance of oral and psychiatric health and the connections between long-term COVID and multi-organ involvement, including the liver, heart, kidneys, brain, and spleen (130). Probiotics may aid in restoring the intestinal microbiota in COVID-19 patients’ post-infection, thereby preventing dysbiosis and its associated complications.

Since 2021, there has been significant anticipation regarding the protective role of probiotics and their potential as adjuvant components in therapeutic regimens against COVID-19. Recent research highlights the prospect of integrating nutritional supplements into future therapeutic strategies, including probiotics and bioactive compounds such as butyrate and desaminotyrosine (131). These substances have been demonstrated to suppress cytokine production, which is also expressed in research this year that confirms their role in inhibiting virus proliferation, regulating the gut-lung axis and enhancing the host’s immune defence against COVID-19. Vitamins, minerals, and omega-3 fatty acids support optimal intestinal function, alleviate clinical symptoms, and mitigate systemic inflammation. Moreover, questions also arise, and further investigation is required to explore the potential synergistic benefits of probiotics in conjunction with vaccination against COVID-19 as in other respiratory illnesses through detailed design of clinical studies with targeted health and non-health population groups and probiotic strain-specific functions (132, 133).

Amid ongoing research efforts, the scientific community underscores the urgency of validating probiotics as an adjuvant therapeutic and preventive approach against COVID-19 through well-designed clinical studies. Emphasis is placed on conducting trials that specify optimal dosages and include a broader range of probiotic strains. Currently, most studies focus on strains from the genera Lactobacillus and Bifidobacterium (134, 135). Despite the need for further validation, the complementary use of probiotics in adjuvant therapy has already been recognised. It should be noted that despite the concern and the wait for validation of the results of probiotics, their complementary use as adjuvant therapy was proposed by the National Health Commission of China and the National Administration of Traditional Chinese Medicine in 2020 (136, 137).

Microbiota-focused interventions, probiotics, and nutritional strategies targeting the gut-lung axis have demonstrated potential in managing COVID-19. This axis has emerged as a critical pathway for understanding immune responses to SARS-CoV-2, as the bidirectional interaction between the gut microbiota and the lungs is crucial for immunological and systemic immune responses (138, 139). As previously discussed, the expression of angiotensin-converting enzyme 2 (ACE2), a key receptor for SARS-CoV-2, links the gut-lung axis. The intestinal microflora, in turn, regulates the activity of ACE2, affecting the entry of the virus and interrupting its multiplication, reducing the total viral load and the inflammatory response. In addition, metabolites derived from the next generation of probiotics and their metabolites enhance immune responses, highlighting the therapeutic potential of modifying the microflora through probiotics. Concurrently, in combination with the prevention of vitamins such as D and omega-3 fatty acids, the intestinal barrier is supported, and inflammation is reduced through their effects on the microbial flora. Understanding the interaction between the intestinal microflora, systemic inflammation, and disease progression could lead to discoveries in managing both acute and chronic diseases (140–142).

The anxiety continued in 2021 for therapeutic means against the coronavirus until we reached the moment of vaccine preparation. In this race, nutritional supplements with potential beneficial effects and prior knowledge of their mode of action against other respiratory diseases or pathogenic conditions are being investigated at length to treat the disease.

The global pandemic has renewed interest in probiotics for their potential to enhance immune function. Given their beneficial function against influenza viruses and other coronaviruses, probiotics have become a research outlet. In parallel with probiotics, prebiotics, which supply beneficial substances by providing a substrate for gut bacteria, complement probiotics by enhancing their colonisation and effectiveness (143). Combining probiotics and prebiotics (synbiotics) has improved outcomes in viral respiratory infections, including COVID-19. These effects consist of promoting intestinal homeostasis, maintaining the microbial flora after the use of antibiotics and reducing susceptibility to secondary infections. Also, the synergistic effect of vitamin D is enhanced by probiotics due to the secretion of lactic acid and lowering the pH value, thus improving its absorption (144, 145). Probiotic-based vaccines have also been proposed as a novel strategy for COVID-19 prevention. Genetically modified probiotics have shown the ability to release immunogenic molecules via mucosal routes, inducing robust systemic and mucosal immune responses, including IgA and IgG production, essential for neutralising respiratory viruses. Further research into strain standardization, dosage, and formulations was considered and formulated again in 2021, although probiotics and prebiotics represent promising, non-invasive strategies for enhancing immune defence against COVID-19 (146).

It is essential to delimit again, now and going back in time, the first year of the start of the pandemic crisis, to shed light on the first critical questions that needed to be answered regarding the research on probiotics and to what extent they could constitute reliable auxiliary solutions to pharmaceutical and nutritional interventions, noting the absence of vaccines. As mentioned earlier, probiotics are already known to be effective in treating other viral respiratory diseases, and there is a grave suspicion of their beneficial role. The starting point is that the gastrointestinal tract hosts diverse microbes that effectively regulate the body’s immune response. Probiotics help to restore or maintain this balance, thus supporting local and systemic immunity. Their mechanism of action has already been identified, namely by promoting regulatory T-cell function and influencing cytokine production; probiotic microorganisms can limit the excessive inflammatory responses characteristic of severe viral infections. In addition, probiotics strengthen epithelial barriers, reducing the possibility of virus translocation through the gut or respiratory mucosa. Moreover, by enhancing antibody responses (IgA, IgG) and supporting antiviral defences, probiotics can help the host to clear the infection more effectively. By nourishing beneficial gut bacteria, prebiotics complement these effects and amplify the positive outcomes associated with probiotics (147). Combining probiotics and prebiotics (synbiotics) holds significant promise for future applications (148). Furthermore, the gut’s interconnectedness with other organs, such as the lungs (gut-lung axis) and the brain (gut-brain axis), highlights the broader implications of gut flora diversity. Enhancing microbial diversity may reduce airway inflammation and modulate neuroinflammation linked to multiple sclerosis and the neurological complications observed in viral infections (149–151).

Another critical parameter that emerged alongside the outbreak of the pandemic crisis and the race for therapeutic measures against the coronavirus is the supportive role of nutritional intervention in patients hospitalised with COVID-19 in the intensive care unit. The management of such patients in the context of nutritional intervention is vital because these patients often present with rapid muscle loss and increased energy requirements. Meeting these needs is complicated by gastrointestinal intolerance and metabolic disorders (152, 153). Therefore, enteral nutrition (EN) is recommended, as it maintains intestinal integrity and reduces the risks of infection. Hence, with their beneficial effects on the intestinal environment, probiotics limit the risk of secondary infections and mitigate the inflammatory complications of severe COVID-19, such as acute respiratory distress syndrome (ARDS). By extension, the need for personalised and tailored nutritional interventions is emphasised to consider individual treatment, side effects of other medications, their dosage, dietary supplements, and parenteral nutrition (154–158).

Reflecting on research conducted during the first year of the COVID-19 pandemic, and with limited data against COVID-19, the scientific community turned to studies on treating respiratory infections, including the role of nutrition in immune functionality in various therapeutic strategies. As documented, nutrition is a critical determinant of immune function, especially during viral infections.

Revisiting the keywords of the same cluster of the bibliographic network found with probiotics, the vitamins are vital for activating immune cells, reducing oxidative stress and strengthening the mucosal barrier. Microelements such as selenium, zinc, and magnesium support antiviral immunity. Finally, the synergy between dietary and lifestyle practices is highlighted. Moderate physical activity enhances both innate and adaptive immunity. Combined with a nutrient-rich diet, it helps reduce the risk of viral infections. Foods rich in polyphenols, unsaturated fatty acids, and probiotics enhance host defences. Public health strategies could incorporate these outcomes by including a balanced diet rich in immune-boosting foods and regular exercise to mitigate the risks of viral infections, such as COVID-19 (159–161).

### 4.3 Limitations

Customarily, all research has some inherent biases and limitations. Therefore, this research faces some challenges, such as the database selection. Although PubMed has purely medical content, additional research incorporating other databases will probably provide more information. In addition, the sole language of choice of the present research, English, which is the international language of the research, indeed excludes other studies that have not yet been digitised or translated and are in the native language of the authors. Finally, since research on COVID issues progresses to increasingly complex problems, further research in the future will shed even more light on the issue of nutrition and its role in different countries and with various research and cultural backgrounds. Despite these shortcomings, we hope that the present study will help future efforts of the global research community.

Although ASReview provided a beneficial framework for speeding up and organizing the article selection process, this machine-learning tool also has some limitations. A key issue is that the tool ranks articles based on the reviewer’s initial, subjective choices. This means that some relevant and important articles may not be identified—especially if they appear later or differ in the features the algorithm “prefers.” This risk is exacerbated when a cut-off criterion is applied, as not all relevant articles may have been viewed yet. In this study, a safer approach was chosen without a cutoff criterion of time or number of articles, but we chose to examine all articles.

Furthermore, while ASReview offers transparency and time savings, it only covers one stage of the systematic review process. Important steps, such as designing the search strategy, data extraction, and risk of bias assessment, remain manual. The tool also relies on predefined settings, such as algorithm selection and using keywords as initial knowledge, which may affect the overall representativeness of the final sample. Finally, the performance of ASReview has not been extensively evaluated across all scientific fields and may vary depending on the topic’s complexity or the users’ experience.

## 5 Conclusion

The bibliometric study presents the dynamics of research formulated during the pandemic crisis and its evolution. The scientific production of research evidence escalated in 2021, reflecting the urgency and demands on issues related to the pandemic and public health, as seen from the research production of reviews and systematic review articles. Subsequently, in the following years, research activity stabilised. However, the higher number of articles on the two above types indicates the importance of continuous integration of knowledge and its centralisation to develop further research activity, its dynamics, and the production of corresponding measures and policies.

As can be seen from the bibliometric maps and the visualisations of keyword networks, nutrition and proper immune function are issues that emerge in COVID research, emphasising interdisciplinarity. New topics that have also emerged spotlight the function of micronutrients, the significance of gut health, and the diverse role of probiotics, suggesting new avenues for research and the adaptability of the global research community to new health crises. Lastly, the diverse role of nutrition and immunity, with probiotics as a connecting link, plays a central role in promoting intersectional efforts to promote public health through research initiatives and constitutes a key foundation for research prioritisation to combat COVID-19 and future infectious diseases globally.

The study proposes specific directions for future research. Clinical trials are needed to assess probiotics’ preventive and therapeutic efficacy in the context of COVID-19 and other respiratory infections, particularly focusing on targeted population groups and strain-specific interventions. Comparative studies should further explore probiotics’ relative and synergistic effectiveness versus other nutritional strategies, including vitamin D, zinc, selenium, and polyphenols. Furthermore, the standardization of probiotic dosing regimens and formulation protocols remains essential to ensure reproducibility, clinical applicability, and preparedness for future pandemics.

In addition, interdisciplinary research that bridges clinical nutrition, immunology, microbiology, and public health is crucial for deepening our understanding of the interaction between diet, microbiota, and immune responses. Longitudinal cohort studies could help elucidate the long-term benefits of nutritional interventions in diverse demographic groups, supporting the design of targeted prevention strategies.

In sum, the bibliometric footprint illuminates the research hierarchy for understanding the multi-level interaction between nutrition, the immune system, and disease management. The current bibliometric approach contributes to the ongoing effort to promote innovation in public health research. The findings serve as a call to action to encourage interdisciplinary collaborations and well-informed citizens to address global health challenges through evidence-based research, with the role of nutrition and immunity as key areas of focus.

## Data Availability

The original contributions presented in the study are included in the article/Supplementary material, further inquiries can be directed to the corresponding author.

## Footnotes

^1^ https://asreview.readthedocs.io/en/latest/screening.html

^2^ https://pubmed.ncbi.nlm.nih.gov/about/

^3^ https://www.mendeley.com/
