# Supplementary material for: The dynamic linkage between covid-19 and nutrition: a review from a probiotics perspective using machine learning and bibliometric analysis
Source: Front Nutr. 2025 May 9;12:1575130. doi: 10.3389/fnut.2025.1575130 (PMC12098087; doi:10.3389/fnut.2025.1575130)
Supplement: Supplementary file 1 [file Data_Sheet_1.pdf]

The dynamic linkage between COVID-19 and nutrition: A review on probiotics perspective using machine learning and bibliometric techniques

Supplementary Material

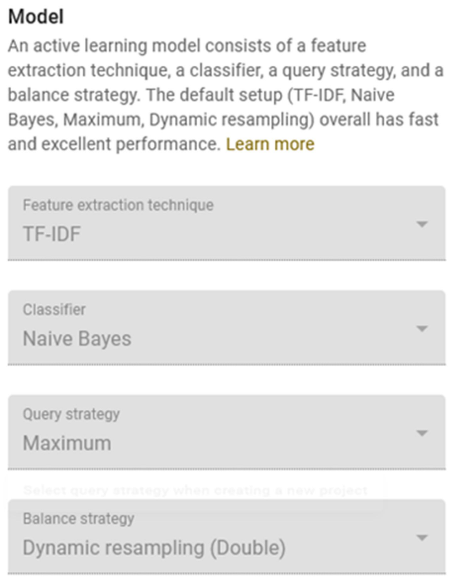

Supplementary Figure 1. ASReview Model Settings and Training.

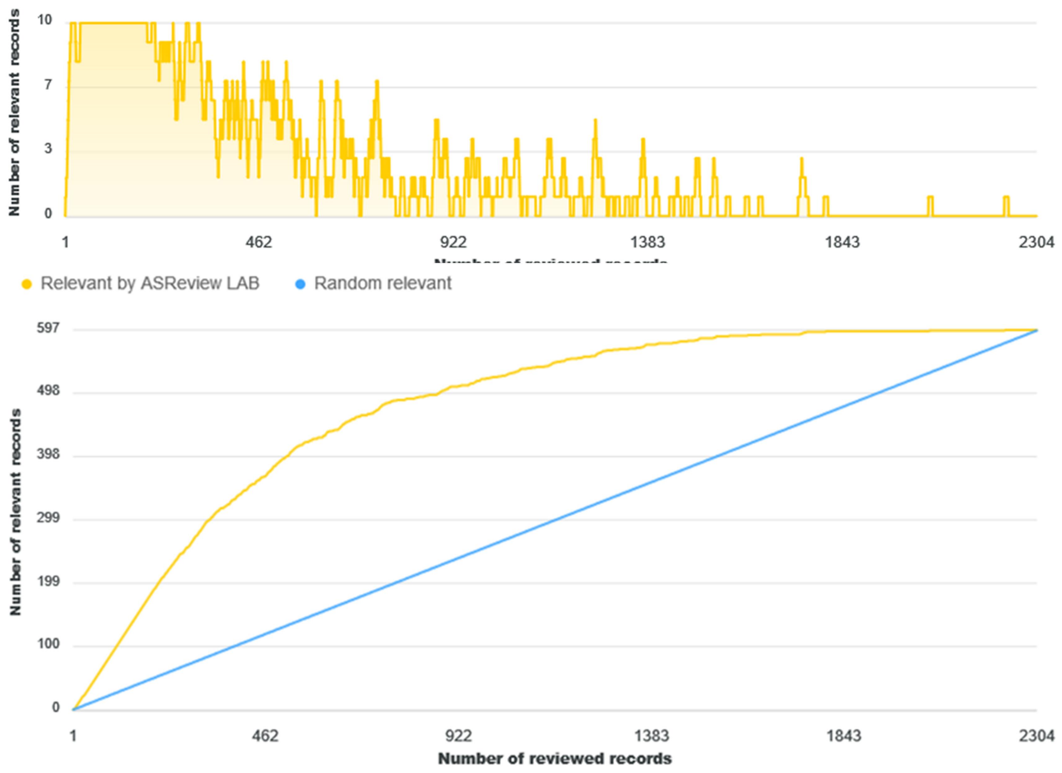

Supplementary Figure 2. ASReview screening process.

Supplementary Table 1. List of the selected articles.

record_id	_of_refere	abstract	ssion_num	custom1	custom2	date	doi	base_prov	edition	number	keywords	language	notes	year	issn	start_page	short_title	condary_ti	title	volume	id
763	JOUR	Iron is a m	34389110		PMC83052	Jul	10.1053/j.	NLM	20210724	3	['*COVID-1	eng	['1532-868	2021	0037-1963	182-187	Iron metal	Semin Hen	Iron metal	58	974
1836	JOUR	The post-C	38269219	The author	PMC1080€	Dec	10.7759/cı	NLM	20231225	12	['covid mic	eng	['2168-818	2023	2168-8184	e51076	Nutritiona	Cureus	Nutritiona	15	1640
611	JOUR	INTRODUC	35169998	The author	PMC8852	Jun	10.1007/s:	NLM	20220215	6	['*covid-1	eng	['1573-497	2022	0301-4851	5153-5163	Ameliorati	Mol Biol R	Ameliorati	49	671
83	JOUR	Increasing	36295814	The author	PMC96112	Sep 27	10.3390/m	NLM	20220927	10	['gut micro	eng	['2218-198	2022	2218-1989 (Print) 221		Gut Microl	Metabolite	Gut Microl	12	1811
1880	JOUR	Inflammat	33105830	The author	PMC7660C	Oct 22	10.3390/m	NLM	20201022	21	['Betacoro	eng	['1420-304	2020	1420-3049		Probiotics	Molecules	Probiotics	25	1530
928	JOUR	BACKGROI	33519087	The author	PMC78338	Feb	10.1016/j.	NLM	20201214		['Diet', 'Nu	eng	['1879-305	2021	0924-2244	187-196	Review art	Trends Foc	Review art	108	1674
220	JOUR	Scientists a	34668465		PMC85934	Dec	10.1017/s(	NLM	20211020	2	['Humans',	eng	['1475-270	2023	0954-4224	181-198	Probiotics	Nutr Res R	Probiotics	36	102
888	JOUR	BACKGROI	33874878			Oct 4	10.2174/1	NLM		2	['Adjuvant:	eng	['2212-401	2021	1872-2083	112-136	Probiotics,	Recent Pat	Probiotics,	15	329
1215	JOUR	Severe acu	35131164	There are	PMC88021	Feb	10.1016/j.	NLM	20220131		['*covid-1	eng	['1879-030	2022	1359-6101	98-107	Roles of th	Cytokine G	Roles of th	63	529
1512	JOUR	COVID-19	33083549	Competing	PMC7536434		10.1038/s	NLM	20201005		['Applied n	eng	['2396-837	2020	2396-8370	17	The poten	NPJ Sci Foc	The poten	4	1559
539	JOUR	Covid-19 is	32430279		PMC72177	Aug	10.1016/j.	NLM	20200513		['Aging', 'B	eng	['1872-749	2020	0168-1702	198018	Gut microl	Virus Res	Gut microl	285	978
40	JOUR	Severe acu	33953641	The author	PMC8059477		10.1155/2	NLM	20210416		['COVID-1	eng	['1466-186	2021	0962-9351	6611222	From the F	Mediators	From the F	2021	421
967	JOUR	The microl	34640553	The author	PMC85091	Sep 30	10.3390/jc	NLM	20210930	19	['Covid-19'	eng	['2077-038	2021	2077-0383 (Print) 207		Microbiota	J Clin Med	Microbiota	10	1753
750	JOUR	It has beer	35379123				10.2174/1	NLM		15	['Humans',	eng	['1873-431	2022	1389-2010	1837-1850	Gut Microl	Curr Pharn	Gut Microl	23	375
61	JOUR	The Coron	36911846	The author	PMC10002	Mar	10.1002/fs	NLM	20230111	3	['Covid-19'	eng	['2048-717	2023	2048-7177	1166-1177	Gut microl	Food Sci N	Gut microl	11	1132
966	JOUR	SARS-CoV-	34579048	The author	PMC8465€	Sep 11	10.3390/n	NLM	20210911	9	['Angioten	eng	['2072-664	2021	2072-6643		Diet, Probi	Nutrients	Diet, Probi	13	955
199	JOUR	Natural he	37299594	The author	PMC10255	Jun 5	10.3390/n	NLM	20230605	11	['Humans',	eng	['2072-664	2023	2072-6643		Functional	Nutrients	Functional	15	592
550	JOUR	COVID-19	37401330			Jun	10.26355/	NLM		12	['Humans',	eng	['2284-072	2023	1128-3602	5927-5945	Role of nu	Eur Rev M	Role of nu	27	189
687	JOUR	COVID-19,	35269795	The author	PMC89107	Feb 28	10.3390/ij	NLM	20220228	5	['COVID-1	eng	['1422-006	2022	1422-0067		Malnutriti	Int J Mol S	Malnutriti	23	387
1395	JOUR	Acute corc	37139365	The author	PMC10150023		10.3389/fp	NLM	20230417		['Humans',	eng	['2296-256	2023	2296-2565	1098774	Gut distres	Front Publ	Gut distres	11	286
1740	JOUR	The SARS-	36145482	The author	PMC95038	Sep 15	10.3390/p	NLM	20220915	9	['Covid-19'	eng	['2076-081	2022	2076-0817 (Print) 207		Gut Microl	Pathogens	Gut Microl	11	1776
1212	JOUR	BACKGROI	34671922	The author	PMC85281	Aug	10.1007/s:	NLM	20211020	4	['*covid-1	eng	['1439-097	2022	0300-8126	815-847	Gut and ai	Infection	Gut and ai	50	89
2021	JOUR	The ecosys	36144365	The author	PMC95058	Aug 31	10.3390/m	NLM	20220831	9	['SARS-Co\	eng	['2076-260	2022	2076-2607 (Print) 207		Immunom	Microorga	Immunom	10	1836
2154	JOUR	The curren	33363049	The author	PMC7756003		10.3389/fc	NLM	20201209		['Animals',	eng	['2235-298	2020	2235-2988	575559	Coronaviru	Front Cell	Coronaviru	10	340
1835	JOUR	BACKGROI	32756323	Authors de	PMC74645	Aug 3	10.3390/jc	NLM	20200803	8	['cirrhosis',	eng	['2077-038	2020	2077-0383 (Print) 207		Gut Microl	J Clin Med	Gut Microl	9	2282
742	JOUR	Since Dece	33925715	The author	PMC81462	Apr 27	10.3390/m	NLM	20210427	5	['SARS CoV	eng	['2076-260	2021	2076-2607 (Print) 207		Next-Gene	Microorga	Next-Gene	9	1720
549	JOUR	On Decem	33160363	The author	PMC76478	Nov 7	10.1186/s:	NLM	20201107	1	['Betacoro	eng	['1479-587	2020	1479-5876	415	COVID-19:	J Transl M	COVID-19:	18	906
132	JOUR	On March	35846832		PMC9246420		10.12938/	NLM	20220329	3	['Dolosigra	eng	['2186-334	2022	2186-6953	94-102	The role of	Biosci Micı	The role of	41	1759
2164	JOUR	The recent	33032673		PMC7684C	Jul 28	10.1017/s(	NLM	20201009	2	['COVID-1	eng	['1475-266	2021	0007-1145	219-227	Mechanisr	Br J Nutr	Mechanisr	126	219
739	JOUR	Worldwide	33845194	The author	PMC80325	May	10.1016/j.	NLM	20210409		['Adaptive	eng	['1521-703	2021	1521-6616	108725	The microl	Clin Immu	The microl	226	83
2167	JOUR	The corona	35004750	The author	PMC8727742		10.3389/fr	NLM	20211222		['Ace2', 'Cc	eng	['2296-858	2021	2296-858X	785496	Potential A	Front Med	Potential A	8	1741
581	JOUR	Interactior	38725230	KD, GW, E(	PMC11093	Jan-Dec	10.1080/1	NLM	20240509	1	['Humans',	eng	['1949-098	2024	1949-0976	2350785	Diet-micro	Gut Microl	Diet-micro	16	2258
2137	JOUR	PURPOSE (	34813042	The author	PMC86095	Dec	10.1007/s:	NLM	20211123	4	['*covid-1	eng	['2161-331	2021	2161-3311	300-306	Impact of (	Curr Nutr I	Impact of (	10	230
1188	JOUR	Low immu	36034155	The author	PMC93931	Oct	10.1016/j.	NLM	20220822		['Active su	eng	['2214-941	2022	1756-4646	105229	Fermentec	J Funct Foc	Fermentec	97	1990
333	JOUR	This reviev	37886807			Oct 27	10.1017/s(	NLM	20231027		['Frailty', 'I	eng	['1475-271	2023	0029-6651	1-16	Nutrition a	Proc Nutr S	Nutrition and immuni		1650
639	JOUR	Due to the	36297256	The author	PMC96072	Oct 18	10.3390/p	NLM	20221018	10	['Covid-19'	eng	['2076-081	2022	2076-0817 (Print) 207		Multidistri	Pathogens	Multidistri	11	1702
1814	JOUR	The proble	35298107	The authors declare no overt anc			10.33029/	NLM	20220111	1	['Animals',	rus	['Sankova,	2022	0042-8833	86-97	[Fermente	Vopr Pitan	[Fermente	91	396
1397	JOUR	COVID-19,	32843118		PMC75423	Apr 28	10.1017/s(	NLM	20200826	8	['COVID-1	eng	['1475-266	2021	0007-1145	851-862	Nutritiona	Br J Nutr	Nutritiona	125	410
1858	JOUR	Probiotics	35844369	The author	PMC92802	May	10.1016/j.	NLM	20220301	5	['Gastroint	eng	['2213-710	2022	1319-562X	3546-3567	Efficacy of	Saudi J Bio	Efficacy of	29	2220
1950	JOUR	COVID-19	33592454		PMC78812	Mar	10.1016/j.	NLM	20210213		['Antiviral	eng	['1879-073	2021	0271-5317	1-12	Probiotics:	Nutr Res	Probiotics:	87	590
1290	JOUR	Severe Acu	37312883	The author	PMC10259402		10.3389/fr	NLM	20230515		['SARS-Co\	eng	['2296-861	2023	2296-861X	1161894	Potential c	Front Nutr	Potential c	10	2171
516	JOUR	Respirator	35462444			Jun	10.1111/1	NLM	20220527	6	['*covid-1	eng	['1348-042	2022	0385-5600	277-291	Probiotics	Microbiol I	Probiotics	66	729
2232	JOUR	BACKGROI	35002079	The author	PMC87203	Feb	10.1016/j.	NLM	20220102		['Diet', 'Fu	eng	['1879-305	2022	0924-2244	174-192	Evidences	Trends Foc	Evidences	120	1801
1541	JOUR	BACKGROI	32940192				10.2174/1	NLM		7	['Animals',	eng	['2212-387	2021	1871-5303	1252-1260	Probiotics	Endocr Me	Probiotics	21	476
221	JOUR	In late Dec	34150807	The author	PMC8211890		10.3389/fr	NLM	20210604		['Ards', 'Co	eng	['2296-858	2021	2296-858X	671714	The Role o	Front Med	The Role o	8	1412
332	JOUR	The role of	34163017	PCC has re	PMC82203	Sep	10.1038/s	NLM	20210623	9	['Aged', '*c	eng	['1476-564	2021	0954-3007	1309-1318	Nutrition a	Eur J Clin N	Nutrition a	75	2
1130	JOUR	Saving live	33785208		PMC79727	Aug	10.1016/j.	NLM	20210319	6	['*COVID-1	eng	['1873-548	2021	0188-4409	582-594	Probiotics	Arch Med	Probiotics	52	814
331	JOUR	The role of	34168111	PCC has re	PMC82235	Jun 23	10.1038/s	NLM	20210623	1	['COVID-1	eng	['2044-405	2021	2044-4052	19	Nutrition a	Nutr Diabe	Nutrition a	11	1
1284	JOUR	Probiotics	35182833			Apr	10.1016/j.	NLM	20220119		['*covid-1	eng	['1873-124	2022	0899-9007	111602	Role of prc	Nutrition	Role of prc	96	1329
468	JOUR	BACKGROI	33588905	The author	PMC78839	Feb 15	10.1186/s:	NLM	20210215	1	['COVID-1	eng	['1824-728	2021	1720-8424	32	Could nutr	Ital J Pedia	Could nutr	47	303

1023	JOUR	The third c	33003648	The autho	PMC7599	Sep 29	10.3390/n	NLM	20200929	10	['Betacoro	eng	['2072-664	2020	2072-6643		Effective I	Nutrients	Effective I	12	308
814	JOUR	Worldwide	33487035			Jan	10.1024/0	NLM	20210125	1	['Aged', '*c	eng	['Gröber, U	2022	0300-9831	13-34	The corona	Int J Vitam	The corona	92	293
520	JOUR	Coronaviru	36012402	The autho	PMC94092	Aug 15	10.3390/ij	NLM	20220815	16	['*covid-19	eng	['1422-006	2022	1422-0067		Nutrition a	Int J Mol S	Nutrition a	23	192
1123	JOUR	Severe acu	34110533	The autho	PMC8190	Aug	10.1007/s	NLM	20210610	4	['COVID-19	eng	['1568-560	2021	0925-4692	1001-1016	Role of vit	Inflammop	Role of vit	29	935
1533	JOUR	Coronaviru	35438892			Mar 17		NLM		1	['*covid-19	eng	['2373-601	2022	0738-0658	29-32	Effects of D	P R Health	Effects of D	41	332
664	JOUR	This review	32785121	The autho	PMC7463	Aug 10	10.3390/jc	NLM	20200810	8	['Covid-19'	eng	['2077-038	2020	2077-0383 (Print) 207		Key Aspec	J Clin Med	Key Aspec	9	931
357	JOUR	Vitamins C	35277048	The autho	PMC8840	Feb 6	10.3390/n	NLM	20220206	3	['Ascorbic A	eng	['2072-664	2022	2072-6643		Multi-Leve	Nutrients	Multi-Leve	14	260
1868	JOUR	BACKGROU	38287379	The autho	PMC1082	Jan 29	10.1186/s	NLM	20240129	1	['Humans',	eng	['2072-131	2024	1606-0997	16	The effect	J Health P	The effect	43	710
141	JOUR	PURPOSE	34837637	The autho	PMC86271	Dec	10.1007/s	NLM	20211127	4	['*covid-19	eng	['2161-331	2021	2161-3311	364-374	The Nutrit	Curr Nutr I	The Nutrit	10	49
46	JOUR	The World	34493880		PMC8412872		10.1007/s	NLM	20210902	4	['Minerals'	eng	['1981-528	2021	0102-695X	361-374	The Role o	Rev Bras F	The Role o	31	1785
1083	JOUR	The corona	34909129	Conflict of	PMC8636	Dec	10.4162/n	NLM	20210628	Suppl 1	['Covid-19'	eng	['2005-616	2021	1976-1457	S1-s21	Nutrient m	Nutr Res P	Nutrient m	15	1279
1803	JOUR	BACKGROU	38907196	The autho	PMC1119	Jun 21	10.1186/s	NLM	20240621	1	['Humans',	eng	['1471-245	2024	1471-2458	1661	Dietary pa	BMC Publi	Dietary pa	24	669
1729	JOUR	Varying de	34629794	Conflict-of	PMC8473	Sep 14	10.3748/w	NLM		34	['Antioxida	eng	['2219-284	2021	1007-9327	5682-5699	Liver disea	World J Ga	Liver disea	27	19
882	JOUR	BACKGROU	34684384	The autho	PMC85381	Sep 26	10.3390/n	NLM	20210926	10	['COVID-19	eng	['2072-664	2021	2072-6643		Patient Nu	Nutrients	Patient Nu	13	7
1939	JOUR	Coronaviru	32975565		PMC75432	Jun 1	10.1093/a	NLM		3	['Aged', '*c	eng	['2156-537	2021	2161-8313	682-692	Coronaviru	Adv Nutr	Coronaviru	12	130
560	JOUR	During the	35355818	There are	PMC8960013		10.1155/2	NLM	20220314		['Child', 'Di	eng	['2314-614	2022	2314-6133	3323825	Multiple M	Biomed Re	Multiple M	2022	758
2251	JOUR	Recently, t	34100300			Jan	10.1024/0	NLM	20210608	1	['*covid-19	eng	['Yaseen, M	2022	0300-9831	35-48	Immunom	Int J Vitam	Immunom	92	362
334	JOUR	Public hea	32340216	P.C.C. has	PMC72307	Apr 23	10.3390/n	NLM	20200423	4	['Covid-19'	eng	['2072-664	2020	2072-6643		Optimal N	Nutrients	Optimal N	12	601
649	JOUR	COVID-19	35901669	The autho	PMC9297	Sep	10.1016/j.	NLM	20220720		['Antioxida	eng	['1878-325	2022	0946-672X	127044	Molecular	J Trace Ele	Molecular	73	1760
272	JOUR	The curren	33244375		PMC7682	Fall		NLM		4	['Covid-19'	eng	['2008-423	2020	2008-2258	331-340	Nutrition,	Gastroent	Nutrition,	13	1179
1228	JOUR	Coronaviru	33947804		PMC82692	May 4	10.1128/m	NLM	20210504	3	['Covid-19'	eng	['2379-507	2021	2379-5077 (Print) 237		Dietary Su	mSystems	Dietary Su	6	1531
1441	JOUR	COVID-19	33544528			Feb 4	10.1515/h	NLM	20210204	1	['Ascorbic A	eng	['1868-189	2021	1868-1883	77-85	COVID-19	Horm Mol	COVID-19	42	131
1786	JOUR	BACKGROU	34729372	Competing	PMC8554510		10.1186/s	NLM	20211029	1	['Covid-19'	eng	['2314-854	2021	2314-8535	71	Vitamins a	Beni Suef U	Vitamins a	10	1603
1264	JOUR	OBJECTIVE	33842400	Conflict of	PMC8021	Jun	10.1007/s	NLM	20210405	1	['Covid-19'	eng	['2251-658	2021	2251-6581	963-972	Nutritiona	J Diabetes	Nutritiona	20	1091
181	JOUR	Low levels	33207753	The autho	PMC7696	Nov 16	10.3390/m	NLM	20201116	22	['Antibodie	eng	['1420-304	2020	1420-3049		Mini-Revie	Molecules	Mini-Revie	25	1290
1590	JOUR	The patho	35063248	Declaratio	PMC8571	Feb	10.1016/j.	NLM	20211106		['Ascorbic A	eng	['2405-457	2022	2405-4577	9-27	Nutritiona	Clin Nutr E	Nutritiona	47	202
503	JOUR	Optimal nu	32653930		PMC7454	Mar 9	10.1093/n	NLM		4	['*covid-19	eng	['1753-488	2021	0029-6643	382-393	Dietary rec	Nutr Rev	Dietary rec	79	193
1077	JOUR	PURPOSE	33835432	None.	PMC8033	Jun	10.1007/s	NLM	20210409	2	['COVID-19	eng	['2161-331	2021	2161-3311	125-136	A Mechani	Curr Nutr I	A Mechani	10	153
281	JOUR	Nutritiona	36099350			Jul 1	10.1684/a	NLM		4	['*COVID-1	fre	['1950-611	2022	0003-3898	319-331	[Review of	Ann Biol C	[Review of	80	316
85	JOUR	OBJECTIVE	32784601	One of the	PMC7468	Aug 7	10.3390/n	NLM	20200807	8	['*Betacor	eng	['2072-664	2020	2072-6643		Early Nutri	Nutrients	Early Nutri	12	239
2174	JOUR	Severe acu	36647652	利益冲突	PMC1040	Jan	10.12182/	NLM		1	['Humans',	chi	['Wang, Xi	2023	1672-173X	108-113	[Nutrition	Sichuan Da	[Nutrition	54	29
1382	JOUR	The novel	36540357		PMC9754	Feb	10.1016/j.	NLM	20221109		['Covid-19'	eng	['2667-268	2023	2667-2685	6-43	An overvie	Clin Nutr C	An overvie	47	943
337	JOUR	Micronutri	34068930	The autho	PMC8155	May 14	10.3390/fc	NLM	20210514	5	['Covid-19'	eng	['2304-815	2021	2304-8158 (Print) 230		A Review o	Foods	A Review o	10	1876
652	JOUR	The world	33059127		PMC7832	Jan	10.1016/j.	NLM	20200908		['COVID-19	eng	['1873-124	2021	0899-9007	111016	Obesity, m	Nutrition	Obesity, m	81	177
1021	JOUR	There are	32842513	The autho	PMC7551	Aug 23	10.3390/n	NLM	20200823	9	['Antioxida	eng	['2072-664	2020	2072-6643		Could Vita	Nutrients	Could Vita	12	1141
691	JOUR	Coronaviru	36245940	None.	PMC95381	Sep 2	10.1002/fs	NLM	20220902	12	['Covid-19'	eng	['2048-717	2022	2048-7177	4112-25	Antioxidar	Food Sci N	Antioxidar	10	1727
381	JOUR	From Pauli	33193359		PMC7655735		10.3389/fi	NLM	20201028		['Adolesce	eng	['1664-322	2020	1664-3224	574029	The Long H	Front Imm	The Long H	11	964
1408	JOUR	Backgroun	34291074	The autho	PMC8287001		10.3389/fr	NLM	20210705		['Covid-19'	eng	['2296-861	2021	2296-861X	698617	Nutritiona	Front Nutr	Nutritiona	8	1282
1019	JOUR	INTRODUC	35432612	Conflict of	PMC8937	Jun	10.2478/sj	NLM	20220321	2	['Covid-19'	eng	['1854-247	2022	0351-0026	124-132	COVID-19	Zdr Varst	COVID-19	61	1991
1916	JOUR	Little is kn	35761885		PMC92227	Jul-Sep	10.1097/ti	NLM	20220622	3	['Covid-19'	eng	['Shoemak	2022	0883-5691	203-217	Immunom	Top Clin N	Immunom	37	1236
1762	JOUR	A healthy	35919576	AUTHOR D	PMC9309	Jun 30	10.3746/p	NLM		2	['Covid-19'	eng	['2287-860	2022	2287-1098	137-149	The Role o	Prev Nutr	The Role o	27	1920
1540	JOUR	PURPOSE	33816060	Conflict of	PMC8010782		10.1007/s	NLM	20210331	3	['25(oh)d3	eng	['2196-304	2021	2196-3045	204-211	Vitamin D	Curr Trop I	Vitamin D	8	1960
680	JOUR	SARS-CoV-	35783349	The autho	PMC9233	Aug	10.1016/j.	NLM	20220625		['Covid-19'	eng	['2667-268	2022	2667-2685	144-154	The effect	Clin Nutr C	The effect	44	1523
119	JOUR	Since COVI	38620928	The autho	PMC8788	Mar	10.1016/j.	NLM	20220125		['Covid-19'	eng	['2666-149	2022	2666-1497	200141	The role o	Hum Nutr	The role o	27	1724
915	JOUR	Severe res	34833042	P.H. is fou	PMC8624	Nov 1	10.3390/li	NLM	20211101	11	['Covid-19'	eng	['2075-172	2021	2075-1729 (Print) 207		Vitamin C	Life (Basel	Vitamin C	11	1789
1353	JOUR	Vitamin C	33916257	The autho	PMC8065	Apr 1	10.3390/n	NLM	20210401	4	['Adult', 'A	eng	['2072-664	2021	2072-6643		Vitamin C	Nutrients	Vitamin C	13	1473
1227	JOUR	The severe	33652653	The autho	PMC7996	Feb 26	10.3390/n	NLM	20210226	3	['*COVID-1	eng	['2072-664	2021	2072-6643		Notable D	Nutrients	Notable D	13	465
194	JOUR	BACKGROU	35217499		PMC8853	May	10.1016/j.	NLM	20220218		['Humans',	eng	['1878-325	2022	0946-672X	126956	Zinc and se	J Trace Ele	Zinc and se	71	105
1564	JOUR	This study	36793782	Conflict of	PMC9900	Jan	10.7762/c	NLM	20230131	1	['Covid-19'	eng	['2287-374	2023	2287-3732	77-89	The Role o	Clin Nutr R	The Role o	12	2017
180	JOUR	Vitamin D	34982377	The autho	PMC8724	Jun	10.1007/s	NLM	20220104	3	['Glucose',	eng	['1573-260	2022	1389-9155	579-599	Associatio	Rev Endoc	Associatio	23	1115
1588	JOUR	Micronutri	35227861	The autho	PMC8873	Apr	10.1016/j.	NLM	20220225		['Covid-19'	eng	['1873-635	2022	0278-6915	112901	The progn	Food Chen	The progn	162	1581

**297**	JOUR	Molecular	34352586		PMC81494	Nov-Dec	10.1016/j.	NLM	20210526		['*covid-19	eng	['1873-124	2021	0899-9007	111356	The possib	Nutrition	The possib	91-92	534
**1946**	JOUR	The coron	35565876	The author	PMC91048	May 2	10.3390/n	NLM	20220502	9	['*covid-19	eng	['2072-664	2022	2072-6643		Do Diet an	Nutrients	Do Diet an	14	94
**289**	JOUR	Introductio	37102680			Nov	10.1089/jii	NLM	20230426	11	['Humans',	eng	['2768-361	2023	2768-3605	695-704	Nutritiona	J Integr Co	Nutritiona	29	844
**748**	JOUR	Coronavir	33990955	The author	PMC82424	Sep	10.1002/jr	NLM	20210519	9	['Age Factc	eng	['1096-907	2021	0146-6615	5285-5294	Vitamin D	J Med Viro	Vitamin D	93	835
**979**	JOUR	OBJECTIVE	38654693	None.	PMC11112	Apr 24	10.1017/s:	NLM	20240424	1	['Adult', 'H	eng	['1475-272	2024	1368-9800	e127	The role of	Public Hea	The role of	27	930
**584**	JOUR	Some earli	34882024				10.1080/1	NLM	20211209	21	['Humans',	eng	['1549-785	2023	1040-8398	5033-5043	Associatio	Crit Rev Fc	Associatio	63	1378
**904**	JOUR	Many stud	35565841	The author	PMC91029	Apr 29	10.3390/n	NLM	20220429	9	['*COVID-1	eng	['2072-664	2022	2072-6643		Modifiable	Nutrients	Modifiable	14	176
**2274**	JOUR	Coronavir	36508011	The author	PMC97431	Apr	10.1007/s(	NLM	20221212	4	['Humans',	eng	['1432-191	2023	0028-1298	607-620	Hospital a	Naunyn Sc	Hospital a	396	1449
**486**	JOUR	BACKGRO	33836322	The author	PMC80103	Jul-Aug	10.1016/j.	NLM	20210331		['Aged', '*c	eng	['1872-697	2021	0167-4943	104411	Nutritiona	Arch Gero	Nutritiona	95	91
**1345**	JOUR	Vitamin D	33142828	J.M. sells v	PMC7692C	Oct 31	10.3390/n	NLM	20201031	11	['*Betacor	eng	['2072-664	2020	2072-6643		Evidence F	Nutrients	Evidence F	12	1161
**712**	JOUR	The pande	32911778	The author	PMC75516	Sep 8	10.3390/n	NLM	20200908	9	['Adolesce	eng	['2072-664	2020	2072-6643		Current St	Nutrients	Current St	12	133
**2025**	JOUR	BACKGRO	35599332	The author	PMC9125C	May 23	10.1186/s	NLM	20220523	1	['*covid-19	eng	['2047-783	2022	0949-2321	70	Zinc suppl	Eur J Med	Zinc suppl	27	17
**1597**	JOUR	There is sti	33146028				10.1080/1	NLM	20201104	5	['Adult', 'A	eng	['1549-785	2022	1040-8398	1308-1316	Vitamin D	Crit Rev Fc	Vitamin D	62	787
**1410**	JOUR	At the end	33803015	The author	PMC80027	Mar 17	10.3390/n	NLM	20210317	3	['COVID-19	eng	['2072-664	2021	2072-6643		COVID-19:	Nutrients	COVID-19:	13	5
**1895**	JOUR	BACKGRO	38693966	The author	PMC11061	May	10.1016/j.	NLM	20240324	5	['Covid-19'	eng	['2475-299	2024	2475-2991	102145	Role of An	Curr Dev N	Role of An	8	1644
**2067**	JOUR	Since the a	35052509	The author	PMC87731	Dec 21	10.3390/a	NLM	20211221	1	['Covid-19'	eng	['2076-392	2021	2076-3921 (Print) 207		Implicatio	Antioxidar	Implicatio	11	1734
**978**	JOUR	BACKGRO	33982105		PMC81946	Jul 1	10.1093/jr	NLM		7	['Anemia/'	eng	['1541-610	2021	0022-3166	1854-1878	The Role o	J Nutr	The Role o	151	41
**1710**	JOUR	BACKGRO	34217144		PMC82364	Jul-Aug	10.1016/j.	NLM	20210628	4	['COVID-19	eng	['1878-033	2021	1871-4021	102189	"Vitamin D	Diabetes N	"Vitamin D	15	187
**58**	JOUR	Worldwide	33570583		PMC74547	Feb 11	10.1093/n	NLM		3	['COVID-19	eng	['1753-488	2021	0029-6643	289-300	Nutritiona	Nutr Rev	Nutritiona	79	158
**446**	JOUR	The pande	34205138	The author	PMC82288	Jun 3	10.3390/n	NLM	20210603	6	['*covid-19	eng	['2072-664	2021	2072-6643		Nutrition i	Nutrients	Nutrition i	13	73
**2132**	JOUR	OBJECTIVE	35938136	The author	PMC9353573		10.3389/fr	NLM	20220722		['Covid-19'	eng	['2296-861	2022	2296-861X	931313	Obesity an	Front Nutr	Obesity an	9	1064
**265**	JOUR	Respirator	35258807	The author	PMC89024	Jun	10.1007/s:	NLM	20220308	6	['*covid-19	eng	['1573-491	2022	0300-8177	1725-1737	Influence (	Mol Cell Bi	Influence (	477	252
**275**	JOUR	SARS-CoV-	36479480		PMC97104	Jun	10.15167/	NLM	20221017	2 Suppl 3	['Humans',	eng	['2421-424	2022	1121-2233	E221-e227	Dietary su	J Prev Mec	Dietary su	63	210
**410**	JOUR	BACKGRO	34719404	The author	PMC85577	Oct 31	10.1186/s:	NLM	20211031	1	['*covid-19	eng	['1475-289	2021	1475-2891	89	Low vitam	Nutr J	Low vitam	20	623
**2235**	JOUR	The newly	34203409	The author	PMC82931	Jun 28	10.3390/d	NLM	20210628	3	['Covid-19'	eng	['2079-972	2021	2079-9721 (Print) 207		The Poten	Diseases	The Poten	9	1766
**115**	JOUR	Insufficien	34069412	W.B.G. rec	PMC81591	May 19	10.3390/n	NLM	20210519	5	['Adult', '*(	eng	['2072-664	2021	2072-6643		COVID-19	Nutrients	COVID-19	13	662
**1750**	JOUR	The nutriti	37528833		PMC10388439		10.1017/jr	NLM	20230726		['Humans',	eng	['2048-679	2023	2048-6790	e86	Selenium s	J Nutr Sci	Selenium s	12	370
**1103**	JOUR	PURPOSE (	37144461			Jul 1	10.1097/m	NLM	20230502	4	['*COVID-1	eng	['1473-651	2023	1363-1950	309-315	Micronutri	Curr Opin	Micronutri	26	47
**1899**	JOUR	Humanity	36284530	The author	PMC95848	Oct	10.1016/j.	NLM	20221021	10	['Food safe	eng	['2405-844	2022	2405-8440	e11216	Effect of fc	Heliyon	Effect of fc	8	1664
**2275**	JOUR	The novel	32438620	The author	PMC72848	May 19	10.3390/n	NLM	20200519	5	['Anti-Infla	eng	['2072-664	2020	2072-6643		COVID-19:	Nutrients	COVID-19:	12	11
**2129**	JOUR	INTRODUC	35086394		PMC88621	Jun	10.1080/1	NLM	20220203	6	['*covid-19	eng	['1744-833	2022	1478-7210	907-913	COVID-19	Expert Rev	COVID-19	20	1255
**2285**	JOUR	The outbre	35044620	The author	PMC87677	Feb	10.1007/s:	NLM	20220119	1	['Adult', 'H	eng	['1559-026	2023	1080-0549	90-107	Risk and P	Clin Rev Al	Risk and P	64	603
**2153**	JOUR	It remains	34626488			May	10.1111/a	NLM	20211027	5	['Adult', '*(	eng	['1398-999	2022	0105-4538	1373-1388	Nutrient s	Allergy	Nutrient s	77	386
**1499**	JOUR	COVID-19	35431568	The author	PMC9012318		10.2147/ij	NLM	20220411		['Covid-19'	eng	['1178-707	2022	1178-7074	3915-3922	Potential c	Int J Gen N	Potential c	15	1966
**803**	JOUR	The COVID	33291720	W.B.G. and	PMC77618	Dec 4	10.3390/n	NLM	20201204	12	['*Athletes	eng	['2072-664	2020	2072-6643		The Benefi	Nutrients	The Benefi	12	232
**588**	JOUR	The immu	35587877		PMC9384C	Oct 2	10.1093/a	NLM		5	['Aged', 'A	eng	['2156-537	2022	2161-8313	1415-1430	Perspectiv	Adv Nutr	Perspectiv	13	790
**329**	JOUR	The Coron	35623808	Declaratio	PMC90125	Jun	10.1016/j.	NLM	20220415		['*covid-19	eng	['2405-457	2022	2405-4577	17-23	Nutrition a	Clin Nutr E	Nutrition a	49	106
**1723**	JOUR	The COVID	37435275	The author	PMC10332	Jun	10.7759/c	NLM	20230610	6	['coronavir	eng	['2168-818	2023	2168-8184	e40231	Zinc Suppl	Cureus	Zinc Suppl	15	1673
**1766**	JOUR	The novel	33919840	The author	PMC80707	Apr 14	10.3390/n	NLM	20210414	4	['Age Factc	eng	['2072-664	2021	2072-6643		Coronavir	Nutrients	Coronavir	13	242
**2068**	JOUR	Since the c	37237970	The author	PMC10215	May 16	10.3390/a	NLM	20230516	5	['Covid-19'	eng	['2076-392	2023	2076-3921 (Print) 207		Antioxidar	Antioxidar	Antioxidar	12	1733
**954**	JOUR	The role of	38171811				10.3177/jr	NLM		6	['Humans',	eng	['1881-774	2023	0301-4800	395-401	Vitamin A	J Nutr Sci V	Vitamin A	69	170
**1368**	JOUR	BACKGRO	35655264	The author	PMC91627	Jun 3	10.1186/s	NLM	20220603	1	['Covid-19'	eng	['2055-092	2022	2055-0928	53	Effective f	BMC Nutr	Effective f	8	1589
**1768**	JOUR	BACKGRO	35581719	Declaratio	PMC91179	Sep	10.1177/0	NLM	20220517	3	['*COVID-1	eng	['2047-945	2022	0260-1060	357-368	Clinical nu	Nutr Healt	Clinical nu	28	842
**2171**	JOUR	The coron	35582132	Conflict-of	PMC90484	Apr 21	10.3748/w	NLM		15	['*covid-19	eng	['2219-284	2022	1007-9327	1526-1535	COVID-19	World J Ga	COVID-19	28	137
**241**	JOUR	BACKGRO	34024545	Declaratio	PMC79875	Jun	10.1016/j.	NLM	20210324		['Anti-Infla	eng	['2405-457	2021	2405-4577	39-48	Strengther	Clin Nutr E	Strengther	43	393
**1716**	JOUR	A symposi	36192012			Oct	10.21873/	NLM		10	['*Biologic	eng	['1791-753	2022	0250-7005	5009-5015	An Apprais	Anticancer	An Apprais	42	457
**1015**	JOUR	BACKGRO	33269357	Competing	PMC77091	Nov 25	10.1101/2	NLM	20201125			eng	['Jolliffe, D	2020			Vitamin D	medRxiv	Vitamin D supplemen		2272
**1155**	JOUR	BACKGRO	35063190	Declaratio	PMC86032	Feb	10.1016/j.	NLM	20211119		['Aftercare	eng	['2405-457	2022	2405-4577	106-116	Strategies	Clin Nutr E	Strategies	47	403
**1014**	JOUR	BACKGRO	33798465	Declaration of interes		May	10.1016/s:	NLM	20210330	5	['Dietary S	eng	['2213-859	2021	2213-8587	276-292	Vitamin D	Lancet Dia	Vitamin D	9	2185
**801**	JOUR	Although c	36145186	W.B.G. rec	PMC95012	Sep 15	10.3390/n	NLM	20220915	18	['*covid-19	eng	['2072-664	2022	2072-6643		Comparing	Nutrients	Comparing	14	1536
**435**	JOUR	Food insec	35162148	The author	PMC88342	Jan 20	10.3390/ij	NLM	20220120	3	['*covid-19	eng	['1660-460	2022	1661-7827 (Print) 166		Micronutri	Int J Envir	Micronutri	19	643

600	JOUR	With the g	34831757	The author	PMC86246	Nov 16	10.3390/ij	NLM	20211116	22	['*covid-19	eng	['1660-460	2021	1661-7827 (Print) 166		The Role o	Int J Envirc	The Role o	18	173
2057	JOUR	BACKGROI	33558789		PMC78579	Apr	10.1016/j.	NLM	20210204		['Foods', 'I	eng	['1879-305	2021	0924-2244	66-77	Role of foc	Trends Foc	Role of foc	110	2136
1302	JOUR	Viral infect	35008706	The author	PMC87456	Dec 28	10.3390/ij	NLM	20211228	1	['Animals',	eng	['1422-006	2021	1422-0067		Role of Sel	Int J Mol S	Role of Sel	23	895
1297	JOUR	Zn deficier	34128459		PMC84385	Apr 28	10.1017/s(	NLM	20210615	8	['Antiviral .	eng	['1475-266	2022	0007-1145	1172-1179	Antiviral a	Br J Nutr	Antiviral a	127	2008
770	JOUR	The antiox	36372013	Declaratio	PMC96303	Jan	10.1016/j.	NLM	20221103		['Humans',	eng	['1878-325	2023	0946-672X	127099	Relationsh	J Trace Ele	Relationsh	75	99
68	JOUR	The novel	33490124	The author	PMC7820179		10.3389/fv	NLM	20210108		['Covid-19'	eng	['2297-176	2020	2297-1769	570748	The Strate	Front Vet S	The Strate	7	1159
1324	JOUR	Existing mi	33235973	Competing	PMC7664499		10.1136/b	NLM	20200618	1	['infectious	eng	['2516-554	2020	2516-5542	93-99	Dietary mi	BMJ Nutr F	Dietary mi	3	1128
502	JOUR	BACKGROI	35272289	The writin	PMC9059C	Mar 10	10.1159/0	NLM	20220310		['Covid-19'	eng	['1421-969	2022	0250-6807	1-8	Long-Chai	Ann Nutr M	Long-Chain Polyunsat		2087
2284	JOUR	Selenium i	32992282	None of th	PMC74813	Oct	10.1016/j.	NLM	20200910		['Animals',	eng	['2213-231	2020	2213-2317	101715	Selenium a	Redox Biol	Selenium a	37	154
2238	JOUR	COVID-19	38255640	All authors	PMC1082C	Dec 22	10.3390/li	NLM	20231222	1	['Covid-19'	eng	['2075-172	2023	2075-1729 (Print) 207		Sarcopenia	Life (Basel	Sarcopenia	14	1689
1744	JOUR	The coron	34064053	The author	PMC82239	May 21	10.3390/n	NLM	20210521	6	['*COVID-1	eng	['2072-664	2021	2072-6643		The Impac	Nutrients	The Impac	13	26
122	JOUR	The purpo	37241870	The author	PMC10222	May 16	10.3390/m	NLM	20230516	10	['Humans',	eng	['1420-304	2023	1420-3049		Could Sele	Molecules	Could Sele	28	1165
1399	JOUR	The global	33183383		PMC77371	Dec	10.1017/s(	NLM	20201113	2	['*covid-19	eng	['1475-270	2021	0954-4224	209-221	Obesity an	Nutr Res R	Obesity an	34	80
906	JOUR	Coronaviru	33065330		PMC75531	Dec	10.1016/j.	NLM	20201013		['Anti-Infla	eng	['2213-717	2020	2213-7165	256-262	Possible ap	J Glob Anti	Possible ap	23	433
941	JOUR	BACKGROI	33645486				10.2174/1	NLM		3	['*covid-19	eng	['1875-641	2022	1573-3998	e0228211	Integrated	Curr Diabe	Integrated	18	1050
845	JOUR	OBJECTIVE	37654858	Authors st	PMC10468	Jun	10.15167/	NLM	20230801	2	['Humans',	eng	['2421-424	2023	1121-2233	E123-e136	Associatio	J Prev Mec	Associatio	64	936
370	JOUR	The high p	33765600	Conflict of	PMC79239	Apr	10.1016/j.	NLM	20210302	4	['COVID-19	eng	['1532-198	2021	0261-5614	1637-1643	Obesity pa	Clin Nutr	Obesity pa	40	857
311	JOUR	Diabetes n	35244834	Authors ha	PMC88953	Apr	10.1007/s:	NLM	20220304	2	['*COVID-1	eng	['1573-260	2022	1389-9155	205-213	Malnutritio	Rev Endoc	Malnutritio	23	121
2227	JOUR	OBJECTIVE	36881729			Jun	10.1080/1	NLM	20230309	6	['Humans',	eng	['1744-833	2023	1478-7210	667-674	Clinical eff	Expert Rev	Clinical eff	21	1454
2086	JOUR	The combi	35775089		PMC91752	Jun	10.22540/	NLM	20220601	2	['Covid-19'	eng	['2459-414	2022	2459-4148	88-94	Nutritiona	J Frailty Sa	Nutritiona	7	1592
1257	JOUR	Some chro	37632513			Nov	10.1053/j.	NLM	20230825	6s	['Humans',	eng	['1532-850	2023	1051-2276	S118-s127	COVID-19	J Ren Nutr	COVID-19	33	1166
1270	JOUR	BACKGROI	36108676			Jul	10.1080/1	NLM	20220928	7	['Humans',	eng	['1744-833	2024	1478-7210	579-585	Effects of s	Expert Rev	Effects of s	22	522
1817	JOUR	A scientific	32992693	The author	PMC77126	Sep 25	10.3390/jp	NLM	20200925	4	['Covid-19'	eng	['2075-442	2020	2075-4426 (Print) 207		Pharmacol	J Pers Mec	Pharmacol	10	1482
532	JOUR	The new c	33573169	The author	PMC79111	Jan 30	10.3390/n	NLM	20210130	2	['COVID-19	eng	['2072-664	2021	2072-6643		Micronutri	Nutrients	Micronutri	13	195
1417	JOUR	SARS-CoV-	35267974	The author	PMC89127	Feb 26	10.3390/n	NLM	20220226	5	['*COVID-1	eng	['2072-664	2022	2072-6643		The Role o	Nutrients	The Role o	14	142
491	JOUR	More than	33783468		PMC80836	Jul 30	10.1093/a	NLM		4	['Aged', '*c	eng	['2156-537	2021	2161-8313	1074-1086	Perspectiv	Adv Nutr	Perspectiv	12	436
562	JOUR	BACKGROI	37781112	The author	PMC10540693		10.3389/fr	NLM	20230915		['Covid-19'	eng	['2296-861	2023	2296-861X	1207237	Role of mi	Front Nutr	Role of mi	10	1672
1918	JOUR	Introductio	33858268			Jun	10.1080/1	NLM	20210426	6	['COVID-19	eng	['1747-635	2021	1747-6348	805-821	Effects of s	Expert Rev	Effects of s	15	497
2008	JOUR	The strong	33801527	The author	PMC79675	Mar 2	10.3390/ij	NLM	20210302	5	['Aged', '*c	eng	['1660-460	2021	1661-7827 (Print) 166		Link betwe	Int J Envirc	Link betwe	18	518
1281	JOUR	Immunom	35267980	The author	PMC89125	Feb 27	10.3390/n	NLM	20220227	5	['Aged', '*c	eng	['2072-664	2022	2072-6643		Healthy Im	Nutrients	Healthy Im	14	264
1844	JOUR	Multiple n	36920723	The author	PMC10015	Apr	10.1007/s:	NLM	20230315	2	['Humans',	eng	['1568-560	2023	0925-4692	573-583	Nutritiona	Inflammop	Nutritiona	31	914
1430	JOUR	Nutrition p	36570149	The author	PMC9772031		10.3389/fr	NLM	20221208		['amino ac	eng	['2296-861	2022	2296-861X	1082500	The relatic	Front Nutr	The relatic	9	1717
905	JOUR	The coron	33390800	Competing	PMC7757136		10.7150/ij	NLM	20210101	2	['COVID-19	eng	['1449-190	2021	1449-1907	314-324	Perspectiv	Int J Med S	Perspectiv	18	280
1952	JOUR	The severe	33037564	The author	PMC75469	Dec	10.1007/s:	NLM	20201010	4	['*Betacor	eng	['1573-910	2020	0921-9668	458-466	Potential I	Plant Food	Potential I	75	251
673	JOUR	Mediterra	34946334	The author	PMC87046	Dec 20	10.3390/m	NLM	20211220	12	['*covid-19	eng	['1648-914	2021	1010-660X (Print) 101		Mediterra	Medicina (	Mediterra	57	379
97	JOUR	Malnutritio	33803339	The author	PMC79674	Mar 9	10.3390/ij	NLM	20210309	5	['Adult', 'A	eng	['1660-460	2021	1661-7827 (Print) 166		Approache	Int J Envirc	Approache	18	408
836	JOUR	Certain mi	38794638	The corres	PMC11123	May 7	10.3390/n	NLM	20240507	10	['Humans',	eng	['2072-664	2024	2072-6643		Individuali	Nutrients	Individuali	16	2183
709	JOUR	As media r	33233560	The author	PMC76997	Nov 20	10.3390/fc	NLM	20201120	11	['Covid-19'	eng	['2304-815	2020	2304-8158 (Print) 230		Food Ingre	Foods	Food Ingre	9	1571
48	JOUR	The patho	36890344	The author	PMC99951	Dec	10.1007/s:	NLM	20230309	12	['Humans',	eng	['1559-072	2023	0163-4984	5546-5560	Mechanist	Biol Trace	Mechanist	201	617
31	JOUR	Coronaviru	34064534	The author	PMC81479	May 4	10.3390/n	NLM	20210504	5	['Age Facto	eng	['2072-664	2021	2072-6643		Emergent	Nutrients	Emergent	13	9
252	JOUR	The whole	32808122	The author	PMC74312	Nov	10.1007/s:	NLM	20200818	11	['Animals',	eng	['1559-118	2020	0893-7648	4856-4877	Therapeut	Mol Neuro	Therapeut	57	1015
218	JOUR	Coronaviru	34322507	The author	PMC8310913		10.3389/fr	NLM	20210712		['SARS-CoV	eng	['2296-861	2021	2296-861X	629440	Dairy-Deri	Front Nutr	Dairy-Deri	8	1542
2149	JOUR	Viral respi	32670091		PMC7326040		10.3389/fp	NLM	20200616		['beneficia	eng	['1664-042	2020	1664-042X	699	The Modu	Front Phys	The Modu	11	2203
2301	JOUR	Covid-19 p	35306019		PMC8924C	Jun 1	10.1016/j.	NLM	20220316	Pt A	['Fatty Acic	eng	['1879-000	2022	0141-8130	244-257	An insight	Int J Biol M	An insight	209	1605
171	JOUR	Viruses pr	37576049	The author	PMC1042C	Aug	10.1002/fs	NLM	20230522	8	['Covid-19'	eng	['2048-717	2023	2048-7177	4444-4459	Antiviral fc	Food Sci N	Antiviral fc	11	2161
149	JOUR	The new c	33084959	The author	PMC75765	Mar	10.1007/s(	NLM	20201021	2	['Adult', 'A	eng	['1436-621	2021	1436-6207	559-579	Micronutri	Eur J Nutr	Micronutri	60	424
1278	JOUR	The vulner	38620788	The author	PMC9762C	Mar	10.1016/j.	NLM	20221219		['Covid-19'	eng	['2666-149	2023	2666-1497	200179	COVID-19	Hum Nutr	COVID-19	31	1549
1400	JOUR	Infection c	32532069	The author	PMC73526	Jun 10	10.3390/n	NLM	20200610	6	['Betacoro	eng	['2072-664	2020	2072-6643		Can Probic	Nutrients	Can Probic	12	831
1581	JOUR	Nutraceuti	36239436	The author	PMC98745	Dec	10.1111/jf	NLM	20221014	12	['Humans',	eng	['1745-451	2022	0145-8884	e14445	Nutraceuti	J Food Bio	Nutraceuti	46	1198
988	JOUR	The emerg	33062058	Competing	PMC7549427		10.1186/s:	NLM	20201012			eng	['1757-474	2020	1757-4749	47	How to bo	Gut Patho	How to bo	12	1816
2091	JOUR	COVID-19	34330463	Declaratio	PMC82342	Aug	10.1016/j.	NLM	20210626		['COVID-19	eng	['2405-457	2021	2405-4577	173-187	The snapsl	Clin Nutr E	The snapsl	44	237

**1999**	JOUR	SARS-CoV2	33468263		PMC78847	Dec 14	10.1017/s(	NLM	20210120	11	['Adaptive	eng	['1475-266	2021	0007-1145	1663-1672	Vitamin A	Br J Nutr	Vitamin A	126	249
**1051**	JOUR	BACKGROI	34118455		PMC81897	Aug	10.1016/j.	NLM	20210610	8	['Autoimm	eng	['1873-018	2021	1568-9972	102865	Autoimmu	Autoimmu	Autoimmu	20	96
**1242**	JOUR	Aging of th	34844781			Jan-Feb	10.1016/j.	NLM	20211126	1	['Aged', '*c	spa	['1578-174	2022	0211-139x	33-38	[Immunos	Rev Esp Ge	[Immunos	57	178
**1645**	JOUR	BACKGROI	35917358	The author	PMC9345473		10.1371/jc	NLM	20220802	8	['Aged', '*(	eng	['1932-620	2022	1932-6203	e0272513	Contempo	PLoS One	Contempo	17	397
**1700**	JOUR	Backgroun	32733487		PMC7363949		10.3389/fi	NLM	20200709		['Actinoba	eng	['1664-322	2020	1664-3224	1548	Role of Im	Front Imm	Role of Im	11	1008
**1834**	JOUR	Microelem	35218585	The author	PMC90825	Mar	10.1002/b	NLM	20220226	2	['*covid-19	eng	['1872-808	2022	0951-6433	294-306	Zinc and g	Biofactors	Zinc and g	48	1413
**608**	JOUR	The newly	33354848			Jun	10.1002/p	NLM	20201222	6	['Anti-Infla	eng	['1099-157	2021	0951-418x	2879-2889	Polypheno	Phytother	Polypheno	35	896
**2036**	JOUR	OBJECTIVE	33039952		PMC78328	Nov-Dec	10.1016/j.	NLM	20200908		['Aged', 'C(	eng	['1873-124	2020	0899-9007	111017	Cohort stu	Nutrition	Cohort stu	79-80	904
**1229**	JOUR	COVID-19	35268723	The author	PMC8912C	Mar 1	10.3390/m	NLM	20220301	5	['COVID-19	eng	['1420-304	2022	1420-3049		The Poten	Molecules	The Poten	27	171
**107**	JOUR	Novel corc	32872374	The author	PMC75514	Aug 28	10.3390/n	NLM	20200828	9	['Antiviral	eng	['2072-664	2020	2072-6643		Antiviral F	Nutrients	Antiviral F	12	903
**1150**	JOUR	Confineme	33520339	The author	PMC7834C	Dec	10.1016/j.	NLM	20201109	4	['Covid-19'	eng	['2414-644	2020	2096-3947	146-152	Lifestyle fa	Glob Healt	Lifestyle fa	4	1926
**1929**	JOUR	Coronaviru	33521076	The author	PMC7838355		10.3389/fv	NLM	20210113		['Ace2', 'Cc	eng	['2297-176	2020	2297-1769	586637	An Insight	Front Vet S	An Insight	7	1707
**2114**	JOUR	The COVID	38498260			Apr	10.1007/s4	NLM	20240318	2	['Animals',	eng	['2299-568	2024	1734-1140	307-327	Effect of p	Pharmacol	Effect of p	76	670
**2110**	JOUR	Despite th	35057437	MM is the	PMC87795	Jan 7	10.3390/n	NLM	20220107	2	['Curcumin	eng	['2072-664	2022	2072-6643		Effectiven	Nutrients	Effectiven	14	1863
**1560**	JOUR	BACKGROI	38810425	Conflict of interest Ov		Jul	10.1016/j.	NLM	20240522	7	['Adult', 'F	eng	['1532-198	2024	0261-5614	1657-1666	The role of	Clin Nutr	The role of	43	1137
**1801**	JOUR	Cancer is n	34080508				10.1080/0	NLM	20210603	2	['Adult', '*(	eng	['1532-791	2022	0163-5581	450-462	Nutrition i	Nutr Canc	Nutrition i	74	44
**404**	JOUR	The curren	34285642	Conflict of	PMC8260C	Jun	10.21315/	NLM	20210630	3	['Covid-19'	eng	['2180-430	2021	1394-195X	18-45	Analysis of	Malays J M	Analysis of	28	1659
**1753**	JOUR	The SARS-(	37324739	The author	PMC10267353		10.3389/fr	NLM	20230601		['Ace-2', 'C	eng	['2296-861	2023	2296-861X	1152254	Nutrients,	Front Nutr	Nutrients,	10	1618
**285**	JOUR	Vitamin D	35334824	The author	PMC89499	Mar 10	10.3390/n	NLM	20220310	6	['Aged', '*(	eng	['2072-664	2022	2072-6643		Calcifediol	Nutrients	Calcifediol	14	2068
**850**	JOUR	BACKGROI	39167591	The author	PMC11338465		10.1371/jc	NLM	20240821	8	['*Diet, Me	eng	['1932-620	2024	1932-6203	e0301564	Relevance	PLoS One	Relevance	19	472
**1741**	JOUR	PURPOSE (	34484977	Conflict of	PMC8404541		10.1007/s4	NLM	20210830	10	['Albumin',	eng	['2167-481	2021	2167-4817	24	Effects of F	Curr Surg F	Effects of F	9	1661
**1962**	JOUR	The virus s	34578858	The author	PMC84721	Aug 27	10.3390/n	NLM	20210827	9	['COVID-19	eng	['2072-664	2021	2072-6643		Covid 19: I	Nutrients	Covid 19: I	13	77
**2063**	JOUR	BACKGROI	37284648	The author	PMC10239816		10.3389/fr	NLM	20230522		['Covid−19	eng	['2296-861	2023	2296-861X	1179432	Probiotics	Front Nutr	Probiotics	10	1998
**2071**	JOUR	Emerging v	35462942	The author	PMC90155	Mar	10.1002/ff	NLM	20211208	1	['Covid-19'	eng	['2643-842	2022	2643-8429	96-123	The direct	Food Front	The direct	3	2113
**1524**	JOUR	Major dep	35662945	The author	PMC9158469		10.3389/fr	NLM	20220518		['S-adenos	eng	['2296-861	2022	2296-861X	867150	Nutrition,	Front Nutr	Nutrition,	9	1554
**1698**	JOUR	Novel corc	34284268		PMC82002	Aug	10.1016/j.	NLM	20210613		['Anti-Infla	eng	['1879-073	2021	0271-5317	109-128	Potential r	Nutr Res	Potential r	92	1016
**2058**	JOUR	PURPOSE (	37646976			Oct	10.1007/s1	NLM	20230830	10	['Humans',	eng	['1534-624	2023	1523-3804	643-652	The Inters	Curr Ather	The Inters	25	70
**2002**	JOUR	PURPOSE (	34506003	The author	PMC84294	Dec	10.1007/s1	NLM	20210910	4	['*COVID-1	eng	['2161-331	2021	2161-3311	352-363	Lifestyle A	Curr Nutr I	Lifestyle A	10	284
**1953**	JOUR	PURPOSE (	32661859	The author	PMC73561	Sep	10.1007/s1	NLM		3	['Betacoro	eng	['2161-331	2020	2161-3311	202-209	Can Vitam	Curr Nutr I	Can Vitam	9	1014
**935**	JOUR	BACKGROI	37010092			Jun	10.1002/n	NLM	20230403	3	['Humans',	eng	['1941-245	2023	0884-5336	499-519	Macronutr	Nutr Clin P	Macronutr	38	389
**1016**	JOUR	OBJECTIVE	36215226	Competing	PMC94493	Sep 7	10.1136/b	NLM	20220907		['*COVID-1	eng	['1756-183	2022	0959-8138	e071230	Effect of a	Bmj	Effect of a	378	1124
**1042**	JOUR	INTRODUC	33137200		PMC76655	Feb 26	10.1093/m	NLM		3-4	['Adult', 'C	eng	['1930-613	2021	0026-4075	e310-e318	Gut Microl	Mil Med	Gut Microl	186	1598
**2033**	JOUR	BACKGROI	35592814	The author	PMC9113266		10.2147/jr	NLM	20220512		['Covid-19'	eng	['1178-239	2022	1178-2390	1101-1110	Associatio	J Multidisc	Associatio	15	2028
**1506**	JOUR	Coronaviru	32667418		PMC7346091		10.31744/	NLM	20200710		['Adolesce	por eng	['2317-638	2020	1679-4508	eRW5774	Gastrointe	Einstein (S	Gastrointe	18	759
**1096**	JOUR	Vitamin D	33230496	Competing	PMC7295862		10.1136/b	NLM	20200520	1	['dietary p	eng	['2516-554	2020	2516-5542	67-73	Avoidance	BMJ Nutr F	Avoidance	3	1911
**1180**	JOUR	BACKGROI	35331502	Declaratio	PMC88096	Apr	10.1016/j.	NLM	20220202		['Aged', '*c	eng	['2405-457	2022	2405-4577	275-281	COVID-19	Clin Nutr E	COVID-19	48	1219
**123**	JOUR	African An	33546262	W.B.G. rec	PMC79133	Feb 3	10.3390/n	NLM	20210203	2	['Black or A	eng	['2072-664	2021	2072-6643		Does the H	Nutrients	Does the H	13	1439
**1782**	JOUR	OBJECTIVE	34202578	All authors	PMC83082	Jun 24	10.3390/n	NLM	20210624	7	['Administ	eng	['2072-664	2021	2072-6643		Effects of a	Nutrients	Effects of a	13	477
**219**	JOUR	This comp	34791425		PMC86899	Feb 10	10.1093/n	NLM		3	['Brain-Gut	eng	['1753-488	2022	0029-6643	561-578	The role of	Nutr Rev	The role of	80	673
**482**	JOUR	PURPOSE (	33909265	All authors	PMC80804	Sep	10.1007/s1	NLM	20210428	3	['*Adipose	eng	['2162-496	2021	2162-4968	214-243	Understan	Curr Obes	Understan	10	715
**1985**	JOUR	The COVID	34065864	The author	PMC81505	May 12	10.3390/n	NLM	20210512	5	['Adolesce	eng	['2072-664	2021	2072-6643		Joint Effor	Nutrients	Joint Effor	13	348
**772**	JOUR	The COVID	37378779				10.1007/9	NLM			['Humans',	eng	['Golpour-I	2023	0065-2598	397-411	Antiviral N	Adv Exp M	Antiviral N	1412	749
**859**	JOUR	The pande	32948484		PMC78319	Dec	10.1016/j.	NLM	20200910	12	['COVID-19	eng	['1876-035	2020	1876-0341	1811-1817	Opinions o	J Infect Pu	Opinions o	13	413
**204**	JOUR	The high ir	35302260	The author	PMC9111C	May	10.1002/p	NLM	20220318	5	['*COVID-1	eng	['1099-157	2022	0951-418X	2042-2060	Therapeut	Phytother	Therapeut	36	86
**1760**	JOUR	Sepsis rem	33925791	The author	PMC81465	Apr 26	10.3390/m	NLM	20210426	5	['*covid-19	eng	['1648-914	2021	1010-660X (Print) 101		Micronutri	Medicina (	Micronutri	57	516
**1431**	JOUR	IMPORTAN	33595634	Conflict of	PMC78904	Mar 16	10.1001/ja	NLM		11	['Adult', 'B	eng	['1538-359	2021	0098-7484	1053-1060	Effect of a	Jama	Effect of a	325	1396
**2297**	JOUR	Coronaviru	38107099	The author	PMC10724	Dec	10.1002/fs	NLM	20230926	12	['Covid-19'	eng	['2048-717	2023	2048-7177	7504-7514	The effect	Food Sci N	The effect	11	1573
**2241**	JOUR	The global	35390469	The author	PMC89772	Jul	10.1016/j.	NLM	20220404		['*COVID-1	eng	['1090-213	2022	0889-1591	19-27	Long COVI	Brain Beha	Long COVI	103	165
**1903**	JOUR	BACKGROI	33155902				10.2174/1	NLM		41	['*Antivira	eng	['1873-428	2020	1381-6128	5241-5260	Current La	Curr Pharr	Current La	26	1085
**305**	JOUR	PURPOSE (	32578027	None.	PMC73086	Sep	10.1007/s1	NLM		3	['Betacoro	eng	['2161-331	2020	2161-3311	119-128	A Rapid Ac	Curr Nutr I	A Rapid Ac	9	426
**2163**	JOUR	Backgroun	32649272			Nov-Dec	10.1080/0	NLM	20200710	8	['Animals',	eng	['1541-108	2020	0731-5724	685-693	Combating	J Am Coll N	Combating	39	652

**1454**	JOUR	Coronaviru	33448317		PMC7834‹	Mar	10.3892/ij	NLM	20210115	3	['Aged', 'A	eng	['1791-244	2021	1107-3756 (Print) 110		Vitamin D,	Int J Mol M	Vitamin D,	47	1379
**1379**	JOUR	BACKGROI	34709541	The author	PMC85524	Dec	10.1007/s:	NLM	20211028	6	['Appetite/	eng	['1568-560	2021	0925-4692	1769-1776	The effect	Inflammop	The effect	29	338
**2092**	JOUR	Severe acu	33102511		PMC7545367		10.3389/fr	NLM	20200925		['Covid-19'	eng	['2296-861	2020	2296-861X	583080	Thrombosi	Front Nutr	Thrombosi	7	1032
**245**	JOUR	Se is a mic	32758306		PMC7503C	Mar 28	10.1017/s(	NLM	20200806	6	['Animals',	eng	['1475-266	2021	0007-1145	618-627	Selenium a	Br J Nutr	Selenium a	125	93
**231**	JOUR	Among the	32823497	The author	PMC74652	Aug 13	10.3390/a	NLM	20200813	8	['Covid-19'	eng	['2076-392	2020	2076-3921 (Print) 207		Hesperidir	Antioxidar	Hesperidir	9	1732
**1712**	JOUR	In this revi	35983618			Feb	10.1017/s(	NLM	20220819	1	['Humans',	eng	['1475-271	2023	0029-6651	1-12	The releva	Proc Nutr S	The releva	82	160
**501**	JOUR	BACKGROI	33744894	The author	PMC8089441		10.1159/0	NLM	20210319	2	['COVID-19	eng	['1662-403	2021	1662-4025	205-213	Patients w	Obes Facts	Patients w	14	425
**2177**	JOUR	The COVID	37764669	The author	PMC10535	Sep 6	10.3390/n	NLM	20230906	18	['Humans',	eng	['2072-664	2023	2072-6643		Stand Up t	Nutrients	Stand Up t	15	696
**1036**	JOUR	PURPOSE (	35391661	All authors	PMC89891	Sep	10.1007/s:	NLM	20220407	3	['*COVID-1	eng	['2162-496	2022	2162-4968	116-125	Obesity, H	Curr Obes	Obesity, H	11	798
**1559**	JOUR	The novel	33218357	The author	PMC76777	Nov 20	10.1186/s:	NLM	20201120	1	['Betacoro	eng	['1479-587	2020	1479-5876	441	The dark s	J Transl M	The dark s	18	246
**2276**	JOUR	Coronaviru	32430996	The author	PMC72768	Nov	10.1002/p	NLM	20200623	11	['*Betacor	eng	['1099-157	2020	0951-418X	2911-2920	Potential e	Phytother	Potential e	34	833
**1052**	JOUR	This syster	33751020		PMC79895	Oct 1	10.1093/a	NLM		5	['*covid-19	eng	['2156-537	2021	2161-8313	1636-1658	Associatio	Adv Nutr	Associatio	12	558
**148**	JOUR	During the	36771480	The author	PMC99202	Feb 2	10.3390/n	NLM	20230202	3	['Humans',	eng	['2072-664	2023	2072-6643		Global Die	Nutrients	Global Die	15	1202
**1607**	JOUR	BACKGROI	34535329	Conflict of	PMC8390C	Dec	10.1016/j.	NLM	20210827	12	['Young Ad	eng	['1532-198	2022	0261-5614	2955-2964	The impac	Clin Nutr	The impac	41	62
**972**	JOUR	The Coron	35866873	The author	PMC93501	Oct	10.1111/jf	NLM	20220722	10	['AMP-Acti	eng	['1745-451	2022	0145-8884	e14345	An overvie	J Food Bio	An overvie	46	1380
**116**	JOUR	Selenium i	36265330	Conflict of	PMC95293	Nov	10.1016/j.	NLM	20221004	11	['Humans',	eng	['1876-035	2022	1876-0341	1225-1233	Selenium a	J Infect Pu	Selenium a	15	81
**317**	JOUR	While all g	32311498		PMC71651	Jul	10.1016/j.	NLM	20200418		['Adaptive	eng	['1090-213	2020	0889-1591	53-54	The impac	Brain Beha	The impac	87	8
**1359**	JOUR	The develc	33041708		PMC74821	May 9		NLM		Suppl 1		eng	['1945-708	2020	1546-993X	54-62	The Functi	Integr Mec	The Functi	19	1901
**864**	JOUR	BACKGROI	38373980	The author	PMC10877	Feb 20	10.1186/s:	NLM	20240220	1	['Humans',	eng	['1475-289	2024	1475-2891	21	Associatio	Nutr J	Associatio	23	591
**1571**	JOUR	The trend	33573173	There is nc	PMC79108	Jan 30	10.3390/n	NLM	20210130	2	['COVID-19	eng	['2072-664	2021	2072-6643		Phytonutri	Nutrients	Phytonutri	13	104
**1735**	JOUR	Dietary ha	32875990				10.2174/1	NLM		7	['Anti-Infla	eng	['2212-387	2021	1871-5303	1171-1182	Exploitatic	Endocr Me	Exploitatio	21	743
**613**	JOUR	The SARS-(	36883129	The author	PMC94456	Mar-Apr	10.1177/1	NLM	20220905	2	['exercise',	eng	['1559-828	2023	1559-8276	231-257	COVID-19	Am J Lifest	COVID-19	17	1669
**1307**	JOUR	'Frailty' ha	36820002	The author	PMC9929193		10.1177/1	NLM	20230213		['Covid-19'	eng	['1759-721	2023	1759-720X	1759720x2	Nutritiona	Ther Adv M	Nutritiona	15	982
**1437**	JOUR	The proce	36027611		PMC93931	Oct	10.1016/j.	NLM	20220822		['Antioxida	eng	['1950-600	2022	0753-3322	113593	The favora	Biomed Ph	The favora	154	1230
**844**	JOUR	Synergistic	32582320		PMC73069	May	10.12669/	NLM		Covid19-s4	['Covid19',	eng	['1681-715	2020	1682-024X	S85-s89	Beyond tra	Pak J Med	Beyond tra	36	1580
**1865**	JOUR	Micronutri	36014944	The author	PMC94163	Aug 21	10.3390/n	NLM	20220821	16	['*covid-19	eng	['2072-664	2022	2072-6643		The Mutua	Nutrients	The Mutua	14	1367
**392**	JOUR	The coron	32738305		PMC73907	Sep	10.1016/j.	NLM	20200730	3	['Anti-Infla	eng	['1872-791	2020	0924-8579	106118	Lactoferrir	Int J Antim	Lactoferrir	56	1187
**657**	JOUR	Multisyste	33763085	The author	PMC7982656		10.3389/fi	NLM	20210308		['Animals',	eng	['1664-322	2021	1664-3224	648546	Vitamin D	Front Imm	Vitamin D	12	412
**1648**	JOUR	PURPOSE (	35703977	DECLARAT	PMC9247C	Jul 1	10.1097/m	NLM		4	['*COVID-1	eng	['1473-651	2022	1363-1950	277-281	Nutritiona	Curr Opin	Nutritiona	25	266
**1914**	JOUR	Viral respi	32570850	The author	PMC73556	Jun 18	10.3390/m	NLM	20200618	6	['Covid-19'	eng	['2076-260	2020	2076-2607 (Print) 207		Microbiota	Microorga	Microbiota	8	1604
**1708**	JOUR	Coronaviru	33158234	The author	PMC76622	Nov 4	10.3390/ij	NLM	20201104	21	['Betacoro	eng	['1660-460	2020	1661-7827 (Print) 166		COVID-19	Int J Envirc	COVID-19	17	726
**371**	JOUR	COVID-19	33105731	T.R.S. decl	PMC76903	Oct 22	10.3390/n	NLM	20201022	11	['Adult', '*I	eng	['2072-664	2020	2072-6643		A Review o	Nutrients	A Review o	12	144
**1574**	JOUR	Worldwide	32786117		PMC74366	Oct	10.1002/n	NLM	20200812	5	['Betacoro	eng	['1941-245	2020	0884-5336	792-799	Relevant N	Nutr Clin P	Relevant N	35	15
**662**	JOUR	The world	34220861	The author	PMC8248499		10.3389/fi	NLM	20210617		['Adjuvant	eng	['1664-322	2021	1664-3224	698672	Glycophos	Front Imm	Glycophos	12	416
**1358**	JOUR	The Medit	36346507	The author	PMC96416	May	10.1007/s:	NLM	20221108	2	['Humans',	eng	['1877-875	2023	1138-7548	371-382	Potential u	J Physiol B	Potential u	79	1254
**1866**	JOUR	BACKGROI	36510155	The author	PMC97431	Dec 12	10.1186/s:	NLM	20221212	1	['Humans',	eng	['1471-244	2022	1471-244x	781	Impact of I	BMC Psych	Impact of I	22	485
**1347**	JOUR	The expan	36839224	The author	PMC99616	Feb 8	10.3390/n	NLM	20230208	4	['Humans',	eng	['2072-664	2023	2072-6643		The Role o	Nutrients	The Role o	15	69
**2077**	JOUR	The persist	35868674		PMC92126	Aug	10.1016/j.	NLM	20220620	3	['Aged', '*(	eng	['1879-885	2022	0749-0690	565-591	Nutraceuti	Clin Geriat	Nutraceuti	38	128
**35**	JOUR	Although (	32668037		PMC74053	Oct	10.1002/n	NLM	20200715	5	['*Betacor	eng	['1941-245	2020	0884-5336	800-805	Gastrointe	Nutr Clin P	Gastrointe	35	826
**1989**	JOUR	The epider	32545556	The author	PMC73538	Jun 12	10.3390/m	NLM	20200612	6	['Betacoro	eng	['1648-914	2020	1010-660X (Print) 101		Nutritiona	Medicina (	Nutritiona	56	197
**2215**	JOUR	COVID-19	37571387	The author	PMC1042C	Aug 4	10.3390/n	NLM	20230804	15	['Humans',	eng	['2072-664	2023	2072-6643		Nutritiona	Nutrients	Nutritiona	15	245
**1510**	JOUR	Backgroun	35268095	The author	PMC89122	Mar 7	10.3390/n	NLM	20220307	5	['*COVID-1	eng	['2072-664	2022	2072-6643		The Effect:	Nutrients	The Effect:	14	32
**243**	JOUR	The height	39339646	D. Berlana	PMC11434	Sep 10	10.3390/n	NLM	20240910	18	['Humans',	eng	['2072-664	2024	2072-6643		Impact of (	Nutrients	Impact of (	16	832
**1747**	JOUR	Studies ha	32653511	Declaratio	PMC73505	Aug 20	10.1016/j.	NLM	20200710		['Anti-Infla	eng	['1873-459	2020	0891-5849	190-199	Potential b	Free Radic	Potential b	156	1405
**614**	JOUR	Calcifediol	35745177	The author	PMC92311	Jun 13	10.3390/n	NLM	20220613	12	['*Asthma/	eng	['2072-664	2022	2072-6643		Calcifediol	Nutrients	Calcifediol	14	1844
**1002**	JOUR	There is lit	33654695		PMC7901705		10.1093/b	NLM	20201221		['Covid-19'	eng	['2321-387	2020	2321-3868	tkaa048	Gut, metal	Burns Trau	Gut, metal	8	1594
**1131**	JOUR	Folic acid (	37309846	The authors declare no conflict o			10.32394/	NLM		2	['Pregnanc	eng	['Kurowska	2023	0035-7715	131-141	Folic acid -	Rocz Panst	Folic acid -	74	325
**829**	JOUR	A large am	37237547	The author	PMC10215	May 18	10.3390/b	NLM	20230518	5	['Covid-19'	eng	['2079-773	2023	2079-7737 (Print) 207		Importanc	Biology (B	Importanc	12	1838
**1543**	JOUR	COVID-19	36505552	There are	PMC9731C	Sep	10.4103/jf	NLM	20221014	9	['COVID-19	eng	['2278-713	2022	2249-4863	4942-4948	Role of Nu	J Family M	Role of Nu	11	1681
**566**	JOUR	Since COVI	35458574	The author	PMC90303	Apr 7	10.3390/m	NLM	20220407	8	['Anti-Infla	eng	['1420-304	2022	1420-3049		The Impor	Molecules	The Impor	27	1711
**1772**	JOUR	The novel	35682411	The author	PMC91806	Jun 2	10.3390/ij	NLM	20220602	11	['*COVID-1	eng	['1660-460	2022	1661-7827 (Print) 166		Lockdown	Int J Envirc	Lockdown	19	222

1485	JOUR	Backgroun	34917577	The authoı	PMC8669368		10.3389/fţ	NLM	20211130		['Aged', '*c	eng	['2296-256	2021	2296-2565	763994	Food Acce	Front Publ	Food Acce:	9	269
397	JOUR	INTRODUC	34330515	Declaratio	PMC81462	Aug	10.1016/j.	NLM	20210525		['COVID-1S	eng	['2405-457	2021	2405-4577	69-77	Nutrition g	Clin Nutr E	Nutrition g	44	155
1562	JOUR	Patients w	32837212		PMC73514	Sep	10.1016/j.	NLM	20200710	4	['Covid-19'	fre	['1279-796	2020	1279-7960	218-224	[Nutritiona	Prat Anest	[Nutritiona	24	1572
646	JOUR	BACKGROI	37439029	Declaratio	PMC10345	Jun	10.1177/0	NLM	20230712	2	['*Diet, Me	eng	['2047-945	2024	0260-1060	215-233	The impac	Nutr Healt	The impac	30	411
1046	JOUR	The recent	36234910	The authoı	PMC9572€	Sep 27	10.3390/m	NLM	20220927	19	['Antiviral .	eng	['1420-304	2022	1420-3049		In Silico Ev	Molecules	In Silico Ev	27	1421
1107	JOUR	The new c	34856087			Nov 30	10.18071/	NLM		11-12	['*covid-1S	hun	['Kovács, A	2021	0019-1442	367-378	[Relationsl	Ideggyogy	[Relationsl	74	196
561	JOUR	BACKGROI	33781275	The authoı	PMC80061	Mar 29	10.1186/s:	NLM	20210329	1	['Adult', 'A	eng	['1479-587	2021	1479-5876	128	The effect	J Transl M	The effect	19	742
792	JOUR	BACKGROI	37408032	The authoı	PMC1032C	Jul 5	10.1186/s:	NLM	20230705	1	['Humans',	eng	['1745-621	2023	1745-6215	442	The effect:	Trials	The effect:	24	429
1608	JOUR	SARS-CoV-	36103031	The authoı	PMC94721	Jul	10.1007/s:	NLM	20220914	5	['Humans',	eng	['1573-683	2023	0272-4340	1685-1695	Neurologi	Cell Mol N	Neurologi	43	22
41	JOUR	BACKGROI	37547100	The authoı	PMC10403319		10.1155/2	NLM	20230728		['Humans',	eng	['1742-124	2023	1368-5031	5734675	Efficacy of	Int J Clin P	Efficacy of	2023	1467
653	JOUR	The aim of	33530554	The authoı	PMC78661	Jan 26	10.3390/ij	NLM	20210126	3	['Animals',	eng	['1422-006	2021	1422-0067		Mediterra	Int J Mol S	Mediterra	22	1246
309	JOUR	A pandem	34071527	The authoı	PMC81981	May 28	10.3390/ij	NLM	20210528	11	['Anti-Infe	eng	['1422-006	2021	1422-0067		The Immu	Int J Mol S	The Immu	22	678
1447	JOUR	Coronavir	33490659		PMC7810€	Jan	10.1016/j.	NLM	20201211	1	['Alternati	eng	['2405-844	2021	2405-8440	e05703	Can limon	Heliyon	Can limon	7	1685
743	JOUR	BACKGROI	37577106	The authoı	PMC10422885		10.1177/2	NLM	20230810		['Long CO\	eng	['2040-623	2023	2040-6223	20406223:	The use of	Ther Adv C	The use of	14	1978
661	JOUR	BACKGROI	35020796		PMC88072	Mar 4	10.1093/aj	NLM		3	['Adult', 'A	eng	['1938-32C	2022	0002-9165	790-798	Effect of a	Am J Clin N	Effect of a	115	909
205	JOUR	The 2019-ı	34979573	The authors declare n		Mar	10.1055/a	NLM	20220103	3	['Antiviral .	eng	['2194-938	2022	2194-9379	123-130	Phytocons	Drug Res (:	Phytocons	72	1070
567	JOUR	Thousand:	33794307	The authoı	PMC80064	Jun	10.1016/j.	NLM	20210329		['Air Pollut	eng	['1873-635	2021	0278-6915	112161	The effect:	Food Chen	The effect:	152	74
1004	JOUR	Severe anc	32391659		PMC88007	May 25	10.3785/j.	NLM		2	['Adrenal C	chi	['Jiang, Sai	2020	1008-9292	158-169	[Pharmace	Zhejiang D	[Pharmace	49	910
2072	JOUR	Patients h	38892564	The authoı	PMC11174	May 26	10.3390/n	NLM	20240526	11	['Humans',	eng	['2072-664	2024	2072-6643		Preventive	Nutrients	Preventive	16	493
834	JOUR	Therapeut	33962011	The authoı	PMC80965	Nov	10.1016/j.	NLM	20210505		['Adrenal C	eng	['1095-913	2021	1046-2023	29-43	Therapeut	Methods	Therapeut	195	938
1474	JOUR	Confineme	33916087	The authoı	PMC80657	Apr 1	10.3390/n	NLM	20210401	4	['Adult', 'B	eng	['2072-664	2021	2072-6643		Conseque	Nutrients	Conseque	13	447
465	JOUR	PURPOSE (	32487876	None.	PMC72991	Jul	10.1097/m	NLM		4	['Covid-19'	eng	['1473-651	2020	1363-1950	288-293	Nutrition i	Curr Opin	Nutrition i	23	1422
489	JOUR	In the last	34876830	The authoı	PMC8643201		10.2147/ji	NLM	20211130		['Covid-19'	eng	['1178-703	2021	1178-7031	6349-6381	Common I	J Inflamm	Common I	14	1996
434	JOUR	Innate imr	33193427		PMC7655931		10.3389/fi	NLM	20201028		['Animals',	eng	['1664-322	2020	1664-3224	590716	Tea Bioact	Front Imm	Tea Bioact	11	405
676	JOUR	Coronavir	35118699			Nov	10.1111/jf	NLM	20220203	11	['Humans',	eng	['1745-451	2022	0145-8884	e14091	Elderberri	J Food Bio	Elderberri	46	949
690	JOUR	Studies ha	37401761	JA and GG	PMC10321	Jan-Dec	10.1080/1	NLM		1	['Humans',	eng	['1949-098	2023	1949-0976	2229938	Limosilact	Gut Microl	Limosilact	15	839
2027	JOUR	Severe acu	34066443	The authoı	PMC81481	May 6	10.3390/v	NLM	20210506	5	['Covid-19'	eng	['2076-393	2021	2076-393X (Print) 207		Probiotic-I	Vaccines (I	Probiotic-I	9	1828
2083	JOUR	An entirel	34621776	The authoı	PMC8490651		10.3389/fr	NLM	20210921		['Covid-19'	eng	['2296-861	2021	2296-861X	747956	Curcumin	Front Nutr	Curcumin	8	2034
154	JOUR	BACKGROI	35668500	MM is the	PMC9167€	Jun 6	10.1186/s:	NLM	20220606	1	['Alkaloids	eng	['1745-621	2022	1745-6215	472	The efficac	Trials	The efficac	23	1938
153	JOUR	BACKGROI	37378780				10.1007/9	NLM			['Humans',	eng	['Askari, Gl	2023	0065-2598	413-426	Evaluation	Adv Exp M	Evaluation	1412	606
1917	JOUR	The main a	36799442			Apr	10.1002/p	NLM	20230217	4	['Humans',	eng	['1099-157	2023	0951-418x	1663-1677	The effecti	Phytother	The effecti	37	1388
1433	JOUR	BACKGROI	36513483	Declaratio	PMC95118	Dec	10.1016/j.	NLM	20220926		['Humans',	eng	['2405-457	2022	2405-4577	431-435	The effect	Clin Nutr E	The effect	52	481
919	JOUR	It has beer	35023260			Feb	10.1002/p	NLM	20220112	2	['*covid-1S	eng	['1099-157	2022	0951-418x	1013-1022	Effect of n	Phytother	Effect of n	36	333
958	JOUR	Zinc is an €	35334884	The authoı	PMC89552	Mar 14	10.3390/n	NLM	20220314	6	['*COVID-1	eng	['2072-664	2022	2072-6643		Developm	Nutrients	Developm	14	231
879	JOUR	Considerin	37429042	The authors of the ma		Nov 1	10.1139/a	NLM	20230710	11	['Humans',	eng	['1715-532	2023	1715-5312	799-807	Possible a	Appl Physi	Possible a	48	1317
1031	JOUR	Corona Vir	35600703	Declaratio	PMC9118892		10.1177/2	NLM	20220514		['Covid-19'	eng	['2050-312	2022	2050-3121	20503121:	Impact of	SAGE Ope	Impact of	10	1884
2096	JOUR	At the end	32613637	The authoı	PMC73614	Dec	10.1002/p	NLM	20200702	12	['Animals',	eng	['1099-157	2020	0951-418X	3137-3147	Naringeni	Phytother	Naringeni	34	258
2266	JOUR	BACKGROI	37271189	Declaratio	PMC10238	Aug	10.1016/j.	NLM	20230603		['Humans',	eng	['1873-696	2023	0965-2299	102958	Adjuvant p	Compleme	Adjuvant p	75	1321
373	JOUR	Novel corc	32574257		PMC7221157		10.3389/fi	NLM	20200507		['Covid-19'	eng	['1664-322	2020	1664-3224	944	Coronavir	Front Imm	Coronavir	11	23
1517	JOUR	There is hi	32703911		PMC73782	Jul 23	10.1128/m	NLM	20200723	4	['Animals',	eng	['2150-751	2020			Can Dietar	mBio	Can Dietar	11	706
1059	JOUR	BACKGROI	33148157				10.2174/1	NLM		2	['Adolesce	eng	['1876-103	2021	1574-8871	146-150	Can Nutrit	Rev Recen	Can Nutrit	16	119
169	JOUR	After the C	39102208				10.1007/9	NLM			['Humans',	eng	['Ayseli, M	2024	0065-2598	349-369	Innovative	Adv Exp M	Innovative	1458	372
1551	JOUR	The sudde	33178237		PMC7593249		10.3389/fţ	NLM	20201015		['clinical tr	eng	['1664-462	2020	1664-462X	568890	Natural Pl	Front Plan	Natural Pl	11	2033
1770	JOUR	COVID19 h	35871955	Declaratio	PMC91662	Aug	10.1016/j.	NLM	20220604		['Ascorbic .	eng	['2405-457	2022	2405-4577	8-14	Vitamin C	Clin Nutr E	Vitamin C	50	1237
302	JOUR	OBJECTIVE	36215222	Competing	PMC94493	Sep 7	10.1136/b	NLM	20220907		['Adult', '*(	eng	['1756-183	2022	0959-8138	e071245	Preventior	Bmj	Preventior	378	599
1603	JOUR	At present	33296131			May	10.1002/p	NLM	20201209	5	['Covid19',	eng	['1099-157	2021	0951-418x	2514-2522	Phytother	Phytother	Phytother	35	1947
299	JOUR	Compared	35912674		PMC10011	Aug 1	10.1017/s(	NLM	20220801	7	['COVID-1S	eng	['1475-266	2022	0007-1145	1-6	Low dietar	Br J Nutr	Low dietar	129	1634
2209	JOUR	Commonly	32593613	Declaratio	PMC7316C	Aug	10.1016/j.	NLM	20200625		['Angioten	eng	['1873-296	2020	0006-2952	114123	Testing of	Biochem P	Testing of	178	1327
1582	JOUR	The recent	37359125	Conflict of	PMC10013	Mar 14	10.1007/s	NLM	20230314		['Antioxida	eng	['2229-447	2023	0970-4078	1-8	Herbs-deri	Vegetos	Herbs-derived phytoc		1721
2007	JOUR	Severe acu	36278162	The authoı	PMC9581083		10.3389/fţ	NLM	20221005		['SARS-Co\	eng	['1663-981	2022	1663-9812	928106	Chinese he	Front Phar	Chinese he	13	1761
1624	JOUR	The aim of	34069656	The authoı	PMC81608	May 19	10.3390/n	NLM	20210519	5	['Adult', 'A	eng	['2072-664	2021	2072-6643		Mediterra	Nutrients	Mediterra	13	720

1413	JOUR	Coronaviru	34335237	The author	PMC8322585		10.3389/fp	NLM	20210716		['Covid-19'	eng	['1663-981	2021	1663-9812	624006	Potentials	Front Phar	Potentials	12	1597
828	JOUR	Ever since	36530229	The author	PMC97531	Dec 13	10.1021/a	NLM	20221202	49		eng	['2470-134	2022	2470-1343	44542-445	Critical Re	ACS Omeg	Critical Re	7	1739
648	JOUR	Coronaviru	34488609				10.2174/1	NLM		3	['Antiviral	eng	['1875-560	2022	1389-5575	457-483	The Effect	Mini Rev M	The Effect	22	1002
1067	JOUR	BACKGROU	33067112	The author	PMC74555	May	10.1016/j.	NLM	20200829		['Antiviral	eng	['1618-095	2021	0944-7113	153311	Screening	Phytomed	Screening	85	1023
1367	JOUR	OBJECTIVE	33272309	The author	PMC77136	Dec 3	10.1186/s:	NLM	20201203	1	['Adult', 'A	eng	['1745-621	2020	1745-6215	996	The effect	Trials	The effect	21	498
318	JOUR	The nutriti	33196946	There is nc	PMC76672	Feb	10.1007/s:	NLM	20201116	1	['Anti-Infec	eng	['1568-560	2021	0925-4692	101-105	COVID-19	Inflammop	COVID-19	29	124
201	JOUR	Rather tha	35287379	The author	PMC8917946		10.1155/2	NLM	20220305			eng	['2090-073	2022	2090-0724	5134560	Possible B	J Nutr Met	Possible B	2022	1883
942	JOUR	Progressio	38620808	The author	PMC96751	Dec	10.1016/j.	NLM	20221119	2	['Covid 19'	eng	['2772-502	2022	2772-5022	100241	Utilization	Appl Food	Utilization	2	2016
1409	JOUR	BACKGROU	36165862	Conflict of	PMC94828	Nov	10.1016/j.	NLM	20220919		['Humans',	eng	['1096-120	2022	0882-4010	105792	The effect	Microb Pat	The effect	172	788
1497	JOUR	Medicinal	36193345	The author	PMC95195	Dec	10.1016/j.	NLM	20220929		['Anthraqu	eng	['0254-629	2022	0254-6299	146-155	Antiviral p	S Afr J Bot	Antiviral p	151	2025
137	JOUR	A clinical s	38282651		PMC10808878		10.1017/jn	NLM	20240123		['Adult', 'H	eng	['2048-679	2024	2048-6790	e5	Virgin coc	J Nutr Sci	Virgin coc	13	628
65	JOUR	Coronaviru	33519274	The author	PMC78321	Mar	10.1016/j.	NLM	20201222	3	['Alternati	eng	['2213-710	2021	1319-562X	1519-1527	Fighting ag	Saudi J Bio	Fighting ag	28	1682
688	JOUR	INTRODUC	36505757	The author	PMC9729036		10.1155/2	NLM	20221130		['Humans',	eng	['1466-186	2022	0962-9351	6850342	The Effect	Mediators	The Effect	2022	1360
236	JOUR	The COVID	38596121	The author	PMC11002	Apr 15	10.1016/j.	NLM	20240330	7	['Covid-19'	eng	['2405-844	2024	2405-8440	e28946	Three year	Heliyon	Three year	10	1631
1286	JOUR	The outbre	33591233				10.1080/1	NLM	20210216	20	['*COVID-1	eng	['1549-785	2022	1040-8398	5569-5581	COVID-19	Crit Rev Fc	COVID-19	62	318
729	JOUR	The COVID	36145270	The author	PMC95044	Aug 25	10.3390/p	NLM	20220825	9	['Covid-19'	eng	['1424-824	2022	1424-8247 (Print) 142		Quercetin	Pharmace	Quercetin	15	1648
1570	JOUR	The use of	35903374	Among inc	PMC9320027		10.3389/fp	NLM	20220711		['*covid-19	eng	['2296-256	2022	2296-2565	821740	Conflict of	Front Publ	Conflict of	10	1169
624	JOUR	COVID-19	35434432	The author	PMC89904	Jul	10.1016/j.	NLM	20220408		['Azadirach	eng	['2468-227	2022	2468-2276	e01184	Anti-COVII	Sci Afr	Anti-COVII	16	1793
628	JOUR	BACKGROU	34668556		PMC8687C	Dec 20	10.1093/ir	NLM		Supplemer	['Banglade	eng	['1537-661	2021	0022-1899	S901-s909	Nutrition a	J Infect Dis	Nutrition a	224	13
778	JOUR	Eating beh	34769648	The author	PMC85828	Oct 22	10.3390/ij	NLM	20211022	21	['*covid-19	eng	['1660-460	2021	1661-7827 (Print) 166		Eating Beh	Int J Envirc	Eating Beh	18	506
681	JOUR	BACKGROU	34271944	The author	PMC82846	Jul 16	10.1186/s:	NLM	20210716	1	['Adult', '*	eng	['1745-621	2021	1745-6215	459	Adapting t	Trials	Adapting t	22	734
1337	JOUR	The conse	34975797	The author	PMC8718880		10.3389/fr	NLM	20211217		['Brics', 'Cc	eng	['1664-302	2021	1664-302X	769884	Interplay c	Front Micr	Interplay c	12	1459
2296	JOUR	PURPOSE (	36249489	Conflict of	PMC9549037		10.1007/s	NLM	20221010	4	['Breast fe	eng	['2196-304	2022	2196-3045	197-206	Effects of (	Curr Trop I	Effects of (	9	1162
609	JOUR	Human he	34274837	The author	PMC82707	Oct 1	10.1016/j.	NLM	20210710		['Biofortifi	eng	['1090-241	2021	0147-6513	112500	Nano-biof	Ecotoxicol	Nano-biof	222	378
2124	JOUR	COVID-19	36823035			Mar	10.1080/0	NLM	20230223	2	['Humans',	eng	['1465-347	2023	0963-7486	124-187	Nutritiona	Int J Food	Nutritiona	74	1097
1001	JOUR	Food acce	38346847			Jun	10.1111/o	NLM	20240212	6	['Humans',	eng	['1467-789	2024	1467-7881	e13720	Use of onli	Obes Rev	Use of onli	25	392
2242	JOUR	With WHC	38045130	The author	PMC10689	Nov	10.1016/j.	NLM	20231117	11	['Covid-19'	eng	['2405-844	2023	2405-8440	e22479	Lesson lea	Heliyon	Lesson lea	9	1738
744	JOUR	OBJECTIVE	32898882	None declared.		Sep	10.1055/s-	NLM	20200908	S 02	['Breast Fe	eng	['1098-878	2020	0735-1631	S46-s53	Managem	Am J Perin	Managem	37	1022
2133	JOUR	Breastfeec	33897707	The author	PMC8058436		10.3389/fi	NLM	20210407		['Antibodie	eng	['1664-322	2021	1664-3224	661806	Breastfeec	Front Imm	Breastfeec	12	3
1505	JOUR	PURPOSE (	36415223	Conflict of	PMC9672601		10.1007/s	NLM	20221118	4	['Breastfee	eng	['2196-304	2022	2196-3045	218-224	Impact of t	Curr Trop I	Impact of t	9	1695
1313	JOUR	BACKGROU	32690158		PMC72116	Aug	10.1016/j.	NLM	20200511		['*Betacor	eng	['2405-457	2020	2405-4577	196-200	Home mec	Clin Nutr E	Home mec	38	951
1093	JOUR	The paper	33344494	The author	PMC7744420		10.3389/fr	NLM	20201203		['COVID-19	eng	['2296-861	2020	2296-861X	598913	COVID-19	Front Nutr	COVID-19	7	1872
273	JOUR	One of the	37376562	The author	PMC10326	May 27	10.3390/v	NLM	20230527	6	['Humans',	eng	['1999-491	2023	1999-4915		Ketogenic	Viruses	Ketogenic	15	288
1530	JOUR	The coron	36470236	The conter	PMC98776	Feb	10.1002/jp	NLM	20221205		['Humans',	eng	['1941-244	2023	0148-6071	S11-s15	Food secu	JPEN J Par	Food secu	47 Suppl 1	960
1565	JOUR	As the CO\	35805748	I.-Y.K. and	PMC92659	Jul 1	10.3390/ij	NLM	20220701	13	['Aged', 'A	eng	['1660-460	2022	1661-7827 (Print) 166		Preventior	Int J Envirc	Preventior	19	1082
246	JOUR	BACKGROU	37437808		PMC10789	Dec	10.1016/j.	NLM	20230716	12	['Adult', 'H	eng	['Bernhart,	2023	2212-2672	1763-1771	"It's Gonna	J Acad Nut	"It's Gonna	123	1313
1010	JOUR	Factors su	36075551		PMC94445	Jan	10.1016/j.	NLM	20220906	1	['Humans',	eng	['2212-267	2023	2212-2672	144-194.e	Changes ir	J Acad Nut	Changes ir	123	1251
1676	JOUR	We evalua	34409727		PMC84202	Oct	10.1111/m	NLM	20210819	4	['Animals',	eng	['1740-870	2021	1740-8695	e13259	Can nutriti	Matern Ch	Can nutriti	17	304
2306	JOUR	The coron	38540923	The author	PMC10969	Mar 19	10.3390/fc	NLM	20240319	6	['COVID-19	eng	['2304-815	2024	2304-8158 (Print) 230		Impact of t	Foods	Impact of t	13	2053
2218	JOUR	BACKGROU	38458559	Declaration of compe		May	10.1016/j.	NLM	20240306		['Adolesce	eng	['1559-203	2024	1551-7144	107490	Design anc	Contemp (	Design anc	140	702
327	JOUR	The COVID	34835945	The author	PMC86181	Oct 20	10.3390/n	NLM	20211020	11	['Adolesce	eng	['2072-664	2021	2072-6643		Telehealth	Nutrients	Telehealth	13	1080
1404	JOUR	Bone fragi	36937822	The author	PMC10015293		10.1177/1	NLM	20230314		['Covid-19'	eng	['1759-721	2023	1759-720X	1759720x2	Bone fragi	Ther Adv M	Bone fragi	15	1574
619	JOUR	INTRODUC	37671267	The author	PMC10475751		10.22037/	NLM	20230723	1	['Covid-19'	eng	['2645-490	2023	2645-4904	e52	Non-Pharr	Arch Acad	Non-Pharr	11	1748
349	JOUR	SARS-CoV-	36615746	The author	PMC98235	Dec 24	10.3390/n	NLM	20221224	1	['Adolesce	eng	['2072-664	2022	2072-6643		The Effect	Nutrients	The Effect	15	109
1248	JOUR	Honey is a	37631069	The author	PMC10459	Aug 14	10.3390/p	NLM	20230814	8	['Covid-19'	eng	['1424-824	2023	1424-8247 (Print) 142		Honey as a	Pharmace	Honey as a	16	1762
1961	JOUR	During the	34065655	The author	PMC81567	May 16	10.3390/n	NLM	20210516	5	['Adolesce	eng	['2072-664	2021	2072-6643		Dietary Ha	Nutrients	Dietary Ha	13	714
1099	JOUR	The COVID	36142043	The author	PMC95172	Sep 18	10.3390/ij	NLM	20220918	18	['Adolesce	eng	['1660-460	2022	1661-7827 (Print) 166		Analysis of	Int J Envirc	Analysis of	19	1347
1378	JOUR	Fasting is (	33521030	The author	PMC7838371		10.3389/fr	NLM	20210113		['coronavir	eng	['2296-861	2020	2296-861X	570235	Ramadan I	Front Nutr	Ramadan I	7	1586
1938	JOUR	PURPOSE (	35194411	Conflict of	PMC8855650		10.1007/s	NLM	20220218	1	['Anaesthe	eng	['2167-627	2022	1523-3855	109-124	Physical ar	Curr Anest	Physical ar	12	2074
934	JOUR	AIMS: Cor	32946851	Declaratio	PMC74921	Nov	10.1016/j.	NLM	20200916		['Aged', 'A	eng	['1872-822	2020	0168-8227	108448	The associ	Diabetes R	The associ	169	361
1135	JOUR	The novel	32472745	The author	PMC73004	Oct	10.1111/m	NLM	20200530	4	['Adult', 'A	eng	['1740-870	2020	1740-8695	e13032	SARS-CoV-	Matern Ch	SARS-CoV-	16	265

**857**	JOUR	A perennia	35631245	The autho	PMC91475	May 18	10.3390/n	NLM	20220518	10	['Antioxida	eng	['2072-664	2022	2072-6643		A Focused	Nutrients	A Focused	14	1461			Departmei	['Hammoudi Halat, D.', 'Krayem, M.', 'Khaled, S.', 'Youn
**152**	JOUR	Until now,	36420866			Feb	10.1002/p	NLM	20221124	2	['Adult', 'H	eng	['1099-157	2023	0951-418x	627-644	Honey and	Phytother	Honey and	37	793			Departmei	['Ashraf, S.', 'Ashraf, S.', 'Ashraf, M.', 'Imran, M. A.', 'Kal
**353**	JOUR	BACKGROI	38236689	Disclosure	PMC10795	Jan 19	10.7189/jc	NLM	20240119		['Child', 'A	eng	['2047-298	2024	2047-2978	05006	Pivoting sc	J Glob Hea	Pivoting sc	14	37			Centre for	['Carducci, B.', 'Dominguez, G.', 'Kidd, E.', 'Janes, K.', 'O
**2293**	JOUR	The global	38890839	The autho	PMC11172	May 22	10.3390/f	NLM	20240522	11	['Covid-19	eng	['2304-815	2024	2304-8158	(Print) 230	Exploring t	Foods	Exploring t	13	2093			Departmei	['Zhou, F.', 'Ma, Z.', 'Rashwan, A. K.', 'Khaskheli, M. B.', '
**955**	JOUR	INTRODU(	36644449	The autho	PMC98309	Feb	10.1016/j.	NLM	20230110		['Covid-19	eng	['2210-804	2023	2210-8033	100626	Senna: As i	J Herb Me	Senna: As	37	1617			Departmei	['Ikram, A.', 'Khalid, W.', 'Saeed, F.', 'Arshad, M. S.', 'Afz
**495**	JOUR	In children	32420946	Each auth	PMC75696	May 11	10.23750/	NLM	20200511	2	['Administ	eng	['2531-674	2020	0392-4203	204-206	COVID-19	Acta Biom	COVID-19	91	913			Array. enz	["D'Auria, E.", 'Anania, C.', 'Cuomo, B.', 'Decimo, F.', 'In
**2173**	JOUR	This analy	32541352		PMC73158	Sep	10.1097/p	NLM		9	['*Betacor	eng	['1537-738	2020	0894-9115	769-774	Physical M	Am J Phys	Physical M	99	898			From the D	['Wang, T. J.', 'Chau, B.', 'Lui, M.', 'Lam, G. T.', 'Lin, N.', '
**878**	JOUR	Nowadays	35930325				10.1080/1	NLM	20220805	3	['Humans',	eng	['1549-785	2024	1040-8398	585-602	Truths and	Crit Rev Fc	Truths and	64	880			Sustainabl	['Hassoun, A.', 'Harastani, R.', 'Jagtap, S.', 'Trollman, H.'
**2054**	JOUR	It is well k	37057872			Jun	10.1002/n	NLM	20230414	3	['Child', 'H	eng	['1941-245	2023	0884-5336	531-538	Impact of	Nutr Clin P	Impact of	38	1138			Outpatient	['Theodotou, K. L.']
**1365**	JOUR	Beta-gluca	34836215	The autho	PMC86237	Nov 6	10.3390/n	NLM	20211106	11	['Agaricale	eng	['2072-664	2021	2072-6643		Beta-Gluca	Nutrients	Beta-Gluca	13	983			Departmei	['Mirończuk-Chodakowska, I.', 'Kujawowicz, K.', 'Witko
**1960**	JOUR	The COVIE	34068876	The autho	PMC81563	May 14	10.3390/n	NLM	20210514	5	['Adolesce	eng	['2072-664	2021	2072-6643		Difference	Nutrients	Difference	13	459			Departmei	['Skolmowska, D.', 'Głąbska, D.', 'Guzek, D.']
**160**	JOUR	COVID-19	34586557		PMC84797	Jan	10.1007/s	NLM	20210929	1	['Animals',	eng	['1993-040	2022	1672-0415	88-95	From Mole	Chin J Inte	From Mole	28	383			Departmei	['Atif, M.', 'Naz, F.', 'Akhtar, J.', 'Imran, M.', 'Saleem, S.',
**2032**	JOUR	Green tea	34899956	The autho	PMC8664505		10.1155/2	NLM	20211203			eng	['1741-428	2021	1741-427X	7170736	A Compret	Evid Basec	A Compret	2021	2175			Departmei	['Tallei, T. E.', 'Fatimawali', 'Niode, N. J.', 'Idroes, R.', 'Z
**56**	JOUR	The coron	34786156	Conflict-of	PMC85674	Oct 26	10.4252/w	NLM		10	['Covid-19	eng	['1948-021	2021	1948-0210	1530-1548	Overview (	World J St	Overview (	13	892			Departmei	['Akbulut, G.', 'Yesildemir, O.']
**1809**	JOUR	On 11 Mar	34960042	The autho	PMC87056	Dec 15	10.3390/n	NLM	20211215	12	['COVID-1!	eng	['2072-664	2021	2072-6643		"Five Keys	Nutrients	"Five Keys	13	117			Food & He	['San Onofre, N.', 'Soler, C.', 'Merino-Torres, J. F.', 'Sori
**1329**	JOUR	In additior	33393841	No compe	PMC78264	Jan	10.1089/b	NLM	20201230	1	['*Benchm	eng	['1556-834	2021	1556-8253	29-38	Best Practi	Breastfeec	Best Pract	16	384			Margaret F	['McGuire, M. K.', 'Seppo, A.', 'Goga, A.', 'Buonsenso, D
**1494**	JOUR	The currer	32518634	Competing	PMC7255895		10.12688/	NLM	20200207		['Animals',	eng	['2046-140	2020	2046-1402	93	Lithium an	F1000Res	Lithium an	9	841			Departmei	['Nowak, J. K.', 'Walkowiak, J.']
**1102**	JOUR	COVID-19	33038834		PMC75278	Nov	10.1016/j.	NLM	20201001		['Betacoro	eng	['1532-282	2020	0952-3278	102183	Polyunsatu	Prostaglan	Polyunsatu	162	994			Dell Pediat	['Kothapalli, K. S. D.', 'Park, H. G.', 'Brenna, J. T.']
**1926**	JOUR	OBJECTIVE	38151866	Fernandez-Aranda acknowledges			10.1007/s	NLM		12	['Humans',	eng	['1760-478	2023	1279-7707	1162-1167	Intensive V	J Nutr Hea	Intensive V	27	1228			Prof Jordi S	['Shyam, S.', 'García-Gavilán, J. F.', 'Paz-Graniel, I.', 'Gaf
**117**	JOUR	COVID-19	36066798	The autho	PMC94465	Oct	10.1007/s	NLM	20220906	47	['*Air Pollu	eng	['1614-749	2022	0944-1344	70822-708	The impac	Environ Sc	The impac	29	608			Al Ahsa Mu	['Alshubaith, I. H.', 'Alhajri, S.', 'Alhajri, A.', 'Alsultan, R. A
**288**	JOUR	The ongoi	33669146	The autho	PMC79965	Feb 25	10.3390/f	NLM	20210225	3	['food loss	eng	['2304-815	2021	2304-8158	(Print) 230	Transform	Foods	Transform	10	1809			Food Engir	['Boyacı-Gündüz, C. P.', 'Ibrahim, S. A.', 'Wei, O. C.', 'Ga
**1500**	JOUR	Recently, t	37810195	Yingqiu Xie	PMC93801	Sep	10.1097/h	NLM	20210907	1	['Chemical	eng	['2765-861	2021	2097-0226	31-38	Rhizoma p	Acupunct I	Rhizoma p	1	1654			School of S	['Nurtay, L.', 'Sun, Q.', 'Mu, C.', 'Cao, Z.', 'Wang, Q.', 'Lia
**1752**	JOUR	COVID-19,	34306988	Conflict of	PMC8279807		10.1007/s	NLM	20210715	4	['Antimala	eng	['2198-641	2021	2198-641X	135-149	Phytocom	Curr Pharn	Phytocom	7	1814			Faculty of	['Rolta, R.', 'Salaria, D.', 'Sharma, P.', 'Sharma, B.', 'Kum
**1256**	JOUR	Considerin	32952005		PMC74344	Jan	10.1053/j.	NLM	20200818	1	['COVID-1!	eng	['1532-850	2021	1051-2276	39-42	Coronavirı	J Ren Nutr	Coronavirı	31	1260			Federal Ur	['Mafra, D.', 'Cardozo, Lfmf', 'Moraes, C.', 'Moreira, L. S

Supplementary Table 2. List of all nutrition, probiotics, and COVID-19 studies with Review Status: "Ongoing" retrieved from the PROSPERO database on 24/12/2024.

COVID-19 and nutrition		
**Registered**	Title	Review status
**24/3/2020**	The relationship between the blood D-dimer and severe coronavirus disease 2019 (COVID-19) infections: a meta-analysis [CRD42020175553]	Review Ongoing
**20/4/2020**	The prevalence and influence of chronic comorbidities in patients with COVID-19: a systematic review and meta-analysis [CRD42020177203]	Review Ongoing
**21/4/2020**	Effect and safety of chloroquine and hydroxychloroquine in the treatment of COVID-19: systematic review and meta-analysis of the evidence from studies with humans [CRD42020181282]	Review Ongoing
**5/5/2020**	Short and long term impacts of school closures, or other isolation measures in childhood, on physical and mental health [CRD42020181658]	Review Ongoing
**24/4/2020**	Lifestyle factors and COVID-19: a rapid and living systematic review and meta-analysis. [CRD42020181902]	Review Ongoing
**6/5/2020**	Risk factors for severe illness and death in COVID 19: a systematic review and meta-analysis [CRD42020184440]	Review Ongoing
**11/8/2020**	Short-term effectiveness of nutritional therapy to treat type 2 diabetes mellitus in low- and middle-income countries: a systematic review of randomised controlled trials [CRD42020188435]	Review Ongoing
**3/6/2020**	Incidence, risk factors and etiology of clinical organ transplant related to COVID-19 [CRD42020189458]	Review Ongoing
**24/6/2020**	A Healthy Immunity May be the Only Option for Patients with COVID-19 After a Graded Systematic Review of Investigational COVID-19 Therapies [CRD42020190300]	Review Ongoing

**12/8/2020**	Recommendations for the prevention of contagion by the new coronavirus among health professionals in the scenario of COVID-19: a systematic review [CRD42020190491]	Review Ongoing
**15/6/2020**	A systematic review of COVID-19 treatment trials to assess whether they adhere to ethical standards: protocol for a systematic review [CRD42020191823]	Review Ongoing
**22/6/2020**	Diagnostic accuracy of pooled nucleic acid amplification testing for SARS-CoV-2 infection [CRD42020192697]	Review Ongoing
**17/7/2020**	Nonspecific effect of vaccines on mortality and prevention of nonspecific diseases: a systematic review and meta-analysis of randomized controlled trials [CRD42020197873]	Review Ongoing
**20/7/2020**	Vertical transmission of COVID-19 through breastfeeding: a living systematic review protocol [CRD42020199542]	Review Ongoing
**11/8/2020**	Interventions to support early childhood development in times of COVID-19: a systematic review and evidence gaps [CRD42020202541]	Review Ongoing
**18/8/2020**	Association of vitamin D status with COVID-19 infection and severity: a systematic review of observational studies [CRD42020203903]	Review Ongoing
**14/9/2020**	Nutritional intake and peripheral arterial occlusive disease. [CRD42020204398]	Review Ongoing
**10/9/2020**	Prevelence of depression, anxiety, sleep disorders and posttraumatic stress symptoms among children during the COVID-19 pandemic: a systematic review and meta-analysis [CRD42020205166]	Review Ongoing
**11/1/2021**	Is nutritional supplementation associated with the prevention for the community-acquired lower respiratory tract infections in generally healthy but nutritionally insufficient adults and elderly? [CRD42021205604]	Review Ongoing
**15/9/2020**	In patients hospitalised with COVID-19 infection, what is the best way of ensuring continuity of nutritional care on hospital discharge to minimise the nutritional consequences of infection and optimise recovery? [CRD42020208448]	Review Ongoing
**17/9/2020**	Administration of parenteral vitamin C in patients with severe infection: a systematic review and meta-analysis [CRD42020209187]	Review Ongoing
**23/10/2020**	Prevalence and outcomes of malnutrition among hospitalized patients with COVID-19: a systematic review and meta-analysis [CRD42020215396]	Review Ongoing
**12/11/2020**	Prevalence of gastrointestinal and gustatory manifestations in adults and the elderly with Covid-19: a systematic review and meta-analysis [CRD42020219513]	Review Ongoing
**13/11/2020**	Prognostic value of immune and inflammatory biomarkers for COVID-19 severity and mortality: a systematic review and meta-analysis. [CRD42020220214]	Review Ongoing
**16/11/2020**	External negative pressure ventilation for the treatment of acute or acute-on-chronic respiratory failure: a systematic review [CRD42020220881]	Review Ongoing
**27/11/2020**	Adverse perinatal outcomes, nurturing care and early childhood development during the pandemic of COVID-19: A systematic review and meta-analysis [CRD42020222333]	Review Ongoing

**11/12/2020**	A systematic review and meta-analysis of observation and intervention studies on the prevention of upper respiratory tract infection: implications for the role of nutrient intake [CRD42020222530]	Review Ongoing
**26/12/2020**	Understanding the role of the volunteer in palliative care in Scandinavia - a systematic review and thematic analysis of qualitative studies of the experiences of volunteers' role in palliative care, from the perspectives of volunteers, patients and the patient's family and healthcare professionals [CRD42020222695]	Review Ongoing
**4/12/2020**	COVID-19 and Maternal Mortality: Evidence synthesis on risks and risk factors for maternal deaths [CRD42020224120]	Review Ongoing
**8/12/2020**	Predictors of COVID-19 outcomes: an individual participant meta-analysis [CRD42020224323]	Review Ongoing
**11/12/2020**	The association between Vitamin D supplementation and COVID-19 severity and mortality [CRD42020224330]	Review Ongoing
**11/12/2020**	Impact of confinement resulting from the COVID-19 pandemic on diet quality and eating behaviours: a systematic review of the literature [CRD42020225292]	Review Ongoing
**21/12/2020**	Epidemiology of psychological consequences of COVID-19 in Africa: a systematic review and meta-analysis of studies from Africa [CRD42020227292]	Review Ongoing
**11/1/2021**	Weight excess association with severity in children and adolescents with COVID-19: a systematic review [CRD42021230686]	Review Ongoing
**22/2/2021**	Determinants of Food Insecurity and Physical Activity: A Systematic Review [CRD42021231672]	Review Ongoing
**3/3/2021**	Persistent symptoms following SARS-CoV-2 infection amongst children and young people: a meta-analysis of controlled and uncontrolled studies [CRD42021233153]	Review Ongoing
**1/2/2021**	Effects of COVID-19 candidate vaccines or their components on pregnancy safety outcomes [CRD42021234185]	Review Ongoing
**1/2/2021**	A systematic review of the symptoms associated with the UK COVID-19 variant B117 and related clinical outcomes across Black, Asian and minority ethnic (BAME) groups [CRD42021234358]	Review Ongoing
**22/2/2021**	A living systematic review of the impacts of COVID-19 infection on the health-related quality of life across Black, Asian and minority ethnic (BAME) groups [CRD42021238645]	Review Ongoing
**20/4/2021**	Acute acalculous cholecystitis in patients with Covid-19 infection: a systematic review [CRD42021240016]	Review Ongoing
**12/3/2021**	The effect of COVID-19 related restrictions on diet and physical activity in older and elderly populations: a systematic review [CRD42021241997]	Review Ongoing
**16/3/2021**	The impact of gut microbiota and modulators on lung immunity and pathogenicity of respiratory tract infections: an integrated systematic review [CRD42021242779]	Review Ongoing
**28/5/2021**	The impact of gut microbiota and modulators on lung immunity and pathogenicity of respiratory tract infections in animal models and in vitro systems: an integrated systematic review and meta-analysis [CRD42021242785]	Review Ongoing
**3/5/2021**	Does SARS CoV-2 have hepatotropic properties? Meta-analysis of observational studies [CRD42021242958]	Review Ongoing

**27/5/2021**	Combined non-pharmaceutical and lifestyle approaches for the global prevention and treatment of depression in adults, a systematic review [CRD42021243259]	Review Ongoing
**28/8/2021**	Supplementation therapy to minimise nutrition- and diet-related complications of bariatric surgery. [CRD42021244336]	Review Ongoing
**4/6/2021**	Systematic review of 24-hour movement behaviors among children and youth in Asian countries (2-17 years) [CRD42021249327]	Review Ongoing
**12/5/2021**	The prevalence of household food insecurity in Canada during the COVID-19 pandemic and mitigation strategies to reduce it: a systematic review [CRD42021254450]	Review Ongoing
**20/5/2021**	Vitamin C and D supplementation and the severity of COVID-19: a protocol for systematic review and meta-analysis [CRD42021255763]	Review Ongoing
**11/6/2021**	Transmission of COVID-19 following COVID-19 vaccination: protocol for a rapid review [CRD42021257125]	Review Ongoing
**7/6/2021**	Effectiveness of micronutrient deficiencies interventions among adolescents and women of reproductive age in low middle income countries: a systematic review [CRD42021257267]	Review Ongoing
**23/6/2021**	Consequences of food and nutritional insecurity on family farmers during the COVID-19 pandemic: a systematic review [CRD42021258341]	Review Ongoing
**22/6/2021**	Understanding Covid-19 through the nutritional lens: the syndemic relationship [CRD42021260792]	Review Ongoing
**28/6/2021**	The association between the COVID-19 pandemic and eating behaviours: a systematic review [CRD42021262085]	Review Ongoing
**2/7/2021**	Effectiveness of nutritional interventions on COVID-19 outcomes [CRD42021264900]	Review Ongoing
**14/7/2021**	What are the nutritional rehabilitation strategies after an acute hospital stay among elderly patients? [CRD42021264971]	Review Ongoing
**13/7/2021**	Folate and vitamin B12 and global health - evaluation of policies informed by integrated assessment of health effects [CRD42021265041]	Review Ongoing
**1/12/2022**	A systematic review of the long-term outcomes in patients with juvenile dermatomyositis [CRD42022265132]	Review Ongoing
**5/7/2021**	Association between nutritional status and clinical outcomes among COVID-19 patients in the intensive care unit: a systematic review and meta-analysis [CRD42021265725]	Review Ongoing
**19/8/2021**	Effect of immunomodulators in the treatment of COVID-19 in individuals with and without non-communicable chronic diseases [CRD42021268124]	Review Ongoing
**27/7/2021**	Feeding strategies to prevent neonatal SARS-CoV-2 infection in term or late preterm babies born to mothers with confirmed COVID-19 [CRD42021268576]	Review Ongoing
**4/8/2021**	Association Between Sociodemographic Characteristics on Migraine During the COVID-19 Pandemic [CRD42021270152]	Review Ongoing

**8/6/2022**	Using photography to capture health perceptions: A systematic review of health-related photo-based studies [CRD42022273204]	Review Ongoing
**5/10/2021**	Conservative and non-pharmacological clinical intervention strategies in overweight and obese individuals during the Covid-19 pandemic: a systematic review [CRD42021273904]	Review Ongoing
**8/9/2021**	The effects of consuming a Mediterranean style diet on associated COVID-19 severity biomarkers of obese/overweight adults: a systematic review [CRD42021277070]	Review Ongoing
**15/11/2021**	Breastmilk Exposure and Infant Respiratory Outcomes in High-Risk Offspring: A Systematic Review and Meta Analysis of Individual Patient Data [CRD42021278052]	Review Ongoing
**20/10/2021**	What are the health inequalities associated with emergency care and preventable mortality experienced by African and Caribbean people in Birmingham, Lewisham and the UK? What evidence-based approaches are effective at preventing and addressing these health inequalities? [CRD42021278114]	Review Ongoing
**14/9/2021**	Effect of probiotics, prebiotics and/or vitamin D supplementation on zonulin level, duration, and incidence of respiratory and Covid-19 infections: a systematic review and meta-analysis [CRD42021278712]	Review Ongoing
**10/11/2021**	Effect of the COVID-19 pandemic on the weight of children and adolescents: a systematic review [CRD42021283985]	Review Ongoing
**25/10/2021**	Prevalence and trends in thinness, overweight and obesity amongst children and adolescents aged 2-18 years across Africa, before and during the COVID-19 pandemic: a systematic review and meta-analysis [CRD42021287009]	Review Ongoing
**3/2/2022**	Systematic review of the programs of physical activity used in COVID-19 rehabilitations [CRD42022289219]	Review Ongoing
**11/5/2022**	Association between nitrate and nitrite bioavailability with blood pressure in humans: a systematic review and meta-analysis [CRD42022290244]	Review Ongoing
**3/12/2021**	Impact of COVID-19 pandemic on diet and nutritional status among children; systematic review [CRD42021292373]	Review Ongoing
**10/2/2022**	The impact of the COVID-19 pandemic on rural food security in high income countries: a systematic review [CRD42022296790]	Review Ongoing
**19/1/2022**	Mental Health and Psycho-social support (MHPSS) interventions in infectious disease outbreaks: an umbrella review [CRD42022297138]	Review Ongoing
**20/12/2021**	Impact of covid-19 quarantine on lifestyle-related to nutrition behavior: a systematic review. [CRD42021298620]	Review Ongoing
**4/2/2022**	Effects of preoperative exercise training on clinical outcomes after elective knee surgery: A systematic review and network meta-analysis [CRD42022300792]	Review Ongoing

**10/1/2022**	Intracranial hemorrhage (ICH) among COVID-19 patients, risk factors and consequences: a systematic review [CRD42022302406]	Review Ongoing
**7/2/2022**	Nutrition to assist immune function during post-acute SARS-CoV infections in adults: a systematic review [CRD42022306051]	Review Ongoing
**27/1/2022**	The effects of melatonin on outcomes of COVID patients: a systematic review and meta-analysis of randomized controlled trials [CRD42022306483]	Review Ongoing
**7/2/2022**	What is the optimal choice of severe COVID-19? A systematic review and network meta-analysis [CRD42022308850]	Review Ongoing
**13/3/2022**	Impact of a rounding checklist on the outcomes of patients admitted to an intensive care unit [CRD42022310092]	Review Ongoing
**15/2/2022**	Maternal and neonatal outcomes and COVID-19 in India: a systematic review and meta-analysis [CRD42022310910]	Review Ongoing
**28/2/2022**	Teleworking and occupational health. A systematic review [CRD42022312044]	Review Ongoing
**22/2/2022**	The incidence of seizures in patients receiving COVID-19 vaccines: a systematic review and meta-analysis [CRD42022312475]	Review Ongoing
**31/3/2022**	Health and safety in next generation workspaces: a systematic review of reviews [CRD42022313172]	Review Ongoing
**28/2/2022**	Assessing the Impact of Telehealth Care Interventions for Working Women's' Mental Health during COVID-19 Pandemic. (A systematic review with meta-analysis of its scope, Quality and Implementation) [CRD42022313200]	Review Ongoing
**28/2/2022**	The incidence of facial nerve palsy in patients receiving COVID-19 vaccines: a systematic review and meta-analysis [CRD42022313299]	Review Ongoing
**12/4/2022**	Acute kidney injury in COVID-19 patients receiving remdesivir: a systematic review and meta-analysis of randomized controlled trials [CRD42022313410]	Review Ongoing
**28/2/2022**	Changes of dietary intake during the restrictions of the COVID-19 pandemic: protocol for a systematic review [CRD42022313653]	Review Ongoing
**7/4/2022**	Impact of inpatient multidisciplinary rehabilitation for adults and older adults with COVID-19: A Systematic Review. [CRD42022323534]	Review Ongoing
**13/4/2022**	Nutritional supplementation and clinical outcomes in COVID-19: a systematic review and network meta-analysis [CRD42022324845]	Review Ongoing
**27/4/2022**	The role of vitamin D and other immunonutrients in covid-19 disease: a systematic review [CRD42022328224]	Review Ongoing
**3/6/2022**	Exercise prescription effectiveness offered by health personnel and exercise professionals in 26 different chronic diseases: A Systematic Review and Meta-analysis [CRD42022332289]	Review Ongoing
**18/5/2022**	Stress management interventions for CCRNs during COVID-19: a systematic review and meta-analysis [CRD42022333386]	Review Ongoing

**30/5/2022**	Costs of inpatient care and out of pocket payments for Covid-19 patients: a systematic review [CRD42022334337]	Review Ongoing
**25/5/2022**	Maternal and neonatal safety outcomes after SAR-CoV-2 vaccination during pregnancy: a systematic review and meta-analysis. [CRD42022334425]	Review Ongoing
**25/5/2022**	Efficacy and Safety outcomes after SAR-CoV-2 vaccination in organ transplant recipients: a systematic review and meta-analysis. [CRD42022334852]	Review Ongoing
**7/6/2022**	Vitamin E intake and supplementation and respiratory infections: A Systematic Review and Meta-Analysis [CRD42022335512]	Review Ongoing
**12/6/2022**	Factors associated with body image dissatisfaction in adults: a systematic review and meta-analysis of the literature [CRD42022336758]	Review Ongoing
**8/6/2022**	Type 1 diabetes mellitus and COVID-19 infection: A systematic review of case reports [CRD42022338271]	Review Ongoing
**20/6/2022**	Does telenutrition provide a better solution for patient management during COVID-19? A systematic review of the telenutrition feasibility and effectiveness from major countries [CRD42022340706]	Review Ongoing
**8/7/2022**	An Umbrella Review and Meta-Analysis of Clinical Outcomes for Nutritional Interventions in the Hospital for Adults with Malnutrition or at Risk of Malnutrition [CRD42022341031]	Review Ongoing
**17/8/2022**	New-onset diabetes mellitus in COVID-19: a systematic review [CRD42022341638]	Review Ongoing
**5/7/2022**	Realist Review: Health visiting in light Of the COVID-19 Pandemic Experience (RReHOPE) [CRD42022343117]	Review Ongoing
**12/7/2022**	Covid-19 and Hospital Acquired Pressure Injuries: A Systematic Review [CRD42022343232]	Review Ongoing
**18/7/2022**	Differences between arterial hypertension and obesity in COVID-19 mortality: a systematic review protocol with meta-analysis [CRD42022347203]	Review Ongoing
**6/8/2022**	Digital health in schools: a systematic review [CRD42022349149]	Review Ongoing
**1/8/2022**	Effectiveness of home-based exercise interventions on pain, physical function and quality of life in patients with knee osteoarthritis during the COVID-19 pandemic: a meta-analysis [CRD42022350513]	Review Ongoing
**1/8/2022**	Eating and lifestyle behaviour of adolescents during the COVID-19 pandemic and their risk of becoming overweight and obese: a systematic review [CRD42022350555]	Review Ongoing
**1/9/2022**	The impact of economic growth and recessions on maternal and child health outcomes in Sub-Saharan African countries [CRD42022352086]	Review Ongoing
**5/9/2022**	The impact of Covid-19 pandemic on maternal wellbeing, infant feeding practice, and infant behaviour: a systematic review and meta-analysis [CRD42022354670]	Review Ongoing
**20/9/2022**	An overview of digital healthcare interventions for women's mental health during the COVID-19 pandemic: A systematic review [CRD42022360516]	Review Ongoing

**26/9/2022**	Postmortem histopathological and ultrastructural findings of kidney in COVID-19 patients: a systematic review and meta-analysis [CRD42022362440]	Review Ongoing
**13/10/2022**	Public and patient involvement (PPI) in behavioural interventions for oro- pharyngeal dysphagia RCT's: a systematic review [CRD42022364202]	Review Ongoing
**10/10/2022**	The effect of early enteral nutrition on the clinical outcome for COVID-19 patients requiring mechanical ventilation: a meta-analysis and systematic review [CRD42022365843]	Review Ongoing
**7/11/2022**	Teachers' quality of life perception during the Covid-19 pandemic: A Systematic Review Protocol [CRD42022365861]	Review Ongoing
**7/11/2022**	Perception of quality of life among administrative professionals in educational institutions regarding implications of remote and in-person work: A systematic review protocol [CRD42022365862]	Review Ongoing
**27/10/2022**	The lived experience of food insecurity among adults with obesity: a systematic review. [CRD42022368762]	Review Ongoing
**29/11/2022**	Investigating the extent of dysbiosis in the critically ill [CRD42022372577]	Review Ongoing
**28/11/2022**	Epidemiology of respiratory viruses among children during the SARS-CoV-2-pandemic: a systematic review and meta-analysis [CRD42022379297]	Review Ongoing
**28/1/2023**	Online intervention in mental health of university students: a systematic review. [CRD42023392665]	Review Ongoing
**14/2/2023**	Mortality in patients with Candidemia and COVID-19: a systematic review and meta-analysis [CRD42023399398]	Review Ongoing
**20/2/2023**	A systematic review of the impacts of combined nutrition and exercise interventions on COVID-19 patient rehabilitation [CRD42023400716]	Review Ongoing
**10/3/2023**	Candida lipolytica bloodstream infection in a COVID-19 adult patient with alcohol use disorder: a unique case and a systematic review of literature [CRD42023405326]	Review Ongoing
**13/3/2023**	Effects of quercetin on inflammatory markers of COVID-19 patients: a systematic review and meta-analysis [CRD42023407390]	Review Ongoing
**6/5/2023**	Use of heparin (UFH, LMWH) for the treatment and prevention of thrombosis in children with burdening symptoms who may need palliative care. Systematic review. [CRD42023414353]	Review Ongoing
**5/4/2023**	Prevalence of food insecurity during the covid-19 pandemic in Latin America and the Caribbean: a systematic review with meta-analysis [CRD42023414484]	Review Ongoing
**22/4/2023**	The equity of digital interventions for healthy eating and active living in older adults. An international perspective: a systematic review. [CRD42023416155]	Review Ongoing
**28/4/2023**	Comparing the efficacy of vitamin C and vitamin D and their metabolites in critically ill patients: a Bayesian network meta-analysis [CRD42023417902]	Review Ongoing
**5/5/2023**	Prevalence of eating disorders in undergraduate and graduate students, before, during and after the COVID-19 pandemic: a systematic review [CRD42023421432]	Review Ongoing

**5/5/2023**	Unfinished Nursing Care phenomenon in healthcare settings facing a pandemic: a systematic review [CRD42023422871]	Review Ongoing
**25/5/2023**	Impact of COVID-19 vaccination on the effectiveness of oral antiviral drugs in reducing severe COVID-19: protocol for a systematic review and meta-analysis [CRD42023429232]	Review Ongoing
**13/6/2023**	The role of cash transfers on food insecurity during COVID-19: a systematic review [CRD42023429834]	Review Ongoing
**2/6/2023**	BMI and inflammatory biomarkers as covariates in association with the outcome of COVID-19 disease: Comparison of young adults to elderly patients [CRD42023431156]	Review Ongoing
**9/6/2023**	Can music change health behaviours? A systematic review and meta-analysis. [CRD42023433748]	Review Ongoing
**27/6/2023**	The effects of, and perspectives about, community-based allied health services on hospital admissions and emergency department presentations: a systematic review protocol [CRD42023437013]	Review Ongoing
**14/7/2023**	Effect of telemedicine and telehealth in combination with smartphone apps- or short message services-based intervention on blood pressure in adults: A systematic review and meta-analysis [CRD42023441440]	Review Ongoing
**18/7/2023**	Systematic review: the effects of the COVID-19 pandemic on childhood malnutrition rates in northern Nigeria: a comprehensive analysis [CRD42023443763]	Review Ongoing
**31/10/2023**	Antioxidants and hospitalized critically ill patients with Covid-19: A systematic review [CRD42023443975]	Review Ongoing
**17/7/2023**	The effectiveness of health behaviour interventions in improving university students' mental health during the Covid-19 pandemic: a systematic review and meta-analysis [CRD42023445455]	Review Ongoing
**4/9/2023**	Impact of the COVID-19 pandemic on interprofessional competency development during student clinical placements: protocol for a mixed methods systematic review [CRD42023457890]	Review Ongoing
**30/9/2023**	The relationship between USDA's universal school meals program and diet- and health-related outcomes [CRD42023464854]	Review Ongoing
**20/11/2023**	The impact of nutrition on the development and management of long-COVID: a systematic review [CRD42023472403]	Review Ongoing
**18/10/2023**	The effects of visual framing in Covid-19 public health messages on Covid-19 behavioural and non-behavioural outcomes: a restricted systematic review and meta-analysis [CRD42023473115]	Review Ongoing
**31/10/2023**	Pathogenesis and related factors in post COVID-19 mucormycosis patients with an odontological perspective: a systematic review of case report [CRD42023476051]	Review Ongoing
**28/11/2023**	Artificial Intelligence Quality Standards in Healthcare:A Rapid Umbrella Review [CRD42023477439]	Review Ongoing
**17/11/2023**	The Effects of Isolation and Confinement and Extreme Environments on Human Nutrition - A Systematic Literature Review [CRD42023480161]	Review Ongoing
**6/11/2023**	Cardiovascular Long-Covid symptoms: a systematic review and meta-analysis [CRD42023480206]	Review Ongoing

**28/11/2023**	Nutritional status and impact of COVID-19 associated factors in childhood nutrition in Bangladesh during and after the pandemic: a systematic review [CRD42023482478]	Review Ongoing
**24/11/2023**	A Systematic Review: The Impact of COVID-19 Policy Changes on SNAP and WIC Access, Enrollment, Retention, and Benefit Utilization [CRD42023483486]	Review Ongoing
**30/11/2023**	A Comparative Study of Digital and Traditional Intervention Strategies for Secondary Prevention of Stroke Recurrence [CRD42023484771]	Review Ongoing
**19/12/2023**	Navigating the Impact of COVID-19 on Online Higher Education and Sustainable Development: A Systematic Review of Global Perspectives and Local Implications [CRD42023491235]	Review Ongoing
**1/1/2024**	The impact of Lifestyle Behavior Changes on Mental Health and Well-being in Youth (Aged 10-25): A Systematic Review [CRD42024496044]	Review Ongoing
**20/1/2024**	Family-based pediatric weight management interventions in Latin America and the Caribbean targeting children ages 6–12 years old: A systematic review guided by the RE-AIM framework [CRD42024500664]	Review Ongoing
**16/1/2024**	Efficacy, effectiveness and Safety of seasonal influenza vaccination co-administered with other vaccines: a systematic review and meta-analysis [CRD42024502759]	Review Ongoing
**21/5/2024**	Systematic Literature Review - Treatment of Postural Orthostatic Tachycardia Syndrome (POTS) in children and adults [CRD42024507731]	Review Ongoing
**12/2/2024**	Food policies and their impact on food security: a review and meta-analysis [CRD42024508520]	Review Ongoing
**6/3/2024**	Effectiveness of Nutritional Interventions for Physical and Mental Health and Wellbeing of People with Tuberculosis Undergoing Treatment: A Systematic Review [CRD42024516935]	Review Ongoing
**6/3/2024**	Effectiveness of nutritional interventions for household contacts of people diagnosed with tuberculosis disease: A systematic review [CRD42024517173]	Review Ongoing
**6/3/2024**	Sarcopenic Obesity and its association with Respiratory Outcomes in Adults: a Systematic Review and Meta-analysis [CRD42024518254]	Review Ongoing
**4/3/2024**	Associations between maternal food insecurity and breastfeeding in high-income countries: a systematic review of observational studies [CRD42024519556]	Review Ongoing
**11/3/2024**	Prevalence of nutritional risk in children and adolescents hospitalized with COVID-19: systematic review and meta-analysis [CRD42024522741]	Review Ongoing
**26/3/2024**	Optimal composition of the diet for people receiving treatment for tuberculosis disease: A systematic review [CRD42024524797]	Review Ongoing
**26/3/2024**	Acceptability of nutritional supplementation for people with tuberculosis disease: A systematic review [CRD42024524856]	Review Ongoing

**28/3/2024**	Optimal duration of nutritional supplementation for people with or recovering from TB disease: A systematic review [CRD42024525487]	Review Ongoing
**28/3/2024**	Feasibility of assessing Nutritional Status and managing Undernutrition in People with Tuberculosis Disease and their household contacts: A systematic Review [CRD42024525500]	Review Ongoing
**15/4/2024**	What is the effectiveness of non-pharmacological interventions in management of hospitalised pneumonia outside of critical care settings? [CRD42024535005]	Review Ongoing
**27/8/2024**	Exploring the link between food insecurity and mental health during the COVID-19 pandemic: A systematic review [CRD42024535393]	Review Ongoing
**1/7/2024**	The Effects of Exercise Programs on Cardiopulmonary Function and Signs and Symptoms in Patients with Post-COVID-19: A Systemic Review and Meta-Analysis [CRD42024538786]	Review Ongoing
**3/5/2024**	Prevalence of nutritional risk factors of NCDs in the Eastern Mediterranean Region: a systematic review protocol of observational study [CRD42024538880]	Review Ongoing
**28/5/2024**	Impact of telemedicine in intensive care on patient-relevant and process-relevant outcomes: a systematic review protocol [CRD42024547985]	Review Ongoing
**15/6/2024**	Family-based pediatric weight management interventions for children ages 6-12 living with HIV: A systematic review assessing effectiveness and implementation science outcomes [CRD42024554376]	Review Ongoing
**25/6/2024**	Best practice recommendations for constructing educative and persuasive health and nutrition messages: A rapid review [CRD42024558146]	Review Ongoing
**19/6/2024**	A systematic review of the evidence fat-soluble vitamins provide clinical benefit for patients with COVID-19 [CRD42024558874]	Review Ongoing
**12/9/2024**	Systematic Review of Summer Meal Programs with USDA Covid-19 Waivers [CRD42024560482]	Review Ongoing
**12/10/2024**	Effect of routine monitoring of gastric residual volume (GRV) in intensive care patients [CRD42024574057]	Review Ongoing
**8/9/2024**	A systematic review of the relationship between socioeconomic status and diet quality in young adults [CRD42024576918]	Review Ongoing
**12/8/2024**	Food Insecurity, COVID-19, and Disordered Eating Among Undergraduate Students: A Systematic Review [CRD42024577588]	Review Ongoing
**11/9/2024**	A systematic review and meta-analysis of longer-term outcomes after Stroke-Associated Pneumonia (SAP) [CRD42024587435]	Review Ongoing
**30/9/2024**	ASH/ISTH Pharamlogical Prophylaxis Guideline Pediatric Venous Thromboembolism [CRD42024591025]	Review Ongoing
**16/10/2024**	Plant-based diets for older adults in care homes: A realist systematic review [CRD42024600852]	Review Ongoing

**29/10/2024**	Diet Quality and Nutrition Outcomes in Adolescents: A Systematic Review of Studies in Nigeria [CRD42024603518]	Review Ongoing
**16/12/2024**	Determinants of Maternal and Infant Nutrition and Growth in Global Exemplar Countries [CRD42024619766]	Review Ongoing
**18/12/2024**	Systematic Review of Food and Nutrition Programs and Policies for Adolescent Sexual and Reproductive Health [CRD42024623830]	Review Ongoing
	**COVID-19 and nutrition and probiotics**	
**16/3/2021**	The impact of gut microbiota and modulators on lung immunity and pathogenicity of respiratory tract infections: an integrated systematic review [CRD42021242779]	Review Ongoing
**28/5/2021**	The impact of gut microbiota and modulators on lung immunity and pathogenicity of respiratory tract infections in animal models and in vitro systems: an integrated systematic review and meta-analysis [CRD42021242785]	Review Ongoing
**28/8/2021**	Supplementation therapy to minimise nutrition- and diet-related complications of bariatric surgery. [CRD42021244336]	Review Ongoing
**22/6/2021**	Understanding Covid-19 through the nutritional lens: the syndemic relationship [CRD42021260792]	Review Ongoing
**14/7/2021**	What are the nutritional rehabilitation strategies after an acute hospital stay among elderly patients? [CRD42021264971]	Review Ongoing
**14/9/2021**	Effect of probiotics, prebiotics and/or vitamin D supplementation on zonulin level, duration, and incidence of respiratory and Covid-19 infections: a systematic review and meta-analysis [CRD42021278712]	Review Ongoing
	**COVID-19 and probiotics**	
**7/11/2024**	Effectiveness of Probiotics on COVID-19 Prevention and Treatment Against Mild COVID-19 in Outpatient Care: A Systematic Review [CRD42024611230]	Review Ongoing
**24/8/2024**	Probiotics Influencing Metabolism and Microbial Diversity in Patients with Depression: A Systematic Review And Meta-analysis of Randomized Controlled Trials [CRD42024577652]	Review Ongoing
**3/8/2024**	Effects of isolation and confinement on gastrointestinal symptoms- a systematic review [CRD42024540150]	Review Ongoing
**29/4/2024**	Traditional, Complementary, and Integrative Medicine therapies for the treatment of mild/moderate acute COVID-19: a systematic review and network meta-analysis [CRD42024517321]	Review Ongoing
**16/2/2024**	Preventive interventions for post Covid-19 condition: protocol for systematic review update [CRD42024513247]	Review Ongoing

**21/3/2024**	Influence of the use of prebiotics and probiotics on the course of COVID-19: a systematic review [CRD42024472465]	Review Ongoing
**24/4/2023**	Assessment of the prophylactic effect of probiotics against COVID-19 infection [CRD42023418900]	Review Ongoing
**15/4/2023**	The use of probiotics as adjuvant therapy in the treatment of respiratory infections in children and adolescents: a systematic review and meta-analysis [CRD42023414402]	Review Ongoing
**6/4/2023**	Role of prebiotics in bacterial and viral infection, and vaccination efficiency: A systematic review [CRD42023408335]	Review Ongoing
**9/1/2023**	Clinical efficacy and safety of molnupiravir for non-hospitalized and hospitalized patients with COVID-19: a systematic review and meta-analysis of randomized control trials [CRD42023390315]	Review Ongoing
**9/1/2023**	Probiotics for the treatment of COVID-19 patients: a meta-analysis [CRD42023390275]	Review Ongoing
**19/12/2022**	Effectiveness and safety of Traditional, Complementary and Integrative Medicine on fatigue post COVID-19 infection: a systematic review of randomized controlled trials [CRD42022384136]	Review Ongoing
**6/12/2022**	Effect of probiotics on the COVID-19: a systematic review and meta-analysis of randomized controlled trials [CRD42022381061]	Review Ongoing
**24/10/2022**	Traditional, complementary and integrative medicine for the treatment of skin diseases related to coronavirus infection: a systematic review [CRD42022369434]	Review Ongoing
**5/8/2022**	Assessment of Psychobiotics Efficacy on Patients with Depressive Disorder: A Comprehensive Systematic Review and Meta-Analysis of Randomized Controlled Trials [CRD42022348953]	Review Ongoing
**14/7/2021**	What are the nutritional rehabilitation strategies after an acute hospital stay among elderly patients? [CRD42021264971]	Review Ongoing
**22/6/2021**	Understanding Covid-19 through the nutritional lens: the syndemic relationship [CRD42021260792]	Review Ongoing
**28/8/2021**	Supplementation therapy to minimise nutrition- and diet-related complications of bariatric surgery. [CRD42021244336]	Review Ongoing
**16/3/2021**	The impact of gut microbiota and modulators on lung immunity and pathogenicity of respiratory tract infections: an integrated systematic review [CRD42021242779]	Review Ongoing
**22/6/2020**	Probiotics for treating novel coronavirus (COVID-19) with diarrhea: a systematic review and meta-analysis [CRD42020192657]	Review Ongoing

**12/6/2020**	The impact of COVID-19 on intestinal flora:a protocol for systematic review and meta analysis [CRD42020191640]	Review Ongoing
**21/4/2020**	The effects of synbiotics, probiotics, and prebiotics supplementation on COVID-19 manifestations and other viral infections: a systematic review and meta-analysis of clinical trials [CRD42020181453]	Review Ongoing
**14/10/2021**	Efficacy of neutraceuticals (probiotics or prebiotics or synbiotics) in the prevention or treatment of COVID -19: a systematic review and meta-analysis [CRD42021284923]	Review Ongoing
**14/9/2021**	Effect of probiotics, prebiotics and/or vitamin D supplementation on zonulin level, duration, and incidence of respiratory and Covid-19 infections: a systematic review and meta-analysis [CRD42021278712]	Review Ongoing
**8/12/2021**	The effectiveness of interventions to reduce the transmission of acute respiratory infections in care homes: a systematic review [CRD42021292849]	Review Ongoing
**18/11/2021**	Preventing Respiratory Tract Diseases by probiotic Interventions: A Systematic Review and Meta-Analysis of Randomized Controlled Trials [CRD42021292142]	Review Ongoing
